# *In vivo* genome editing of central nervous system SIV reservoirs in ART-suppressed rhesus macaques

**DOI:** 10.1016/j.omtn.2026.103063

**Published:** 2026-08-14

**Authors:** Hong Liu, Shuren Liao, Chen Chen, Wenwen Huo, Zahra Safaei, Angela Rocchi, Yuru Huang, Ilker K. Sariyer, Mackenzie E. Collins, Jill M. Lawrence, Will Dampier, Michael R. Nonnemacher, Brian Wigdahl, Tricia H. Burdo, Jennifer Gordon, Mark Lewis, Cheri A. Lee, Kamel Khalili

**Affiliations:** 1 Center for Neurovirology and Gene Editing, Department of Microbiology, Immunology and Inflammation, Lewis Katz School of Medicine at Temple University, 3500 N. Broad Street, 7th Floor, Philadelphia, PA 19140, USA; 2 Excision BioTherapeutics, Inc., 499 Jackson Street, San Francisco, CA 94111, USA; 3 Center for Molecular Virology and Translational Neuroscience, Institute for Molecular Medicine and Infectious Disease, Department of Microbiology and Immunology, Drexel University College of Medicine, 2900 W Queen Ln, Philadelphia, PA 19129, USA; 4 Rutgers Institute for Translational Medicine and Science, Robert Wood Johnson Medical School, Rutgers, The State University of New Jersey, 89 French Street, Suite 4275, New Brunswick, NJ 08901, USA; 5 Bioqual, Inc., 9600 Medical Center Drive, #101, Rockville, MD 20850, USA

**Keywords:** MT: RNA/DNA editing, SIV, HIV, CRISPR-Cas9, excision, viral reservoirs, viral persistence, brain regions, ART, CNS infection

## Abstract

Latent human immunodeficiency virus type 1 (HIV-1) reservoirs in the central nervous system (CNS) may sustain viral persistence and neuroinflammation contributing to HIV-associated neurocognitive disorders (HAND) despite suppressive ART. AAV9-delivered CRISPR has successfully edited SIV proviral DNA in peripheral tissues with acceptable safety profiles, but the extent of *in vivo* genome editing in the brain remains unclear. Using SIV-infected rhesus macaques, we mapped intact proviral DNA across CNS regions and tested systemic AAV9-CRISPR-Cas9 targeting conserved sites within Ψ packaging signal and Gag region. Ten adult rhesus macaques were infected with genetically barcoded SIVmac239, suppressed with ART, then randomized to receive intravenous AAV9-SaCas9 with dual gRNAs (Ψ + Gag) or a Cas9-only control. At necropsy after viral rebound, SIV genomes were detected in multiple brain regions as well as lymphoid tissues, confirming the CNS as a persistent reservoir during ART. Barcode analysis revealed region-specific patterns consistent with compartmentalized CNS persistence. In CRISPR-treated animals, proviral editing was measurable across anatomically distinct CNS sites. These findings demonstrate that intact and potentially replication-competent virus persists in the primate brain under ART and that systemic AAV9-CRISPR can reach and edit proviral DNA in this sanctuary, supporting genome editing as a strategy toward durable remission of CNS reservoirs.

## Graphical abstract

**Figure:**
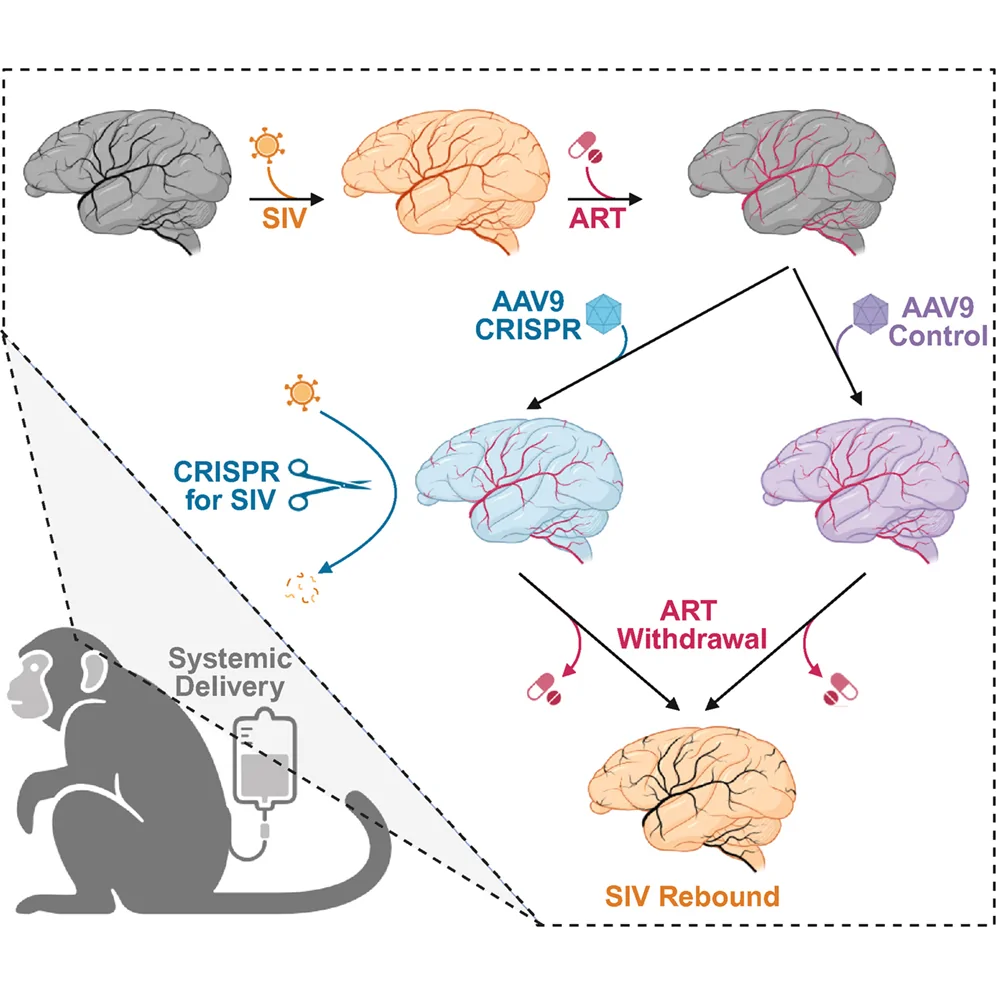

## Teaser

Khalili and colleagues show that the brain remains a persistent viral reservoir despite ART, with intact proviral DNA detected across CNS regions in SIV-infected macaques. Their findings demonstrate that systemic CRISPR gene editing can access these sanctuary sites, advancing strategies toward durable HIV remission.

## Introduction

Combination antiretroviral therapy (ART) continuously suppresses human immunodeficiency virus type 1 (HIV-1) replication but does not eradicate the virus because of the persistence of dormant or minimally replicating viral reservoirs.^1^^,^^2^ These reservoirs are established at the early stage of infection and can drive rapid viral rebound and viremia when therapy is interrupted.^3^^,^^4^ Among the anatomical reservoir sites, the central nervous system (CNS) represents a particular challenge owing to restricted immune surveillance, limited drug penetration across the blood-brain barrier (BBB), and viral discordance between plasma and cerebrospinal fluid (CSF).^5^^,^^6^ Infection with HIV-1 is frequently associated with neurological complications, most notably HIV-associated neurocognitive disorders (HANDs), which remain prevalent even in individuals receiving effective ART.^7^^,^^8^ Although early concepts attributed these disorders primarily to indirect effects of systemic infection and the accompanying inflammatory response, subsequent studies have increasingly supported direct CNS involvement through viral entry across the BBB and infection of resident brain cells.^9^^,^^10^

Within the brain, tissue-resident macrophages and microglia are considered major cellular reservoirs that can harbor replication-competent viruses, while astrocytes and other non-classical brain cell types may also be infected, in some cases in a restricted or abortive manner. And such infection can disrupt neuronal function and behavior through persistent inflammation, altered glial support, and local neurotoxicity, thereby contributing to neuronal injury, cognitive decline, dementia, and broader neurological dysfunction.^11^^,^^12^ These observations raise an important question: can the brain serve as a clinically relevant viral reservoir that sustains persistent proviral DNA despite systemic viral suppression? Early *in situ* and postmortem studies have provided evidence for CNS cellular reservoirs, including detection of HIV-1 nucleic acids in microglia, macrophages, astrocytes, and, in rare instances, neurons.^13^^,^^14^ These findings underscore the brain as a clinically relevant sanctuary site that may sustain latent or low-level viral persistence during ART.

Gene editing offers a promising strategy to directly eliminate integrated proviral DNA. CRISPR-Cas9 systems can be programmed to excise conserved viral sequences and have shown antiviral activity in cell culture and animal models of HIV infection.^15^^,^^16^^,^^17^^,^^18^ Effective translation of this approach to the CNS, however, requires delivery systems with broad biodistribution and the ability to access brain tissue. Adeno-associated virus serotype 9 (AAV9) has been shown to cross the BBB and transduce neurons and glia in rodents and nonhuman primates, motivating its use for CNS-directed genome editing.^19^ In our previous studies using ART-treated rhesus macaques, AAV9-delivered CRISPR has edited SIV proviral DNA in peripheral tissues with an acceptable safety profile, yet its editing activity across defined CNS regions has not been comprehensively assessed.^20^^,^^21^

The SIV-infected rhesus macaque is regarded as a gold-standard preclinical model for HIV-1 infection because it closely models viral pathogenesis, reservoir biology, and tissue distribution, enabling rigorous evaluation of cure strategies *in vivo*.^22^^,^^23^ In addition, genetically barcoded SIVmac239 provides a unique opportunity to examine the clonal composition and diversity of viral reservoirs across tissues.^24^ Here, using barcoded SIV in long-term ART-suppressed macaques, we investigated the presence of SIV proviral DNA in multiple regions of the brain and evaluated the potential of systemic AAV9-CRISPR-Cas9 delivery to edit conserved SIV sequences within the Ψ packaging signal and Gag region in CNS reservoirs elements and assess regional CNS proviral editing alongside clonal dynamics. Further comparison of the genetic diversity and clonal architecture of viral reservoirs in the brain versus peripheral compartments provides insight into the brain as a potential reservoir site and informs gene-editing strategies aimed at viral elimination from the CNS.

## Results

### Design and validation of CRISPR-Cas9 gRNAs

**Figure 1:**
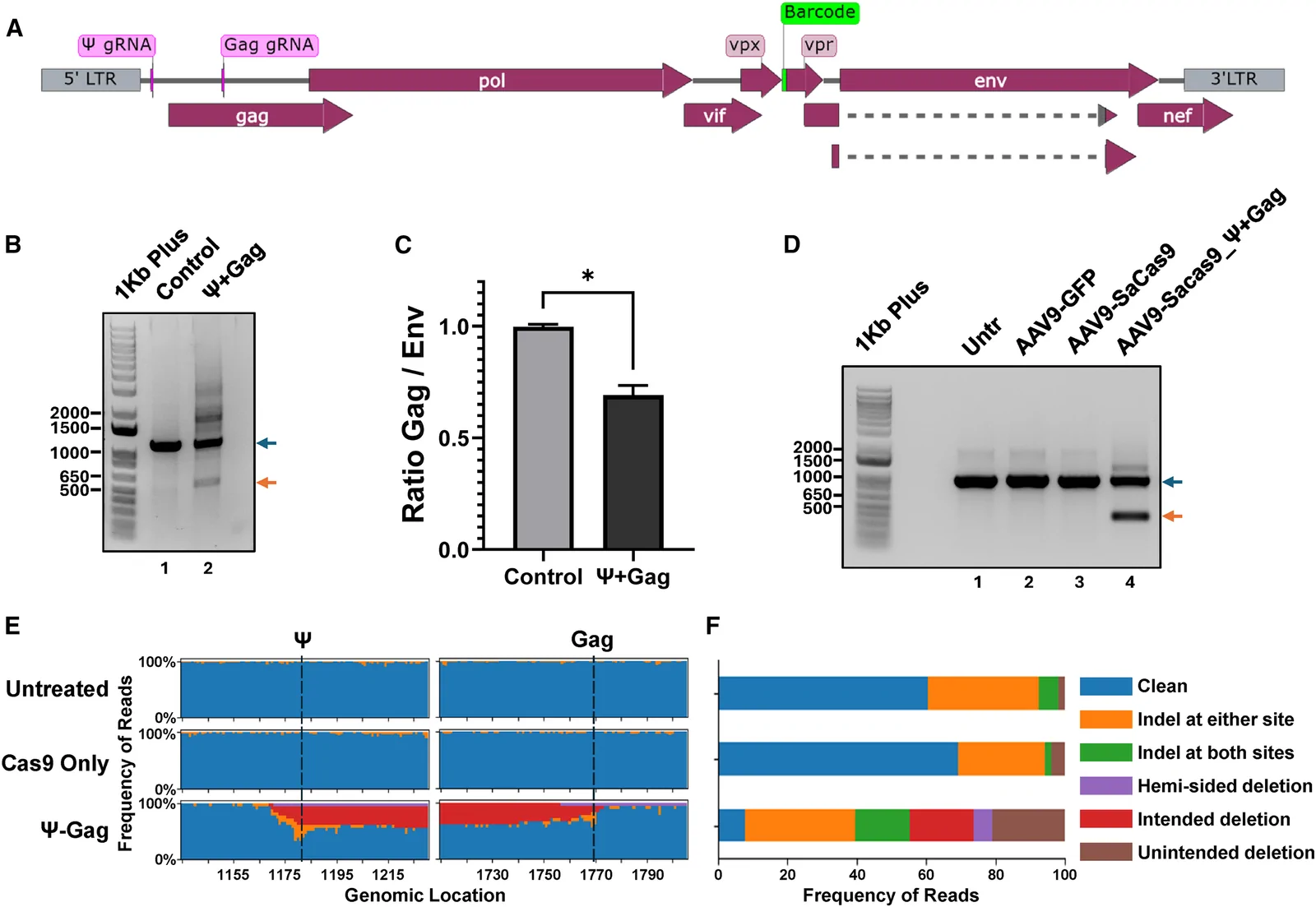
Design and validation of gRNAs targeting the SIV genome (A) Schematic of the SIV genome showing the locations of the Ψ (psi) and Gag gRNA target sites and the barcode region. (B) PCR across the Ψ-Gag interval using DNA from 3D8 cells electroporated with Cas9 RNPs carrying both gRNAs. Dual-gRNA editing generated a smaller amplicon consistent with excision of the 590-bp Ψ-Gag segment (the orange arrow indicates the excision band, and the blue arrow indicates the full-length band). (C) Excision efficiency quantified by ddPCR. A reduced Gag-to-Env signal ratio indicated deletion of the SIV Gag-containing segment in 3D8 cells. Data are presented as mean ± SD. ∗*p* < 0.05. (D) Nested PCR confirmation of Ψ-Gag excision in AAV9-CRISPR-treated 3D8 cells, showing a truncated product consistent with deletion (the orange arrow indicates the excision band, and the blue arrow indicates the full-length band). (E) The frequency of clean (blue), small indel (orange), hemi-sided deletions (purple), intended deletion (red), or unintended deletion (brown) at each base surrounding the target sites. The dashed line indicates the targeted cut sites. (F) A stacked bar plot of the relative frequency of classified reads.

To identify optimal target sequences for SIV gene editing, the *in silico* analyses were performed to design gRNAs against conserved regions of the SIV genome. The LTR-targeting gRNA used in our previous studies was excluded to preserve the barcode cassette required for downstream clonal tracking analyses. The candidate gRNAs were evaluated using *CRISPOR* and *Benchling* platforms for bioinformatic predictions of on-target activity and off-target risk. Two high-scoring gRNAs targeting Ψ_112 and Gag_468 were selected for the downstream studies (Figure 1A). The 3D8 cell line, which harbors a single integrated SIV copy per cell, was used to functionally verify the activity of gRNAs via SaCas9-CRISPR ribonucleoprotein (RNP) nucleofection. The dual-gRNA SaCas9 complex electroporated into 3D8 cells resulted in the expected Ψ-Gag segment deletion as verified by excision PCR (Figure 1B) and Sanger DNA sequencing (Figure S1). Based on the reduction in the Gag-to-Env signal ratio detected by droplet digital PCR (ddPCR), excision of Gag-containing segment occurred in approximately 30% of total SIV genomes (Figure 1C). Additionally, the macaque microglia cells M1A infected with SIVmac239 were used to test the CRISPR-Ψ-Gag dual gRNA system and showed editing outcomes similar to those observed in 3D8 cells (Figure S2). The two gRNAs were cloned into the pX601 vector, and SaCas9 expression was placed under the control of the EF1α promoter. This modification preserved SaCas9 expression at levels comparable to those achieved with the original CMV promoter while reducing vector size to better accommodate AAV9 packaging limitations (Figure S3). The AAV9 vector containing SaCas9 and two gRNAs were tested in the 3D8 cells. PCR analysis confirmed successful cleavage at the Ψ and Gag gRNA sites, resulting in deletion of the intervening SIV genomic segment (Figure 1D). To further confirm the impact of the Ψ and Gag in the 3D8 cells, SIV-near full length (NFL) PCR, which amplifies an approximate 7 kb segment of the proviral genome, was used to quantify the rate of small indels and targeted deletions generated. Each UMI-consensus read was aligned and classified as either clean, contained an indel, or was part of a small indel or large deletion. A pattern of indels and targeted deletions were observed at the target sites in treated samples (Figure 1E). In the Ψ-Gag treated samples, only 7.9% of the reads had no evidence of edits vs. 60% and 69% in untreated or Cas9 only controls, respectively. The frequency of reads containing an indel at either site was consistent across samples but the number of reads with indels at both sites was 15% in treated vs. 5.66% and 1.9% in untreated or Cas9 only controls, respectively. The site-specific excision event (targeted deletion) was found in 18% of reads from Ψ-Gag treated samples and was not observed in either control. Interestingly, we observed that 5% of reads contained a deletion where only one edge matched a target while the other edge did not. This was only observed in the Ψ-Gag treated samples and never observed in either control. There was also an increase in the frequency (21%) of unintended proviral deletions, where neither end matched a target site in Ψ-Gag treated samples, compared to only 1.8% and 2.8% in untreated or Cas9 only controls, respectively (Figure 1F). These results support the activity of the selected gRNA pair and provide a rationale for its subsequent evaluation *in vivo*.

### *In vivo* AAV9-CRISPR-Cas9 administration in SIV-infected rhesus macaques

**Figure 2:**
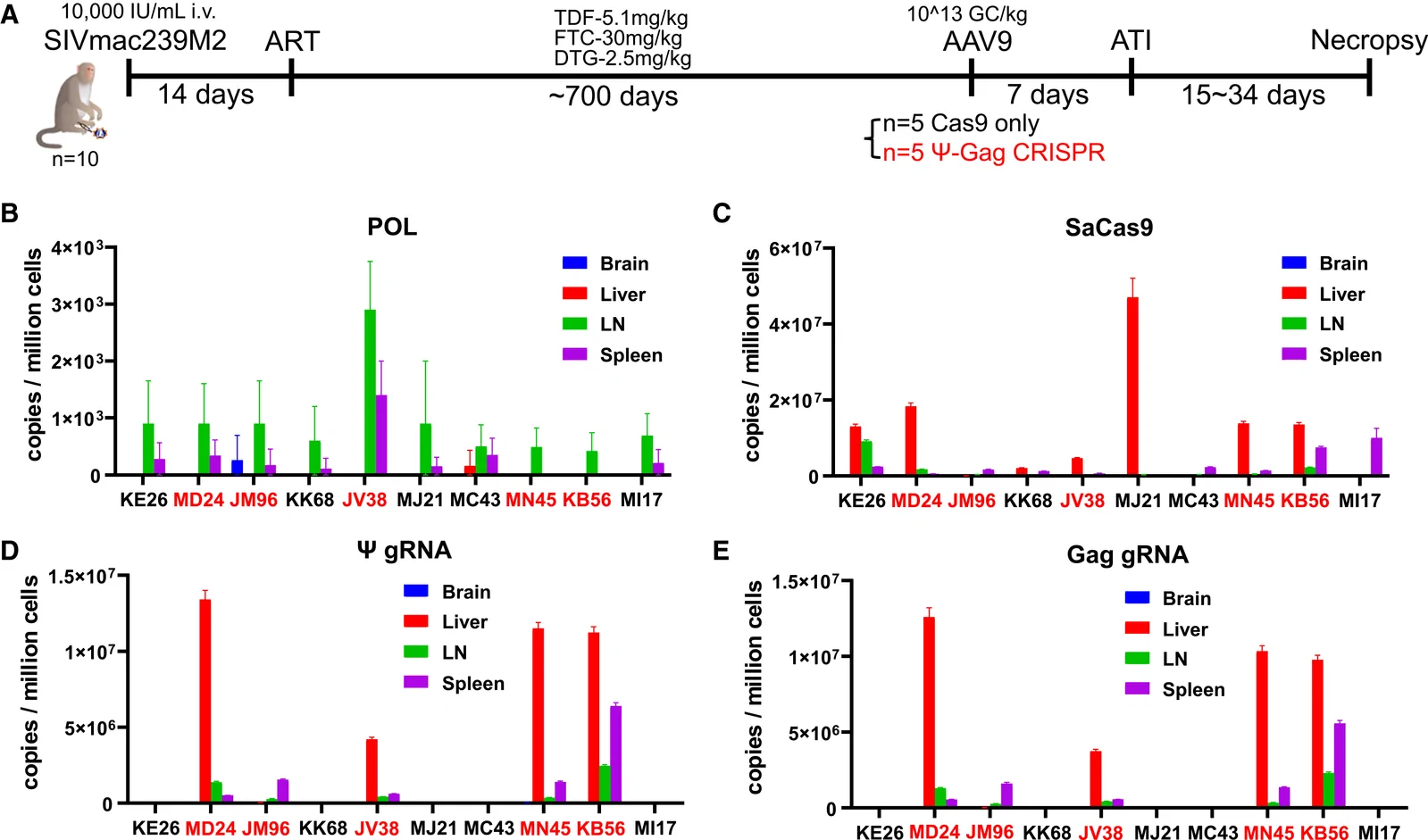
SIV and CRISPR detection in AAV9-CRISPR-Cas9 treated monkeys (A) Timeline illustrating experimental design wherein ten rhesus macaques were intravenously inoculated with barcoded SIVmac239M2 and maintained on daily subcutaneous ART (tenofovir, emtricitabine, and dolutegravir) for about 24 months. Five animals (MD24, JM96, JV38, MN45, and KB56 shown in red) received a slow intravenous infusion of AAV9 vector delivering CRISPR-Cas9 with dual gRNAs targeting Ψ and Gag (10^13^ GC/kg); the remaining five animals (KE26, KK68, MJ21, MC43, and MI17 shown in black) received a control AAV9 vector expressing Cas9 only. ART was withdrawn one-week post-infusion, and necropsies were performed at the onset of detectable viral rebound. Regionally extracted DNA from all ten animals was quantified by ddPCR for SIV Pol (B), SaCas9 (C), gRNA Ψ (D) and gRNA Gag (E) levels. Data in panels (B)–(E) are presented as mean ± SD.

Following SIVmac239M2 infection and long-term ART suppression, ten rhesus macaques received AAV9 vectors encoding CRISPR-Cas9 with (five Ψ-Gag CRISPR) or without (five Cas9 only) the validated gRNAs (Figure 2A). ART was discontinued one week after AAV9 infusion to initiate analytical treatment interruption (ATI) and assess viral rebound. Following viral rebound, tissues were collected by anatomical regions from which DNA was prepared for the next-step analyses. The ddPCR analysis showed that SIV Pol signals were most prominent in lymph nodes (Figure 2B). While SaCas9 signals, which indicate AAV9 vector delivery and persistence of vector-associated sequences, were enriched primarily in the liver but were also detected across multiple other tissues (Figure 2C). Notably, SaCas9 signal levels were substantially higher than SIV Pol signals in the tissues analyzed. The Ψ (Figure 2D) and Gag (Figure 2E) gRNA sequences were found exclusively in the five animals treated with AAV9-CRISPR.

### SIV proviral editing in multiple tissues of AAV9-CRISPR-treated rhesus macaques

**Figure 3:**
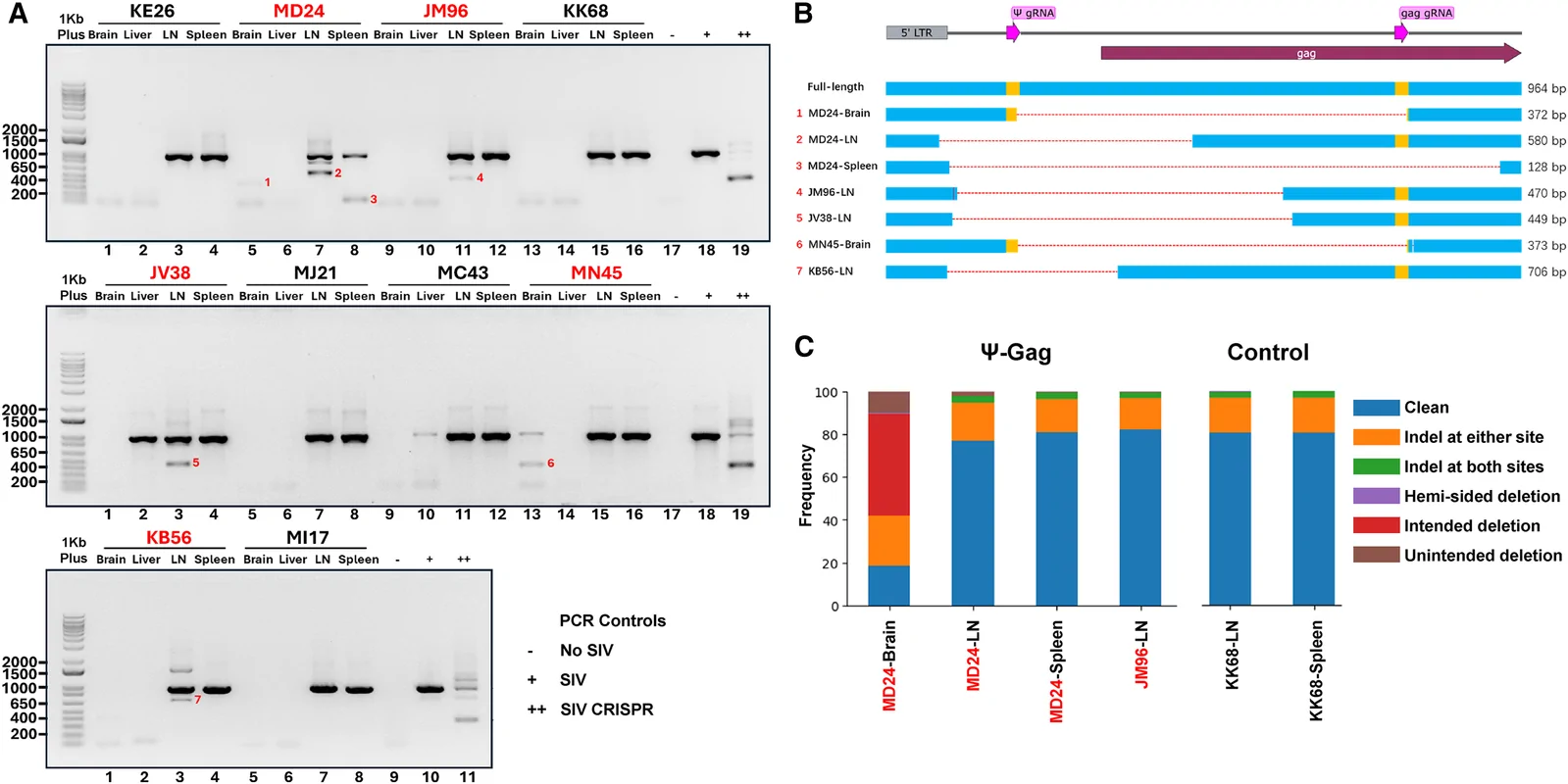
*In vivo* SIV excision by AAV9-delivered CRISPR-Cas9 in ART-suppressed rhesus macaques (A) Agarose gel electrophoresis of nested Ψ-Gag PCR amplicons confirmed SIV proviral DNA excision in tissues from CRISPR-treated animals (MD24, JM96, JV38, MN45, and KB56 shown in red), but not in control animals (KE26, KK68, MJ21, MC43, and MI17 shown in black). The + and ++ represent 3D8 cells and 3D8 cells treated with CRISPR respectively. (B) Truncated amplicons were validated by Sanger sequencing as shown in the sequence alignment. (C) A stacked barplot of the relative frequency of classified reads from the entire PCR reactions shown in (A) as determined by nanopore sequencing.

CRISPR mediated removal of SIV DNA spanning Ψ to Gag was assessed by nested PCR. SIV deletions within this region were detected across tissues from five Ψ-Gag CRISPR-treated monkeys but were absent in Cas9-only control animals, underscoring the requirement of gRNA inclusion (Figure 3A). These deletion products were detected most frequently in lymph node tissues, consistent with the prominent SIV Pol signals observed in lymph nodes by ddPCR (Figure 2B). In contrast, tissues with strong SaCas9 signals but low or undetectable SIV DNA, such as the liver in most animals, did not show detectable editing by PCR-based assays. These findings indicate that detectable proviral editing depends on the simultaneous presence of both the CRISPR editing machinery and sufficient SIV DNA substrate within the sampled tissue. Besides, nested PCR targeting the SIV Pol region did not detect any deletion products in tissues from either CRISPR-treated or control animal (Figure S4), suggesting that the deletions observed in the Ψ-Gag region were specifically mediated by AAV9-delivered CRISPR. Moreover, rhAmpSeq analysis detected no statistically significant increase in indels or structural variants at any nominated predicted off-target site in CRISPR-treated animals compared with control animals, suggesting that editing with the Ψ_112 and Gag_468 guide pair did not produce detectable off-target chromosomal alterations within the analyzed sites and assay sensitivity, thereby supporting the safety profile of the AAV9-CRISPR strategy (Figure S5; Table S1). Sanger sequencing of excised amplicons confirmed SIV editing in these gRNA-treated tissues, while SIV cleavage in the brain tissues showed particularly precise on-target junctions consistent with the predicted CRISPR cut sites (Figure 3B), potentially reflecting greater co-occurrence of CRISPR components and SIV DNA substrate within those sampled tissues. To further confirm these results, the PCR products were subjected to nanopore sequencing to determine the frequency of targeted deletions from treated and untreated animals. The site-specific excision deletion was found in 47% of reads from MD24-Brain and 0% in all other tested samples. The frequency of hemi-targeted deletions and unintended proviral deletions also increased to 0.5% and 10%, respectively, when compared to 0.03% and 0.3% rates seen in the untreated KK68 sample (Figure 3C). These findings motivated a focused investigation of nervous system compartments from all these animals in the following study.

### Distribution of SIV and Cas9 in various brain regions

**Figure 4:**
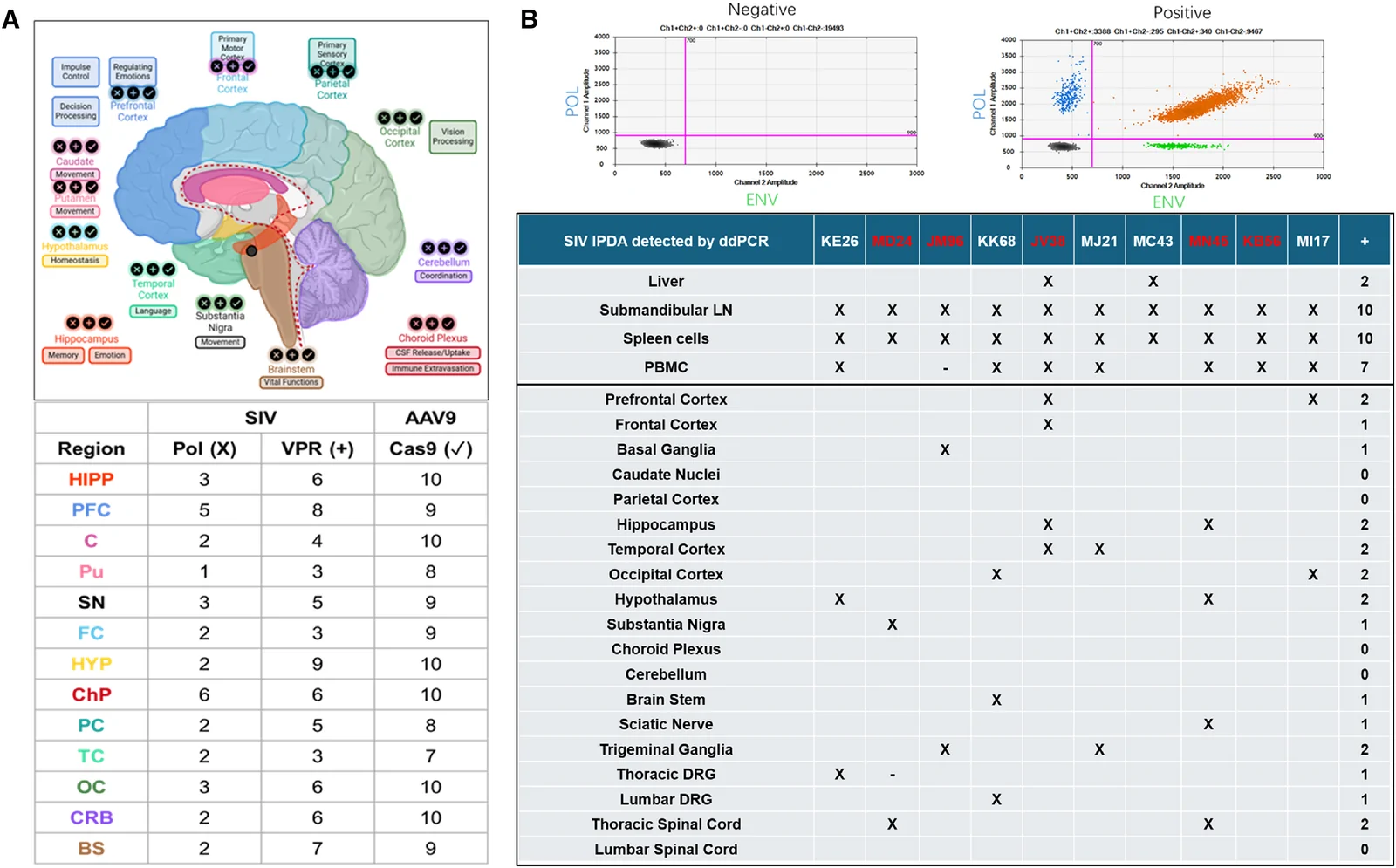
Detection of SIV proviral DNA and SaCas9 across distinct brain regions (A) Schematic illustration of 13 anatomically distinct brain regions (top) from which DNA was extracted and analyzed by ddPCR with accompanying table summarizing the number of animals showing positive signals for SIV Pol, SIV Vpr, and SaCas9 in each region (bottom). (B) Representative IPDA analysis shows that multiplex ddPCR simultaneously amplifies the Pol and Env regions, enabling selective detection of intact proviruses while excluding hypermutated or deleted SIV DNA molecules (top). IPDA was performed on total DNA extracted from various tissues of all ten monkeys. Intact SIV proviral DNA was consistently detected in lymph nodes (LNs) and spleen, as well as in several CNS regions (bottom, “x” indicates positive detection of intact proviral DNA, and “–” indicates not determined; CRISPR-treated animals are shown in red and control animals are shown in white).

Total 13 brain regions from each animal were examined for SIV Pol, Vpr, and SaCas9 DNA. SIV Pol was most frequently detected in prefrontal cortex and choroid plexus, while Vpr was most frequent in hypothalamus and prefrontal cortex (Figure 4A). Both SIV markers were broadly distributed across the brain tissues, as all ten animals show SIV positivity in at least two or three brain regions (Table S2). SaCas9 DNA was detected in over 80% of sampled brain regions, indicating widespread vector access. Using intact proviral DNA assay (IPDA), intact SIV genomes were identified in classical viral enrich sites (e.g., lymph nodes and spleen) as well as in CNS regions including the prefrontal cortex and hypothalamus (Figure 4B; Table S3). However, the intact full-length viral genomes were relatively rare in brain tissues, suggesting that a substantial proportion of CNS-associated viral DNA may be defective and contain deletions. Thus, assays targeting different regions of the SIV genome, such as Pol and Vpr, may yield non-concordant detection patterns, as shown in Figure 4A. These data suggest the presence of replication-competent viruses in various brain regions despite ART intervention, which may serve as an additional reservoir alongside traditional lymphoid tissues.

### CRISPR-Cas9 excises SIV provirus in the nervous system

**Figure 5:**
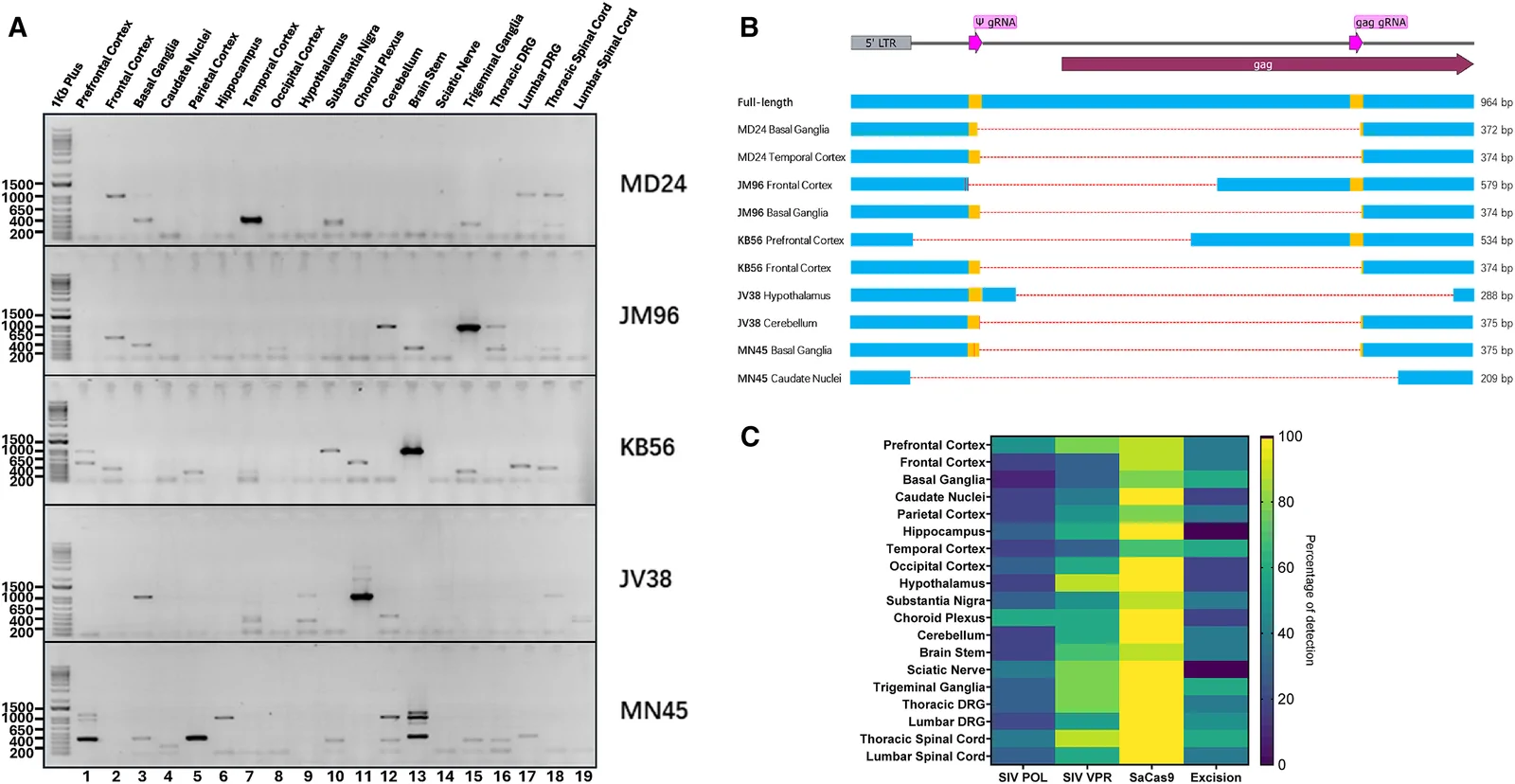
CRISPR-Cas9 mediated excision of SIV DNA in the nervous system of rhesus macaques (A) PCR-based analysis detected excision of SIV proviral DNA (between Ψ and Gag) in various nervous system regions of the five CRISPR-treated animals, demonstrating successful *in vivo* genome editing. The approximately 1,000 bp band represents the full-length amplicon. The Ψ-Gag deletion is indicated by the presence of the smaller excision band of approximately 400 bp. Bands below 100 bp were considered primer dimers. (B) Representative truncated amplicons were confirmed by Sanger sequencing, as shown in the sequence alignment. (C) Heatmap summarizing the distribution of SIV Pol, Vpr, Cas9, and confirmed excision events across nervous system tissues from the five treated animals.

In the five Ψ-Gag CRISPR treated animals, 19 nervous system tissues per animal, including 13 brain, two spinal cord, and four PNS regions, were analyzed for excision-specific amplicons. Some nervous system tissues from these treated animals yielded truncated amplicons when detected by nested PCR (Figure 5A). Sanger sequencing confirmed cleavage mostly located at the Ψ and Gag target sites as shown in the representative schematic, which is consistent with previous findings (Figure 5B). To relate proviral editing to regional SIV and AAV levels, excision events were enriched in CNS areas with higher local SIV and SaCas9 DNA—most notably the prefrontal cortex and substantia nigra (Figure 5C), indicating that editing was influenced by both vector biodistribution and substrate abundance. In addition, no significant correlation was observed between necropsy timing and editing outcomes across animals, suggesting that the modest variation in necropsy timing did not account for the observed editing patterns.

### Barcode analysis of viral diversity

**Figure 6:**
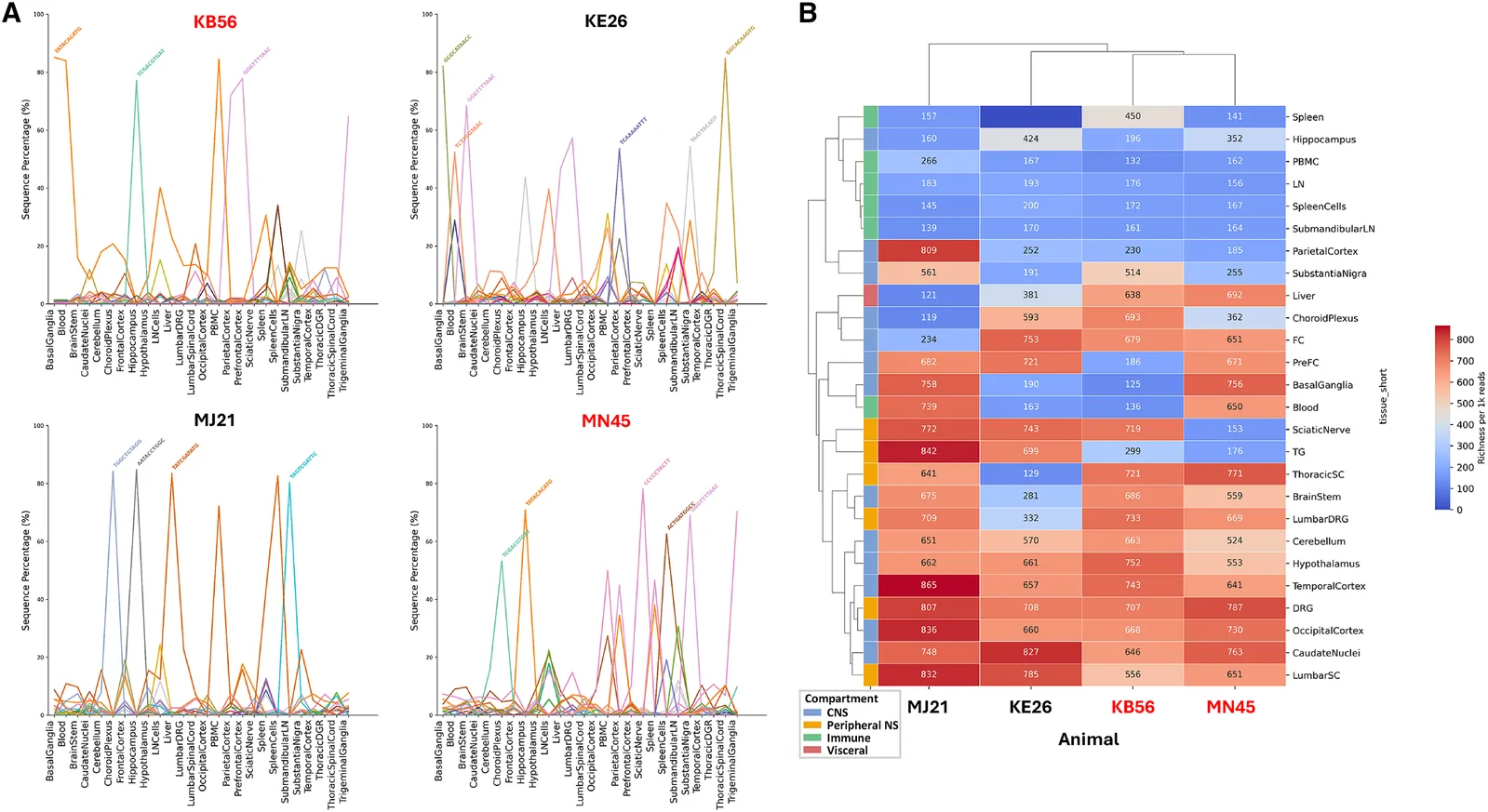
Barcode analysis of viral clonal diversity (A) Relative abundance of the top-10 most abundant barcode sequences per tissue, shown separately for each animal. For each tissue, the ten barcodes with the highest read counts were identified, and their read percentages were plotted across all tissue types. Each line represents an individual barcode sequence, and colors distinguish barcodes within each figure. Those with >50% sequence percentage were annotated with the first 10 nt barcode sequences. (B) Cluster map of normalized barcode richness across 26 tissues and four animals. Tissues and animals were independently clustered using hierarchical clustering with Euclidean distance and average linkage. Cell colors represent normalized richness values according to the color scale shown (blue: low, red: high). Numerical values indicate the raw rounded integer values of normalized richness. The dendrogram on the top illustrates similarity among animals, while the dendrogram on the left illustrates similarity among tissues based on their barcode richness profiles. Colored bars alongside the tissue dendrogram indicate anatomical compartment: CNS (blue), peripheral nervous system (amber), immune/lymphoid (green), and visceral (red). KE26 Spleen had no available data (shown as 0). Untreated animals shown in black: MJ21, KE26. CRISPR-treated animals shown in red: KB56, MN45.

To investigate SIV clonal dynamics, barcode-tagged sequences were analyzed in various tissues from 4 monkeys (controls: MJ21, KE26; treated: KB56, MN45). High-throughput sequencing revealed limited but distinct populations within individual regions, suggesting that CNS reservoirs can be seeded and maintained at least partially independent from viral persistence in peripheral sites (Figure 6A). Notably, the normalized barcode richness was much lower in peripheral tissues associated with higher rebound virus levels (e.g., PBMC, lymph nodes, spleen) than in nervous system tissues (Figure 6B). These findings illustrate spatial compartmentalization of reservoir populations and support the concept that anatomically distinct reservoirs may evolve uniquely under local constraints and contribute differentially to rebound after ATI.

## Discussion

Despite decades of research and the advent of potent ART, the eradication of HIV remains elusive due to the persistence of latent viral reservoirs. Recent reports showed that CD4+ T cells can also be found in the brain, reinforcing the importance of HIV reservoirs in the CNS.^25^^,^^26^ However, few studies have specifically investigated CNS reservoirs, which are uniquely challenging due to the BBB, cellular heterogeneity, and limited immune surveillance.^27^ Our study suggests that CNS may also serve as a critical reservoir for SIV, even under long-term ART suppression, aligning with observations in people with HIV-1.^5^^,^^6^ We detected broad distribution of SIV DNA across multiple brain regions, indicating that virus can disseminate widely within the brain and that proviral DNA can persist in brain cells. IPDA further identified intact genomes in select CNS sites as well as classical lymphoid reservoirs. Notably, intact proviruses were less frequent than total Pol/Vpr signals in the brain, consistent with the known predominance of defective genomes in treated infection and the triphasic decay kinetics of intact SIV genomes during ART.^28^ The presence of hypermutated barcodes in some CNS samples further suggests ongoing host-mediated editing and defective integration events in this compartment.^29^^,^^30^ During specific clinical states, including neurosymptomatic CSF escape under suppressive ART, drug-resistant variants may replicate locally and drive inflammation.^31^ Together, these observations reinforce the view that a persistent and heterogeneous CNS reservoir remains present during ART with ongoing potential neuroinflammatory consequences linked to HAND in a subset of individuals.

The barcode analysis revealed overall limited diversity in the CNS and peripheral tissues, suggesting that early founder viruses may establish long-lived reservoirs. Furthermore, CNS viruses exhibit genetic compartmentalization and clonal expansion, indicating that they are seeded independently and evolve under distinct selective pressures.^32^^,^^33^ The localized reservoirs may arise due to the limited trafficking of immune cells and cART compounds across the BBB, highlighting the importance of targeting distinct CNS niches for viral eradication.^9^ In this regard, the use of genetically barcoded virus provides an additional opportunity to compare the genetic diversity of viral reservoirs in brain versus peripheral tissues and to further evaluate the extent to which the CNS may represent a distinct viral compartment.

The use of dual gRNAs targeting Ψ and Gag achieved successful cleavage and excision of proviral DNA in multiple tissues, with no off-target editing detected in the potential sites (Figure S5), supporting the feasibility of systemic AAV delivery for CNS-targeted CRISPR-Cas9 strategies. Importantly, this approach was safe and did not provoke overt inflammation or toxicity. Our results further imply that AAV9-mediated delivery of CRISPR can induce editing of proviral DNA in the brain, primarily through indel formation and, in some instances, albeit at lower frequency, through dual cleavage at Gag and Ψ that results in excision of the intervening viral DNA from integrated host genomic DNA. In tissues from CRISPR-treated monkeys, the dual Ψ and Gag gRNAs produced not only precise excision of the targeted Ψ-Gag intervening segment but also a range of imperfect repair products, including variable breakpoint deletions and small indels at the target sites. Such heterogeneity is expected as NHEJ/MMEJ-mediated end joining pathways following two Cas9-induced double-strand breaks, and similar outcomes have been reported in paired-gRNA deletion strategies and CRISPR approaches designed to target integrated HIV DNA.^34^^,^^35^ Importantly, even when perfectly rejoined excision products are not recovered, indel-bearing junctions may still functionally inactivate proviral genomes, supporting the biological relevance of these mixed editing outcomes. At the same time, our findings do not exclude the possibility that some viral DNA detected in the brain represents genetically defective proviruses. However, the identification of precise excision products spanning the planned CRISPR target sites strongly supports authentic *in vivo* editing of integrated viral DNA within brain tissues and cells. Thus, while defective genomes may contribute to the total proviral signal, the recovery of predicted excision junctions provides compelling molecular evidence that CRISPR-mediated targeting occurred directly within CNS-associated proviral DNA.

While our results demonstrate the potential of CRISPR-Cas9 to target CNS viral reservoirs, several limitations should be addressed in future studies. Because this study included only male rhesus macaques due to cohort limitations, future translational studies should include both male and female animals to determine whether sex-associated differences influence CNS reservoir persistence, AAV9-CRISPR-Cas9 delivery, and editing outcomes. The cell lines used here were intended to validate the activity of the dual-gRNA CRISPR-Cas9 strategy and confirm targeted editing of the SIV genome. However, the observation under optimized *in vitro* electroporation or transduction conditions may not directly predict *in vivo* CNS effect, where vector delivery, cell-type accessibility, viral DNA abundance, anatomical distribution, and local tissue barriers may all influence the outcomes. Although promising, CRISPR editing did not fully eliminate intact proviral DNA, possibly due in part to limited AAV9-mediated transduction efficiency in certain brain regions.^36^ Cas9 was broadly distributed in the brain of AAV9-treated animals, with excision events observed more frequently in the basal ganglia and temporal cortex, while other brain regions such as the hippocampus showed comparatively fewer editing events, likely reflecting variable delivery kinetics of AAV9 tropism or regional differences in vector uptake.^37^ Residual intact genomes persisted in some regions of treated animals, emphasizing the need to optimize delivery and editing depth to achieve broader CNS coverage. CSF viral loads at necropsy were very low or undetectable, likely due to prolonged ART-mediated viral suppression and the limited duration of ATI (Table S4). Future studies with systematic longitudinal CSF sampling will be needed to determine whether CNS editing affects the timing and kinetics of CSF viral rebound. Moreover, latency reversal was not induced in this study, which may limit the accessibility of CRISPR-Cas9 to deeply latent proviruses.^38^ Combining gene-editing approaches with latency-reversing agents and immunotherapy strategies may improve overall clearance.^39^

The ability to target CNS reservoirs represents a critical advancement in curative HIV research, as many current strategies focus predominantly on peripheral reservoirs while the CNS remains relatively neglected.^40^^,^^41^^,^^42^ Longitudinal analyses in ART-suppressed people with HIV-1 further indicate that the proviral reservoir can remain remarkably stable over time,^43^ and studies of brain tissue demonstrate persistence of intact proviruses within the CNS compartment.^44^ This study therefore helps bridge an important translational gap toward achieving HIV remission or elimination. Our findings highlight the potential of AAV9-CRISPR-Cas9 gene editing as a therapeutic strategy for targeting SIV/HIV reservoirs within the CNS. Successful excision of proviral DNA in ART-suppressed macaques demonstrates that even difficult-to-reach sanctuary sites can be accessed by this approach. Further optimization and combination approaches may improve the likelihood of achieving a functional cure. Overall, this study provides a comprehensive CNS-wide evaluation of *in vivo* SIV excision using a CRISPR strategy in a large animal model.

## Materials and methods

### Cell culture

The 3D8 cells (obtained through the NIH HIV Reagent Program, Division of AIDS, NIAID, NIH: Simian Immunodeficiency Virus, mac316 infected CEMx174 Cells, Clone 3D8, ARP-13239, contributed by Dr. Mario Roedrer and Dr. Joseph Mattapallil) were maintained in RPMI medium supplemented with 10% fetal bovine serum (FBS) and gentamicin (10 μg/mL; Sigma-Aldrich, St. Louis, MO, USA). The macaque microglia M1A cells were kindly provided by Dr. Yoelvis Garcia-Mesa and Dr. Jonathan Karn at Case Western Reserve University.^45^ The cells were cultured in DMEM/F12 medium supplemented with glutamine, 5% FBS, 1× N2, 1× non-essential amino acids (NEAA), 10 mM HEPES, and Plasmocin (2.5 μg/mL) in flasks coated with 0.5% gelatin solution at 37°C for at least 30 min, followed by aspiration before cell plating.

### Animal study design

Ten adult male rhesus macaques (Macaca mulatta) were enrolled in this study. All animals were intravenously inoculated with a barcoded SIVmac239M2 (10,000 IU/mL) (a generous gift from Brandon Keele, barcode sequences shown in Table S5) and after two weeks maintained on a daily subcutaneous ART regimen comprising tenofovir (5.1 mg/kg), emtricitabine (30 mg/kg), and dolutegravir (2.5 mg/kg). ART was administered for a minimum of 20 months to ensure sustained viral suppression before CRISPR treatment. All procedures were approved by the Institutional Animal Care and Use Committee (IACUC) and conducted under protocols at an AAALAC International-accredited facility.

### CRISPR-Cas9 vector design

Dual guide RNAs (gRNAs) targeting the conserved Ψ (packaging signal) and Gag (structural polyprotein) regions of the SIV genome were designed based on bioinformatics predictions using *Benchling* and *CRISPOR* tools to ensure high on-target efficiency and minimal off-target activity (Table S5). The two gRNAs were cloned into an AAV9 vector containing *Staphylococcus aureus* (SaCas9) under the control of a EF1α promoter (Figure S3).

### RNP-CRISPR electroporation

Guide RNAs were coupled individually with SaCas9 by adding 2.2 μL of sNLS-SaCas9-sNLS Nuclease (10 μg/mL = 80 μM, Aldevron, #9218-0.25MG) to 6 μL of synthetic gRNA (60 μM, CRISPRevolution Custom RNA modified, Synthego) in 11.8 μL of electroporation Buffer R (Neon, Invitrogen). The molar ratio when incubating was 1:2 (9 μM:18 μM) SaCas9: gRNAs. The mixtures were incubated individually for 10 min at room temperature (RT), then combined and added to 2.5 × 10^6^ of target cells in 200 μL of Buffer R. Cells were then mixed gently and electroporated using Neon Electroporation Device set to 1400 V, 1 × 30 ms impulse with 100 μL Neon tip. Cells were immediately plated in 4 mL of pre-warmed growth medium.

### AAV9-CRISPR administration

Animals were randomly assigned into two groups: treatment (*n* = 5) and control (*n* = 5). The treatment group received a slow intravenous infusion of AAV9-CRISPR vectors containing dual gRNAs (10^13^ genome copies/kg), while control animals received AAV9-Cas9 vectors lacking gRNAs. Following systemic AAV9-SaCas9 administration, ART was discontinued one week later. Animals were monitored longitudinally for plasma viral rebound, and necropsy was performed at the onset of detectable rebound. Because rebound occurred at different times among animals, necropsy was conducted between 15 and 34 days after ART withdrawal. To minimize potential confounding effects associated with variable necropsy timing, all delivery and editing analyses were normalized to tissue input or cell equivalents, where applicable, and quantified using standardized molecular assays performed under identical experimental conditions.

### Tissue collection and DNA extraction

Upon confirmation of viral rebound, animals were euthanized and perfused with phosphate-buffered saline (PBS). Tissues including spleen, lymph nodes, liver and peripheral blood mononuclear cells (PBMCs) were harvested. A total of 19 nervous system tissues were collected from each animal, including 15 CNS regions (prefrontal cortex, frontal cortex, basal ganglia, caudate nuclei, parietal cortex, hippocampus, temporal cortex, occipital cortex, hypothalamus, substantia nigra, choroid plexus, cerebellum, brain stem, thoracic and lumbar spinal cord) and four peripheral nervous system (PNS) tissues (sciatic nerve, trigeminal ganglia, thoracic and lumbar dorsal root ganglia). Genomic DNA was extracted from tissues using the NucleoSpin Tissue kits (Macherey-Nagel, Germany) according to the manufacturer’s protocol and quantified with the Nanodrop spectrophotometry.

### PCR, droplet digital PCR, and intact proviral DNA assay

#### Exa-band PCR

To detect excision events, amplicons spanning the Ψ and Gag cut sites were generated by nested PCR using flanking primers listed in Table S6. PCR was performed with the Terra PCR kit (Takara, Japan) using an initial input of 100 ng template DNA, with cycling conditions, primer concentrations, reagent volumes, and other reaction parameters set according to the manufacturer’s instructions. Products were visualized by agarose gel electrophoresis and confirmed using Sanger sequencing (Azenta, South Plainfield, NJ, USA).

#### Near full-length PCR

SIV proviral DNA from 3D8 cells was amplified using a two-round hemi-nested long-range PCR strategy designed to generate NFL amplicons tagged with unique molecular indices (UMIs) for downstream nanopore sequencing. For round 1, reactions contained 100 ng template DNA in a 50 μL reaction consisting of 25 μL Platinum SuperFi II Master Mix, 18 μL nuclease-free water, 2 μL combined SIV-NFL-For and SIV-NFL-Rev primers (Table S6) at 250 nM each, and 5 μL DNA template. Round 1 cycling was performed with an initial denaturation at 98°C for 30 s, followed by 30 cycles of 98°C for 10 s, 60°C for 10 s, and 72°C for 210 s, with a final extension at 72°C for 300 s and hold at 4°C. PCR products were purified using Nanopore Ampure XP beads at a 0.8× bead-to-sample ratio and eluted in nuclease-free water. Round 2 PCR used 5 μL purified round 1 product in a 50 μL reaction containing 25 μL Platinum SuperFi II Master Mix, 19.8 μL nuclease-free water, 0.1 μL R2 forward ONT primer and 0.1 μL R2 reverse ONT primer (Table S6), each at 250 nM, and was amplified using the same thermocycling program. The round 1 primers targeted SIV proviral regions and introduced 16 basepair unique UMIs and synthetic priming sites, while the round 2 primers targeted these synthetic sites and added Oxford Nanopore-compatible overhangs.

All ddPCR was conducted using the Bio-Rad QX200 system (Bio-Rad Laboratories, Hercules, CA, USA) to detect SIV Pol, Vpr, SaCas9, gRNAs sequences, and TERT gene as a reference in tissue DNA samples. It was also performed to quantify the relative abundance of SIV Gag and Env genes, providing an indirect measurement of excision efficiency by the loss of Gag/Env signal ratio. The primers and probes for each region are listed in Table S6. Intact proviral DNA was detected using a validated SIV- specific IPDA protocol employing dual-target probes against Pol and Env genes to distinguish intact from defective or hypermutated viral genomes as previously described.^28^

### Nanopore sequence analysis

Products from either the Exa-band PCRs or SIV-NFL were sequenced by nanopore long read sequencing using R14 chemistry with the Native Barcoding Kit (SQK-NBD114.24) and the Ligation Sequencing DNA Kit (SQK-LSK114) according to the manufacturer’s instructions. Products were collected into batches of 6 and sequenced for 12 h. Raw Pod5 files were duplex basecalled using dorado (v0.9.1) with the model duplex model dna_r10.4.1_e8.2_5khz_stereo@v1.3 and simplex model dna_r10.4.1_e8.2_400bps_sup@v5.0.0.

The SIV-NFL samples were subjected UMI consensus merging using a modified version of the Nextflow umi-pipeline-nf.^46^ Three algorithmic refinements were introduced to better accommodate the structural diversity of lentiviral amplicons sequenced on Oxford Nanopore platforms. First, the read-length filter used to exclude spurious concatemers and truncated molecules was extended with a deletion-tolerant mode. In the original implementation, a read was classified as too short whenever its aligned query span fell below a fixed fraction (minimal_overlap) of the target amplicon length, which inadvertently discarded genuine reads carrying large internal deletions—a hallmark of defective genomes. Under the new mode, the short-read criterion was instead based on the breadth of reference coverage across the BED-defined amplicon window, so that a read harboring a large internal deletion but still spanning the full target locus was retained. Correspondingly, the long-read (concatemer) cutoff in this mode evaluated the aligned query span rather than the total read length, preventing intact long-insert reads from being mis-classified as concatemers. An additional parameter allowed the upper length threshold to be set explicitly when the amplicon geometry departs substantially from the default formula. Second, a length-stratified subclustering step was added downstream of UMI-tag-based grouping. After reads sharing a UMI sequence were consolidated into subclusters by pairwise edit distance, each subcluster was further partitioned according to amplicon length using single-linkage clustering, with membership controlled by either an absolute base-pair tolerance or a percentage difference. This refinement addresses the scenario in which intact and internally deleted viral genomes co-occur in the same UMI family—whether by UMI collision or by sharing edit-distance-similar tags—which would otherwise yield a chimeric consensus sequence that corresponds to neither biological molecule. By resolving these length-distinct molecules before polishing, each class was independently corrected and reported. The modified version is available at https://github.com/DamLabResources/umi-pipeline-nf-HIV, commit 19f7d47 was used for this study.

The raw reads in the case of Exa-band PCR or in the case of SIV-NFL, the UMI consensus reads were subjected to a set of custom python scripts to characterize the structural outcomes of CRISPR editing in individual lentiviral genomes, each nanopore sequencing read was processed through a multi-stage classification pipeline. First, raw reads were aligned to the proviral reference sequence using minimap2 (v2.28) with an Oxford Nanopore-optimized preset, producing a set of primary and supplementary alignment segments per read. Because lentiviral long terminal repeats (LTRs) are nearly identical at both ends of the provirus, an automated self-alignment procedure tiles the reference with overlapping seed windows to precisely define the 5′ and 3′ LTR boundaries, and any supplementary alignments incorrectly placed at the wrong LTR copy are detected and re-aligned locally to the correct copy. Nanopore duplex reads—in which two complementary strands are sequenced in tandem and produce a single concatenated signal—were detected by their characteristic reference topology and split into their two constituent strand-level events so they do not appear as spurious structural rearrangements. Alignment segments mapping outside the expected amplicon window were then discarded. With the normalized alignment in hand, each read’s CIGAR strings and inter-segment junctions were parsed to enumerate all structural events: small insertions and deletions, large intra-molecular deletions, inter-contig translocations, inversions, and circular reference wraps. Each event was annotated with its nearest CRISPR guide RNA (gRNA) cut site, and small indels falling within a defined window around each cut are recorded as target-site edits. Based on the combination of events present, every read was assigned one of seven mutually exclusive summary classes: *clean* (no edits or rearrangements), *indel at one or both sites* (small insertions or deletions at one or more cut sites but no large structural changes), *intended deletion* (a deletion spanning and bridging two distinct guide target sites, consistent with excision of the intervening sequence), *hemi-sided deletion* (a large structural rearrangement whose breakpoints map near a cut site), *unintended deletion* (a structural rearrangement whose breakpoints are not associated with any guide target), *mixed* (a combination of on- and off-target events in the same molecule), or no_alignment (the read failed to map). Each read was additionally flagged for whether it spans the complete expected genomic interval—a proxy for molecule integrity—and for its inferred topology (linear, circular, or multi-molecule concatemer).

### Off-target analysis by rhAMP-Seq

Potential off-target sites for the Ψ_112 and Gag_468 guide pair were nominated using Excision BioTherapeutics’ COTANA bioinformatic pipeline, which is based on Cas-OFFinder and incorporates a customized SaCas9 position-weight matrix. The rhesus macaque genome was searched using the SaCas9 PAM sequence 5′-NNGRRN-3′, allowing up to five mismatches and one bulge. Candidate sites were filtered and ranked based on mismatch number, bulge presence, PAM orientation, and predicted positional tolerance. A total of 49 nominated off-target sites were selected for analysis (Table S7).

rhAMP-Seq primer sets were designed using the IDT rhAMP primer design tool. Primers targeting the nominated off-target sites were pooled into a single primer set for the Ψ_112 and Gag_468 guide pair. Two rounds of PCR were performed according to the manufacturer’s instructions, and PCR products from different samples were pooled at equal molar ratios before 2 × 150 bp paired-end sequencing on an Illumina NextSeq platform.

Sequencing data were analyzed using Excision BioTherapeutics’ targeted amplicon sequencing pipeline. Reads were processed using FASTP for adapter, quality, and poly-G trimming. Insertions or deletions within ±5 bp of the predicted SaCas9 cut sites were quantified at each nominated off-target site. Structural variants were also evaluated. Statistical comparisons between treated and control samples were performed using a beta-binomial model, and power analysis was conducted to estimate the detection sensitivity at each site.

### Amplicon barcode sequencing and clonal diversity analysis

Animals were infected with SIV tagged with a 34-nt cassette containing ten base pair (bp) random barcode sequences between the Vpx and Vpr genes. Viral barcode high-throughput sequencing was performed on the DNA prepared from the collected tissues. Regions flanking the barcoded sequences were amplified using nested PCR. Sequencing adapters were subsequently added in a third round of PCR using Illumina indexing primers (Table S6). Equal amounts of the barcoded PCR products were pooled and submitted to Azenta/Genewiz for 2 × 250 bp paired end deep sequencing.

Reads were analyzed using SIV barcode pipeline (adapted from https://github.com/DamLabResources/outerspace). First, demultiplexed R1 and R2 reads were merged using BBMerge. Reads that could not be merged were retained as single end reads. The merged FASTQ files were then converted to BAM format. For each merged or single end read, the 34-nt barcode was extracted by identifying 27 bp upstream and 26 bp downstream flanking sequences using a Python regular expression, allowing up to four mismatches (including a maximum of two insertions and deletions [indels]). Extracted barcodes were error-corrected and collapsed using UMI-tools (https://pmc.ncbi.nlm.nih.gov/articles/PMC5340976/), permitting up to three differences.^47^ The resulting corrected barcodes were used to calculate barcode diversity (richness = number of unique barcode sequences post-correction) and for subsequent downstream analyses. To normalize richness, the values are adjusted based on the total number of reads and scaled to a standardized total of 1,000 reads.

### Statistical analysis

Quantitative results are presented as mean ± standard deviation (SD). Group comparisons were performed using unpaired two-tailed student’s t-tests or one-way ANOVA with Tukey’s multiple comparison test, as appropriate. Statistical significance was defined as *p* < 0.05. Graphs were generated in GraphPad Prism version 10.0.

## Data and code availability

The data supporting the findings of this study are available within the article and its supplemental materials. Additional data are available from the corresponding author upon reasonable request.

## Acknowledgments

The authors wish to thank past and present members of the Center for Neurovirology and Gene Editing for their support and sharing of reagents. We thank Dr. Maurizio Caocci from the Burdo laboratory for the preparation and distribution of brain tissues provided by our colleagues at BioQual, Inc.; Dr. Brandon Keele for providing the barcoded SIV; Dr. Yoelvis Garcia-Mesa and Dr. Jonathan Karn for kindly providing the M1A cells; and Merck and ViiV Healthcare for the antiretrovirals. We also thank the Duke Human Vaccine Institute for CSF viral loads (NIAID DAIDS Virology Core Laboratory; contract # 75N93025C0002), and the BioQual staff for animal care and experimental administration. We appreciate Cynthia Papaleo for the assembly of data and preparation of this manuscript. This research was supported by the CRISPR for Cure Martin Delaney Collaboratory for HIV cure UM1 AI164568 and co-funded by 10.13039/100000060NIAID, 10.13039/100000025NIMH, 10.13039/100000026NIDA, 10.13039/100000065NINDS, 10.13039/100000062NIDDK, and 10.13039/100000050NHLBI. This study utilized services offered by core facilities of the Comprehensive NeuroHIV Center (10.13039/100018999CNHC 10.13039/100000025NIMH grant number P30MH092177).

## Author contributions

Conceived the idea and supervised editing strategy: K.K.; construction of gRNAs and other reagents: C.C.; designed the editing experiments: K.K.; design of viral infection studies, communication with animal facility, tissue preparation and distribution: T.H.B.; performed the experiments: H.L., S.L., Z.S., W.H., and Y.H.; performed animal experiments: M.L. and C.A.L. (Bioqual facility); interpret the data: K.K., T.H.B., J.G., W.D., M.R.N., and B.W.; preparation and comments of manuscript: H.L., K.K., I.K.S., J.G., W.D., M.R.N., and B.W.; preparation of data and figures: A.R., H.L., I.K.S., W.D., and M.R.N. SIV sequencing and analysis: M.E.C., J.M.L., W.D., M.R.N., and B.W.

## Declaration of interests

The authors H.L., S.L., C.C., Z.S., A.R., Y.H., I.K.S., M.E.C., J.M.L., W.D., M.R.N., B.W., M.L., and C.A.L. declare no conflict of interest. W.H. and J.G. were employees of Excision BioTherapeutics. K.K. is named inventor on patents that cover the viral gene editing technology that is the subject of this article.

## Supplemental information

Document S1. Figures S1–S5 and Tables S1–S7

Document S2. Article plus supplemental information
