# Supplementary material for: In vivo genome editing of central nervous system SIV reservoirs in ART-suppressed rhesus macaques
Source: Mol Ther Nucleic Acids. 2026 Aug 14;37(3):103063. doi: 10.1016/j.omtn.2026.103063 (PMC13560538; doi:10.1016/j.omtn.2026.103063)
Supplement: Supplementary file 1 [file mmc1.pdf]

OMTN, Volume *37*

Supplemental information

In vivo genome editing of central nervous system SIV reservoirs in ART-suppressed rhesus macaques

Hong Liu, Shuren Liao, Chen Chen, Wenwen Huo, Zahra Safaei, Angela Rocchi, Yuru Huang, Ilker K. Sariyer, Mackenzie E. Collins, Jill M. Lawrence, Will Dampier, Michael R. Nonnemacher, Brian Wigdahl, Tricia H. Burdo, Jennifer Gordon, Mark Lewis, Cheri A. Lee, and Kamel Khalili

Supplemental Figures

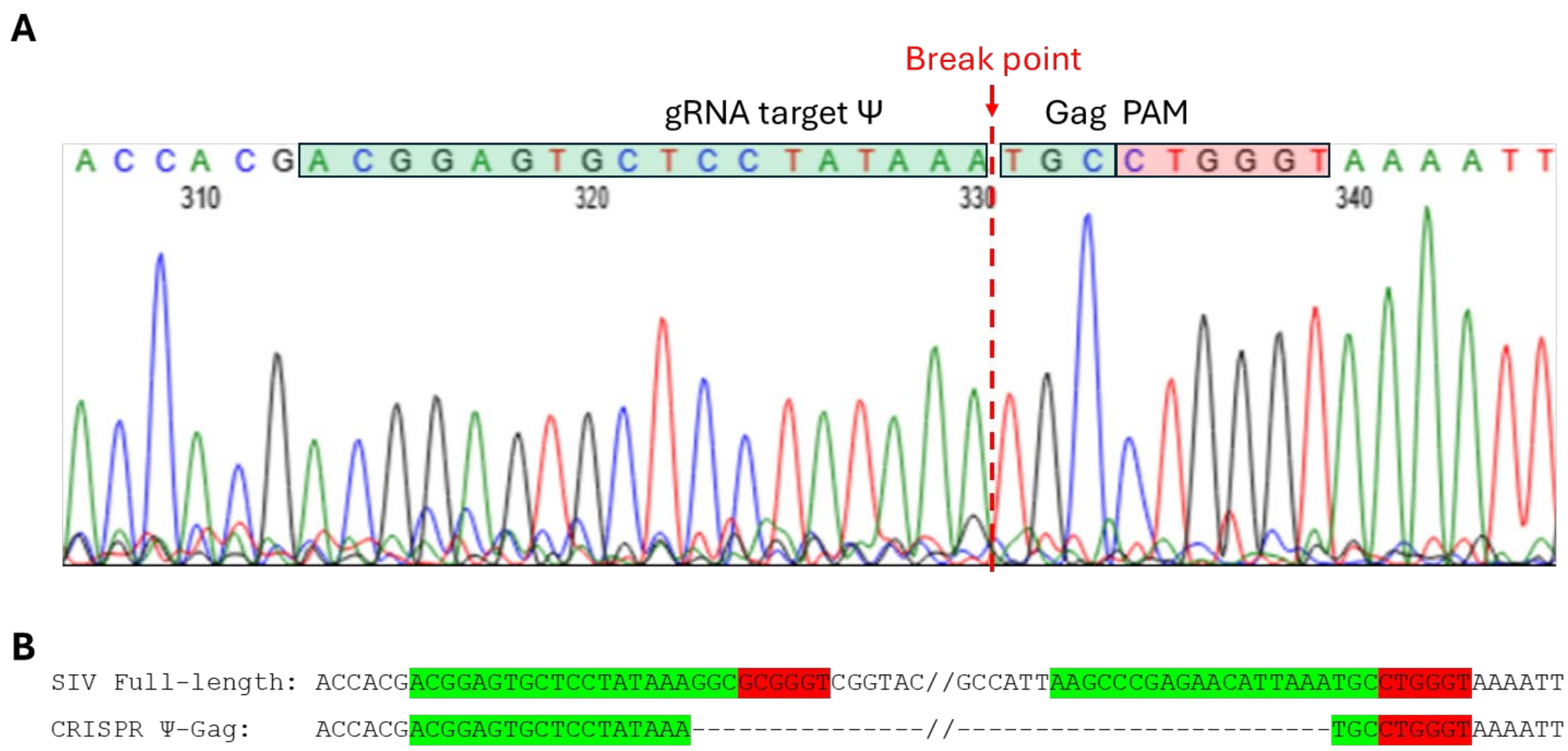

Figure S1. Sanger sequencing analysis of CRISPR-mediated excision of the SIV Ψ–Gag region in 3D8 cells. (A) Representative Sanger sequencing trace of the truncated amplicon generated by CRISPR cleavage and end joining within the SIV Ψ–Gag region in 3D8 cells. (B) Sequence alignment of the corresponding edited amplicons. The gRNA target sites are highlighted in green, and PAM sequences are shown in red.

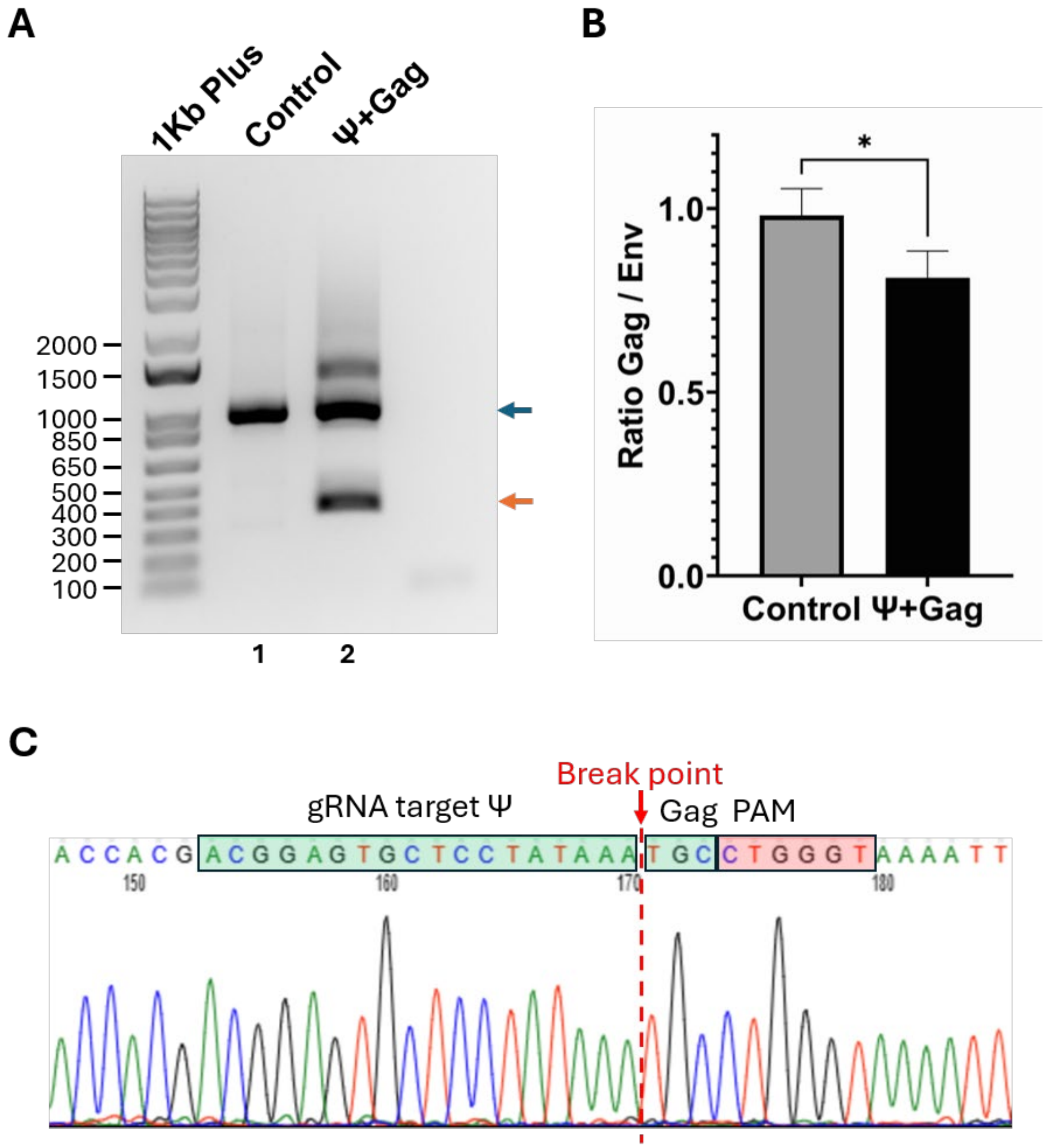

Figure S2. CRISPR-mediated excision of the Ψ–Gag region in SIVmac239-infected macaque microglial M1A cells. (A) PCR analysis of the Ψ–Gag region using genomic DNA isolated from SIVmac239-infected M1A macaque microglial cells electroporated with Cas9 RNPs containing both Ψ and Gag targeting gRNAs. Dual-gRNA editing produced a smaller amplicon corresponding to excision of the 590-bp Ψ–Gag segment. The orange arrow denotes the excision-specific band, whereas the blue arrow denotes the full-length band. (B) Quantification of excision efficiency by ddPCR. The reduced Gag-to-env signal ratio reflects deletion of the Gag segment in SIV-infected M1A cells. (C) Representative Sanger sequencing trace of the truncated amplicon indicated by the orange arrow in panel A, confirming CRISPR-mediated cleavage and end joining within the SIV Ψ–Gag region in M1A cells.

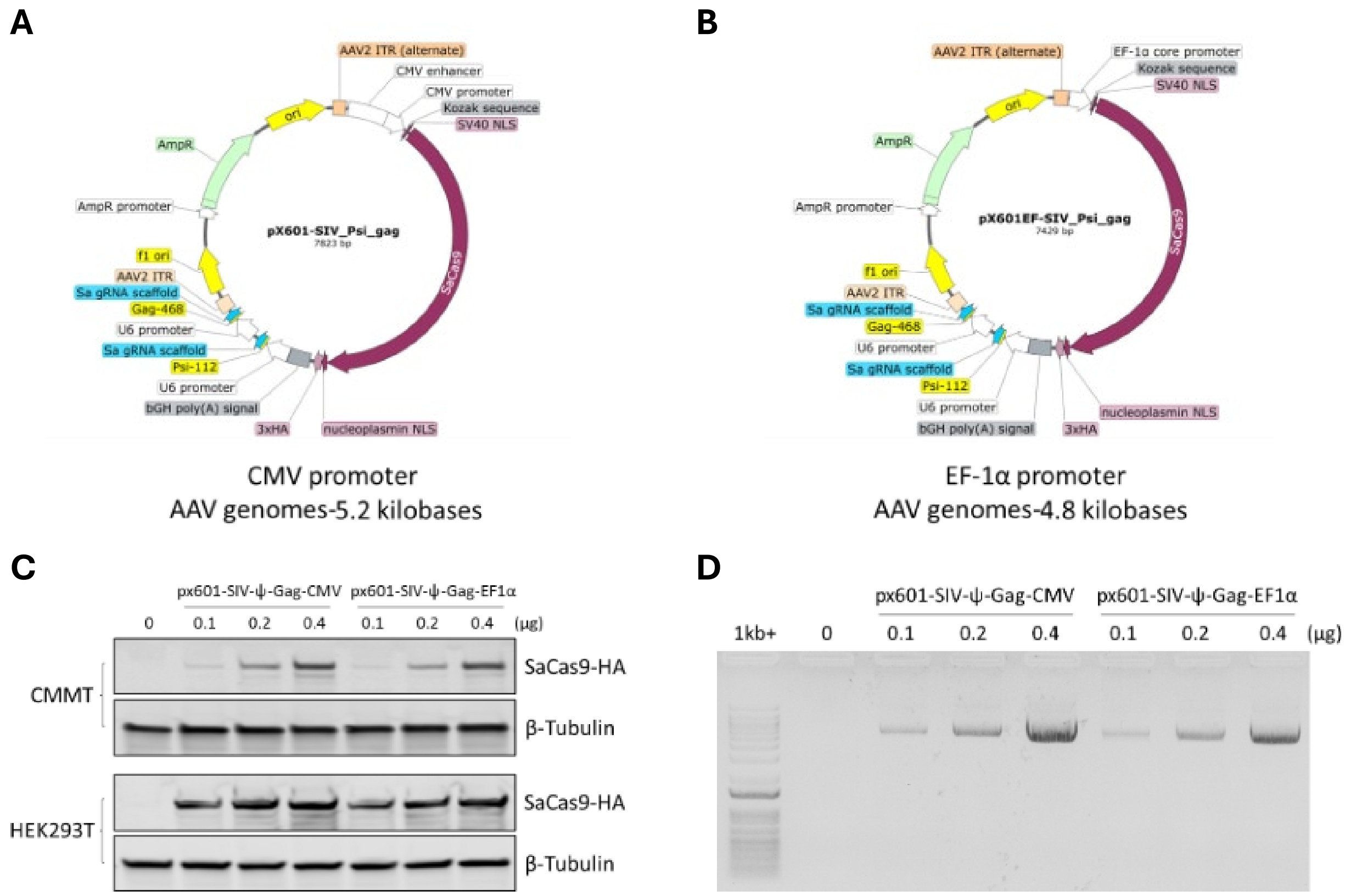

Figure S3. The EF1α core promoter maintains comparable SaCas9 expression while reducing vector size to improve AAV packaging. (A) Vector map of the pX601-SaCas9 construct containing dual gRNAs targeting SIV Ψ and Gag. The CMV promoter drives SaCas9 expression (red), and two gRNA cassettes (yellow target sequence + blue scaffold) encode guides directed against the SIV Ψ packaging signal and the Gag coding region. (B) Vector map of pX601-EF1α-SaCas9-SIV Ψ-Gag. In this construct, the CMV promoter was replaced with the EF1α core promoter to reduce vector genome size and improve compatibility with AAV9 packaging constraints. (C) Increasing amounts of px601-SIV-Ψ-Gag-CMV or px601-SIV-Ψ-Gag-EF1α plasmids were transfected into CMMT monkey cells or HEK293T human cells. SaCas9 expression levels were assessed by western blot analysis 72 hours post-transfection, with β-tubulin used as a loading control. (D) Equal amounts of plasmid DNA used in the transfection experiments were analyzed by agarose gel electrophoresis to confirm comparable DNA input between px601-SIV-Ψ-Gag-CMV and px601-SIV-Ψ-Gag-EF1α plasmids.

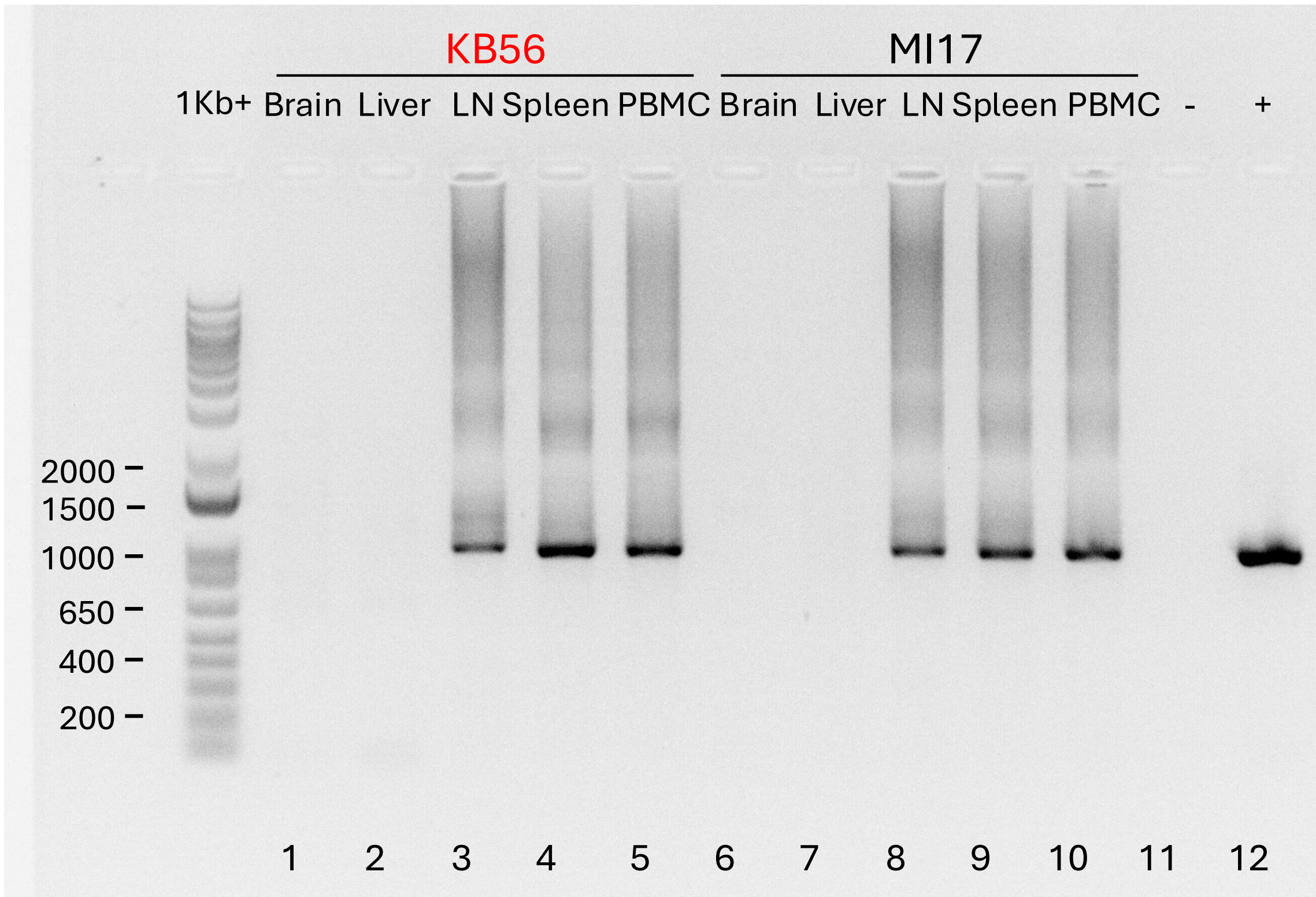

Figure S4. Nested PCR analysis of the SIV Pol region in tissues from ART-suppressed rhesus macaques. Agarose gel electrophoresis of nested PCR amplicons targeting the SIV Pol region showed products of similar size across tissues from both CRISPR-treated (KB56) and control (MI17) animals, with no truncated bands detected. Negative and positive controls consisted of water input and 3D8 cell genomic DNA, respectively.

A

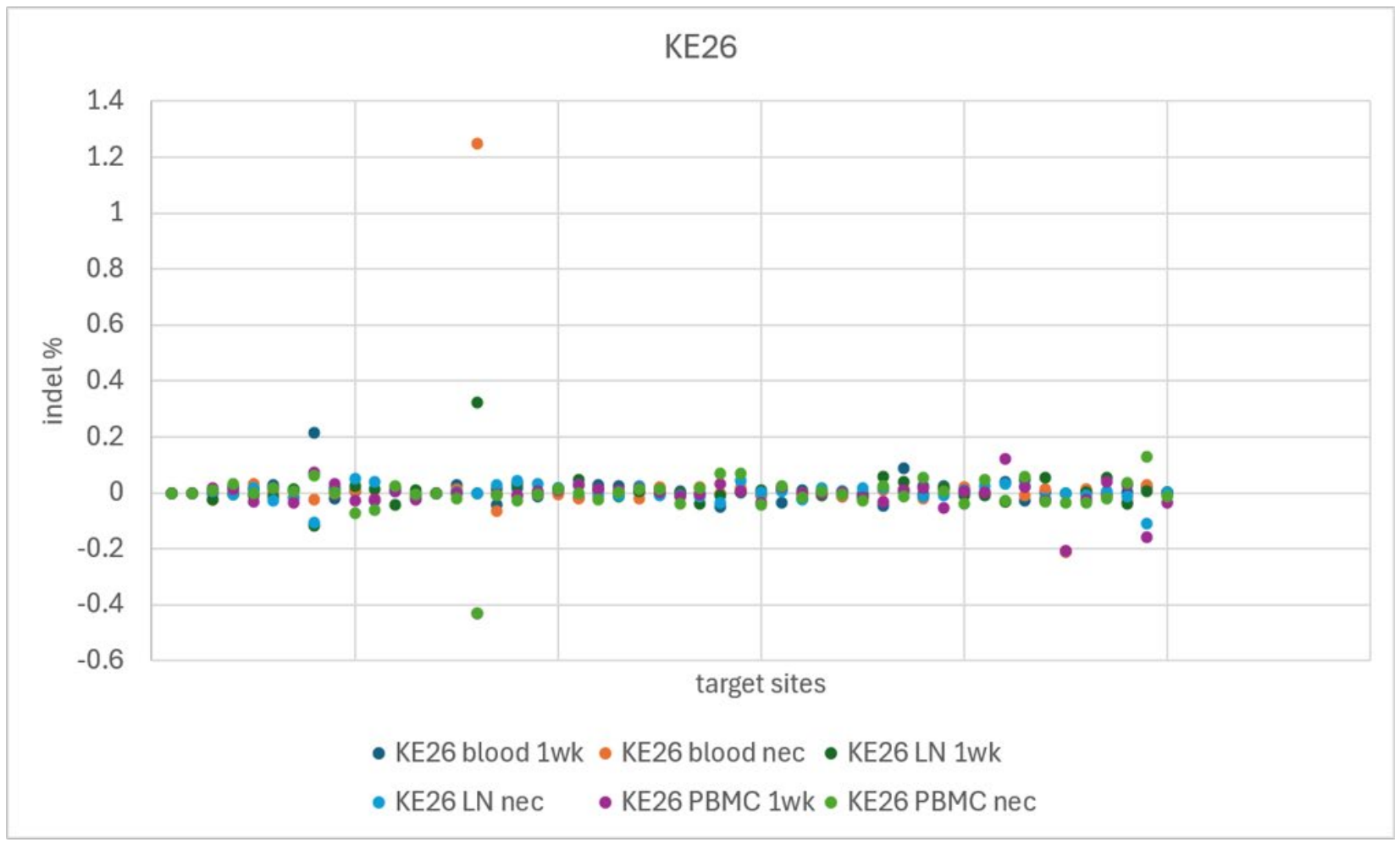

B

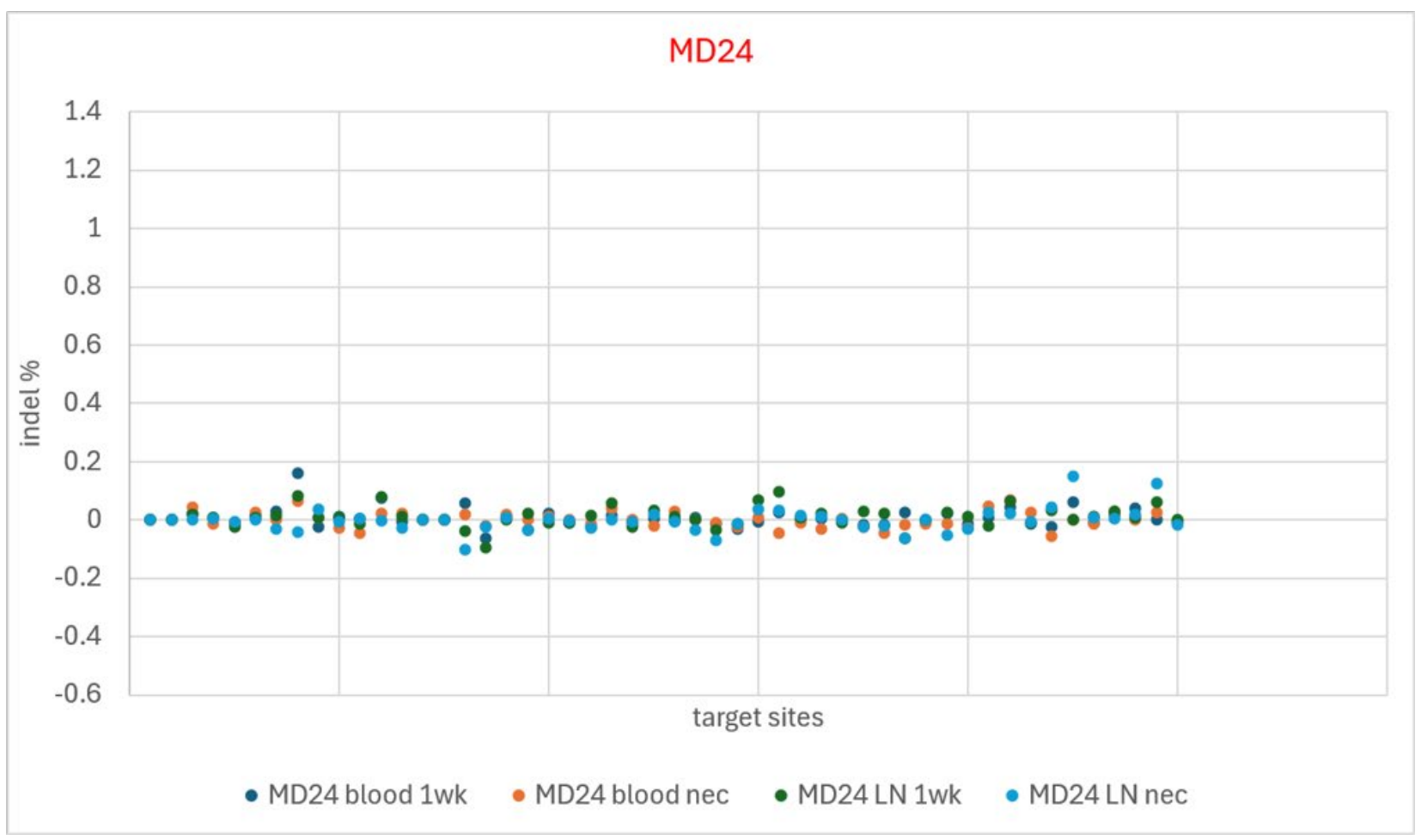

Figure S5. Delta indel frequencies at nominated off-target sites across regional tissue samples from two monkeys. For each nominated off-target site, the change in indel frequency (delta indel frequency) was calculated as the percentage of indel-containing reads in the post-CRISPR sample minus that in the matched pre-CRISPR baseline sample. Each dot represents one nominated off-target site in one tissue sample. Panel A shows Monkey KE26 (control), and panel B shows Monkey MD24 (treated). Distinct colors represent individual samples within each monkey. Off-target sites are arranged along the x-axis in the same order as listed in Table S7. Negative delta indel frequencies likely reflect technical variation resulting from unequal sequencing depth between baseline and post-CRISPR samples. At sites with relatively low baseline read depth, a single indel-containing read can disproportionately inflate the estimated background indel frequency, resulting in an artificial negative value after subtraction. These negative values should therefore be interpreted as baseline noise rather than evidence of reduced indel formation. Sequencing depth for all sites and samples is provided in Table S1. “1 wk” denotes samples collected 1 week after AAV9 treatment, and “nec” denotes samples collected at necropsy.

Supplemental Tables

Table S1. Aligned read depth at nominated off-target sites across regional tissue samples from two monkeys.

	KE26 blood			MD24 blood			KE26 LN cells			MD24 LN cells			KE26 PBMC		
Target Id	pre-	1 wk	Necro	pre-	1 wk	Necro	pre-	1 wk	Necro	pre-	1 wk	Necro	pre-	1 wk	Necro
Ψ112	0	0	0	0	0	0	1	0	2	0	1	2	1	0	0
Gag468	4	2	2	12	0	0	3	0	15	1	5	27	8	2	0
Gag468.188	32910	38839	33367	44180	47877	34770	37740	42495	26750	31947	45884	33430	40762	41429	13816
Gag468.1864	32480	39241	34483	44968	48201	38739	38923	44085	29428	37013	44976	39333	42942	41081	12766
Gag468.2361	25013	31394	25134	34899	36532	26772	27937	31884	18583	21949	32955	25627	29919	30894	8942
Gag468.2797	23138	26902	23081	30181	31085	23752	24199	27887	16843	18783	27785	20132	25001	26909	8291
Gag468.3388	22964	27920	23079	30918	32591	24176	24689	27430	16573	20349	27761	20911	26182	27441	8328
Gag468.3487	3291	3848	4042	2990	3852	3614	5672	6241	5703	5356	5140	5529	5189	5319	1695
Gag468.3875	32477	40040	34186	45423	47898	36981	40057	45222	27912	31205	46372	35957	42350	44344	13446
Gag468.4060	10975	13168	10778	14270	16071	10609	12033	13935	8356	9813	13767	11315	12620	14414	3972
Gag468.4398	12167	14301	12658	17194	17387	13553	13392	15714	9963	11281	15131	11643	15068	15538	5023
Gag468.7037	8175	10580	9061	12098	13140	10299	8852	10465	6964	8432	9735	9065	10244	11269	3119
Gag468.7156	15338	19174	16148	15020	15349	11819	17853	19698	14059	10713	14042	11663	19272	20924	6568
Gag468.7383	0	2	0	0	1	0	1	0	2	2	0	2	2	1	2
Gag468.8142	22697	27516	24880	195	147	156	25233	30364	19833	212	206	158	28350	29266	9935
Gag468.8966	199	250	160	1457	1546	1284	243	308	225	969	1519	1153	233	254	90
Gag468.9400	5826	8008	6605	8591	10266	6835	7522	7578	4951	5530	7515	6242	6905	7984	2501
Gag468.9813	16718	21875	17782	21403	22418	17700	18660	20403	14658	14549	16324	14636	21385	22236	6941
Gag468.10211	20363	25629	20097	12475	12914	8599	26206	29639	17482	8486	14372	10434	26944	29091	8644
Gag468.11495	37801	45480	37693	52462	55633	38773	46230	52592	33749	37073	54860	41880	47627	50431	16133
Gag468.11808	36377	42897	37607	49271	51170	39474	41175	46069	29671	34530	48418	35405	43680	45816	15633
Gag468.12030	18032	21399	18375	23826	24276	19445	21729	23385	15451	18621	22618	19750	21350	21189	6567
Gag468.12674	21219	26068	22085	26457	27957	20760	23567	26478	17819	17482	21229	19627	25871	26773	8571
Gag468.13583	22766	27416	24302	32130	34649	25676	24525	26290	18494	21914	26004	24435	28738	28355	8431
Gag468.14061	34426	42003	36746	46823	49186	37368	38925	44647	27752	31300	44746	33968	41461	42910	13924
Gag468.14698	27812	34015	29214	38522	41570	30601	30183	36124	22215	25423	36450	26906	33823	34685	11077
Gag468.14829	12028	14614	12708	16210	17168	13475	15682	17999	10592	14834	18779	15119	15561	15193	4275
Ψ112.461	10277	13336	10556	15411	17239	12705	12950	14327	8420	9849	15027	11865	13254	15444	4411
Ψ112.1278	24548	30467	27027	36135	37727	30569	27816	30832	20392	26320	32962	26740	31166	33118	10022
Ψ112.1523	21108	26375	21293	19379	21186	15198	23541	27236	17456	12210	17276	13878	25489	27063	8737
Ψ112.2484	11153	14017	11303	19382	21205	14553	13551	15462	9865	13065	19219	14250	13881	15530	5234
Ψ112.3154	12456	15293	13185	16743	19507	15107	14182	16070	10747	12065	16993	12436	15408	17387	5507
Ψ112.3637	30668	38238	32868	44833	46716	36214	33763	38878	25872	31959	37630	32808	38276	40368	12542
Ψ112.3900	16332	20951	17270	23896	26372	19119	18296	22039	14078	17590	22507	17815	20748	23318	7459
Ψ112.4440	35011	42636	37627	47104	49763	39063	41237	47524	30486	34097	48758	36020	42206	46170	16002
Ψ112.4469	32911	39176	33783	44187	48623	34805	36779	40571	25138	30330	39755	32537	40016	39528	12331
Ψ112.4645	7123	8324	7173	9456	10106	7820	11042	12181	8857	10699	13948	10379	11417	11159	3931
Ψ112.5896	37024	43677	37808	48447	50211	39922	39139	44391	27679	31975	43827	36048	44072	42769	13015
Ψ112.6825	13797	17324	14355	20283	21964	15764	15932	18985	11147	14171	20275	14599	17963	19737	5573
Ψ112.6971	31170	38240	33173	42768	44208	33682	37032	42175	25794	29849	42698	31926	39725	41406	12876
Ψ112.7215	12297	15685	12244	19316	21222	14201	14850	16878	11064	12620	18029	14379	15802	17303	5132
Ψ112.7741	3229	3816	3239	4214	4455	3224	3640	4177	2247	2466	3818	3098	3539	3996	1153
Ψ112.8341	37173	45199	35595	53001	56886	40484	37378	42681	28123	33755	43291	38673	45753	43149	12591
Ψ112.8946	11696	14316	12275	16581	17789	13593	13600	15432	10330	10776	15547	12426	14680	14792	5123
Ψ112.9260	1420	1450	1488	1642	1610	1590	1915	2264	1905	2774	2403	2009	1946	1516	584
Ψ112.9911	34856	41623	35725	48406	49925	38762	39558	45440	29714	34763	46791	35851	43655	45894	15299
Ψ112.10613	14275	16410	14123	17849	19927	15627	14939	16202	10298	12073	16686	12774	15276	16445	4983
Ψ112.10754	11215	13983	13297	16256	18192	15757	15227	17161	11310	14459	19560	14985	15872	16603	5410
Ψ112.10978	10179	13020	9748	14661	15921	9862	11761	14032	7829	9598	14459	11168	14587	14173	3256
Ψ112.11009	19083	23876	19903	26534	30260	22499	21484	24871	16857	18993	24084	19908	23576	26296	8838
ACTB	2291	2537	2904	2984	2761	3267	5138	5663	4870	7806	7463	6440	4303	3185	1156
GAPDH	4940	5502	5553	6513	6369	6494	8142	8621	6264	8874	10260	8877	7103	6527	2056

Table S2. Summarizing the positive ddPCR signaling for SIV Pol DNA in different brain regions from the 10 animals.

SIV POL detected by ddPCR	KE26	MD24	JM96	KK68	JV38	MJ21	MC43	MN45	KB56	MI17
Prefrontal Cortex	X	X	X	X						X
Frontal Cortex	X									X
Basal Ganglia										X
Caudate Nuclei	X						X			
Parietal Cortex		X							X	
Hippocampus			X				X	X		
Temporal Cortex						X		X		
Occipital Cortex			X						X	X
Hypothalamus	X					X				
Substantia Nigra			X					X		X
Choroid Plexus			X	X	X	X	X			X
Cerebellum					X					X
Brain Stem								X	X	

Table S3. Tissue distribution of intact proviral DNA (copies per 150 ng gDNA) in 10 rhesus macaques.

SIV IPDA	KE26	MD24	JM96	KK68	JV38	MJ21	MC43	MN45	KB56	MI17
Liver	0.00	0.00	0.00	0.00	1.50	0.00	6.48	0.00	0.00	0.00
Submandibular LN	40.11	26.27	40.14	34.73	11.15	3.02	10.54	1.60	11.20	6.00
Spleen cells	63.67	826.75	9.63	3.03	65.31	10.11	4.27	2.57	1.37	4.07
PBMC	5.28	0.00	NA	1.55	4.80	3.32	0.00	4.38	3.34	124.53
Prefrontal Cortex	0.00	0.00	0.00	0.00	8.00	0.00	0.00	0.00	0.00	2.76
Frontal Cortex	0.00	0.00	0.00	0.00	1.80	0.00	0.00	0.00	0.00	0.00
Basal Ganglia	0.00	0.00	1.40	0.00	0.00	0.00	0.00	0.00	0.00	0.00
Caudate Nuclei	0.00	0.00	0.00	0.00	0.00	0.00	0.00	0.00	0.00	0.00
Parietal Cortex	0.00	0.00	0.00	0.00	0.00	0.00	0.00	0.00	0.00	0.00
Hippocampus	0.00	0.00	0.00	0.00	5.85	0.00	0.00	2.60	0.00	0.00
Temporal Cortex	0.00	0.00	0.00	0.00	2.00	1.20	0.00	0.00	0.00	0.00
Occipital Cortex	0.00	0.00	0.00	9.61	0.00	0.00	0.00	0.00	0.00	1.28
Hypothalamus	1.20	0.00	0.00	0.00	0.00	0.00	0.00	1.20	0.00	0.00
Substantia Nigra	0.00	1.40	0.00	0.00	0.00	0.00	0.00	0.00	0.00	0.00
Choroid Plexus	0.00	0.00	0.00	0.00	0.00	0.00	0.00	0.00	0.00	0.00
Cerebellum	0.00	0.00	0.00	0.00	0.00	0.00	0.00	0.00	0.00	0.00
Brain Stem	0.00	0.00	0.00	1.40	0.00	0.00	0.00	0.00	0.00	0.00
Sciatic Nerve	0.00	0.00	0.00	0.00	0.00	0.00	0.00	3.00	0.00	0.00
Trigeminal Ganglia	0.00	0.00	10.20	0.00	0.00	1.35	0.00	0.00	0.00	0.00
Thoracic DRG	1.22	NA	0.00	0.00	0.00	0.00	0.00	0.00	0.00	0.00
Lumbar DRG	0.00	0.00	0.00	1.40	0.00	0.00	0.00	0.00	0.00	0.00
Thoracic Spinal Cord	0.00	1.26	0.00	0.00	0.00	0.00	0.00	3.47	0.00	0.00
Lumbar Spinal Cord	0.00	0.00	0.00	0.00	0.00	0.00	0.00	0.00	0.00	0.00

NA= Not available

Table S4. Longitudinal CSF viral load measurements (RNA copies/mL, * indicates 1 out of 2 replicates detected).

Sample ID	Before ART	2 weeks after ART	2 months after ART	Necropsy
**KE26**	NA	9,457	Not Detected	169
**MD24**	23,578	12,455	Not Detected	Not Detected
**JM96**	280,390	3,824	Not Detected	Not Detected
**KK68**	47,514	67	Not Detected	Not Detected
**JV38**	24,412	2,848	232*	790
**MJ21**	284,815	466	76*	45*
**MC43**	12,759	1,681	Not Detected	68
**MN45**	293,687	229	Not Detected	38
**KB56**	22,561	77*	Not Detected	Not Detected
**MI17**	173,323	623	119*	37

NA= Not available

The limit of quantification (LOQ) for this assay is 62 RNA cp/mL (1.79 log10) with 0.5 mL plasma. Values below the LOQ may be outside of the 95% confidence interval for reproducibility.

Table S5. SIV barcode and gRNAs sequences.

**Barcode**	
vpx-barcode-vpr	-ACGCGAGCCATGNNNNNNNNNNCGATGCGCGCGT- or<br>-ACGCGCGCATCGNNNNNNNNNNCATGGCTCGCGT-
**SIV gRNA PAM**	
Ψ_112	ACGGAGTGCTCCTATAAAGGCGCGGGT
Gag_468	AAGCCCGAGAACATTAAATGCCTGGGT

Table S6. Primer sequences.

SIV GAG F	GCTCCATTAGTGCCAACAGG
SIV GAG R	ACCACTAGGTGTCTCTGCAC
SIV GAG P FAM	TGCGTCATCTGGTGCATTCACGCA
SIV ENV F	CTACTGGTGGCACCTCAAGA
SIV ENV R	AGCGGTCAGCGTCAACGA
SIV ENV P HEX	TGTGCTAGGGTTCTTGGGTT
SIV POL F	TTCTGCCTCAGGGATGGAAG
SIV POL R	CCCTGTCATGTTCCAGGTCT
SIV POL P FAM	TTGCCTTCCTGAAGGGTTCTAGCACA
SIV VPR F	AAGGACCACAAAGGGAACCA
SIV VPR R	CCTCAAGGGTGTCTCCATGT
SIV VPR P FAM	CCTCGCTTGCTAACTGCACTTGGT
RM TERT F	GAGCTGAGATTGTGCCCTTG
RM TERT R	CCATTTGCTGCTCTCTGCTC
RM TERT P HEX	CCAGCACAGATCCTGGTCCCGT
Ψ gRNA F	GACGGAGTGCTCCTATAAAGGC
GAG gRNA F	GAAGCCCGAGAACATTAAATGC
Scaffold R	CGCCAACAAGTTGACGAGAT
Scaffold P FAM	CAGAATCTACTAAAACAAGGCAAA
SaCas9 F	CGACATCAAGGACATTACCG
SaCas9 R	GCTGCTCTGGTAGATGGTCA
SaCas9 P FAM	AACGCCGAGCTGCTGGATCA
IPDA POL F	GCAGGGATAGAGCACACCTTTG
IPDA POL R	CTATGGTTTCTACTGAATTTGCTTGTTC
IPDA POL P	TTTCAGGTGGTGATTCA
IPDA POL P	TAGGTGGTGATTTATT
IPDA ENV F	CCTCAATAAAGCCTTGTGTAAAATTATC
IPDA ENV R	GTTGTTGATGATTTTGTCAATCCC
IPDA ENV P HEX	TGCATTACTATGAGATGC
IPDA ENV P	TGCATTACTATAAAATGC
Excision 1st F	GCTAGACTCTCACCAGCACT
Excision 1st R	CTGATCCTGACGGCTCCCTA
Excision Nested	CGCCTGGTCAACTCGGTACTC
Excision Nested	TCTGCAGCCTCCTCGTTTAT
Barcode 1st F	GGAGAAGAGACAATAGGAGAGG
Barcode 1st R	GAGCGCTCGTTGGAGGATTC
Barcode	CTACACGACGCTCTTCCGATCT AAACCACCTACCAAGGGAGC
Barcode	AGACGTGTGCTCTTCCGATCT CCTCAAGGGTGTCTCCATGT
SIV-NFL-For	CAAGCAGAAGACGGCATACGAGATNNNYRNNNYRNNNYRNNNatatacttagaaaaggaagaaggcatcata
SIV-NFL-Rev	AATGATACGGCGACCACCGAGATCNNNYRNNNYRNNNYRNNNgccttagcctttttcggagttcttc
OntAdapter-For	TTTCTGTTGGTGCTGATATTGCCAAGCAGAAGACGGCATACGAGAT
OntAdapter-	ACTTGCCTGTCGCTCTATCTTCAATGATACGGCGACCACCGAGATC

i501	AATGATACGGCGACCACCGAGATCTACACTATAGCCTACACTCTTTCCCTACACGACGCTCTTCCGATC
i502	AATGATACGGCGACCACCGAGATCTACACATAGAGGCACACTCTTTCCCTACACGACGCTCTTCCGAT
i503	AATGATACGGCGACCACCGAGATCTACACCCTATCCTACACTCTTTCCCTACACGACGCTCTTCCGATC
i504	AATGATACGGCGACCACCGAGATCTACACGGCTCTGAACACTCTTTCCCTACACGACGCTCTTCCGAT
i505	AATGATACGGCGACCACCGAGATCTACACAGGCGAAGACACTCTTTCCCTACACGACGCTCTTCCGAT
i506	AATGATACGGCGACCACCGAGATCTACACTAATCTTAACACTCTTTCCCTACACGACGCTCTTCCGATC
i507	AATGATACGGCGACCACCGAGATCTACACCAGGACGTACACTCTTTCCCTACACGACGCTCTTCCGAT
i508	AATGATACGGCGACCACCGAGATCTACACGTACTGACACACTCTTTCCCTACACGACGCTCTTCCGATC
i509	AATGATACGGCGACCACCGAGATCTACACGACGACCTACACTCTTTCCCTACACGACGCTCTTCCGATC
i510	AATGATACGGCGACCACCGAGATCTACACTAATCGGCACACTCTTTCCCTACACGACGCTCTTCCGATC
i511	AATGATACGGCGACCACCGAGATCTACACTACAGCCTACACTCTTTCCCTACACGACGCTCTTCCGATC
i512	AATGATACGGCGACCACCGAGATCTACACTATAATGAACACTCTTTCCCTACACGACGCTCTTCCGATC
i513	AATGATACGGCGACCACCGAGATCTACACCAGGCGAGACACTCTTTCCCTACACGACGCTCTTCCGAT
i514	AATGATACGGCGACCACCGAGATCTACACCATGGCTAACACTCTTTCCCTACACGACGCTCTTCCGATC
i515	AATGATACGGCGACCACCGAGATCTACACATGAGCGTACACTCTTTCCCTACACGACGCTCTTCCGAT
i516	AATGATACGGCGACCACCGAGATCTACACGGTAGCACACACTCTTTCCCTACACGACGCTCTTCCGAT
i701	CAAGCAGAAGACGGCATACGAGATACCGTGATGTGACTGGAGTTCAGACGTGTGCTCTTCCG
i702	CAAGCAGAAGACGGCATACGAGATACACATCGGTGACTGGAGTTCAGACGTGTGCTCTTCCG
i703	CAAGCAGAAGACGGCATACGAGATACGCCTAAGTGACTGGAGTTCAGACGTGTGCTCTTCCG
i704	CAAGCAGAAGACGGCATACGAGATACTGGTCAGTGACTGGAGTTCAGACGTGTGCTCTTCCG
i705	CAAGCAGAAGACGGCATACGAGATACCACTGTGTGACTGGAGTTCAGACGTGTGCTCTTCCG
i706	CAAGCAGAAGACGGCATACGAGATACATTGGCGTGACTGGAGTTCAGACGTGTGCTCTTCCG
i707	CAAGCAGAAGACGGCATACGAGATACGATCTGGTGACTGGAGTTCAGACGTGTGCTCTTCCG
i708	CAAGCAGAAGACGGCATACGAGATACTCAAGTGTGACTGGAGTTCAGACGTGTGCTCTTCCG
i709	CAAGCAGAAGACGGCATACGAGATACCTGATCGTGACTGGAGTTCAGACGTGTGCTCTTCCG
i710	CAAGCAGAAGACGGCATACGAGATACAAGCTAGTGACTGGAGTTCAGACGTGTGCTCTTCCG
i711	CAAGCAGAAGACGGCATACGAGATACGTAGCCGTGACTGGAGTTCAGACGTGTGCTCTTCCG
i712	CAAGCAGAAGACGGCATACGAGATACTACAAGGTGACTGGAGTTCAGACGTGTGCTCTTCCG
i713	CAAGCAGAAGACGGCATACGAGATACTTGACTGTGACTGGAGTTCAGACGTGTGCTCTTCCG
i714	CAAGCAGAAGACGGCATACGAGATACGGAACTGTGACTGGAGTTCAGACGTGTGCTCTTCCG
i715	CAAGCAGAAGACGGCATACGAGATACTGACATGTGACTGGAGTTCAGACGTGTGCTCTTCCG
i716	CAAGCAGAAGACGGCATACGAGATACGGACGGGTGACTGGAGTTCAGACGTGTGCTCTTCCG

Table S7. Genomic coordinates of nominated predicted off-target sites assayed by rhAmpSeq.

Guide Name	DNA	Chr.	Dir.	Location	score	Mismatch	Bulge
Gag468.188	AAGagaGAGAACATTAgATGCCCGAGT	chr8	+	129646327	3.81	4	0
Gag468.1864	AAGaCCGtcAACATTAAATGCTGGAGA	chr8	+	74466804	3.15	4	0
Gag468.2361	AAGCCACcAGAAaATTAAgTGCCAGAAT	chr5	+	142783769	4.78	3	1
Gag468.2797	AAG-CCtAGAACATTAtATGCAAGGAA	chr6	+	33186275	5.17	3	1
Gag468.3388	AAGa-CaAGAACATTAcATGCATGAAT	chr1	-	68264642	3.84	3	1
Gag468.3487	cAcCCC-AGAACATaAAATGCTGGAGT	chr1	-	90854215	2.25	3	1
Gag468.3875	AAGCCCaATGAcCATTAcATGCTGGGAT	chr1	-	165565036	3.94	3	1
Gag468.4060	AtaCtaGA-AACATTAAATGCACGAAT	chrX	+	7478665	2.39	4	1
Gag468.4398	AAGaCtG-GcAgATTAAATGCCAGAAT	chrX	-	62915993	2.13	4	1
Gag468.7037	AAcCCCaAGATACATTAgATGCCGGAGT	chr1	+	212407024	3.69	3	1
Gag468.7156	AAGtCaGAGAcCATTcAATGCAGGAAT	chr10	-	14701398	2.74	4	0
Gag468.7383	tAGCCgGgGcACATaAAATGCTGGGGT	chr10	+	63679751	2.17	5	0
Gag468.8142	AgGCaCGtGAggATTAAATGCAAGAAT	chr13	-	30597702	2.22	5	0
Gag468.8966	AAtCaaGAGAcCATTAAATGCCAGAAT	chr14	+	76605106	1.49	4	0
Gag468.9400	AAGCaaGAG-ACAcTAAATGCCAGGGT	chr15	+	39545550	2.82	3	1
Gag468.9813	AAGaCgG-GAACAaTAAATGCTGGGGT	chr16	+	6018724	2.18	3	1
Gag468.10211	AAGaCaGAGAtCATTAAATGtAAGGAT	chr17	+	15401964	6.84	4	0
Gag468.11495	AAG-CtGAGAACAaTAAAaGCGTGGGT	chr2	-	30837452	5.95	3	1
Gag468.11808	AAGCACatAGAACATTAAATGCTAGAGA	chr2	-	96069761	3.75	3	1
Gag468.12030	tAtCaCaAGAA-ATTAAATGCAGGAGT	chr2	-	140752403	1.82	4	1
Gag468.12674	AAaCCCcAGAACA-TAAAgGCAAGGGT	chr20	-	71565886	5.95	3	1
Gag468.13583	AAGCCCcAGgAaATTAAATGCAAGGAC	chr3	-	167015911	3.26	4	0
Gag468.14061	AtGgttGAGAAgATTAAATGCCAGGAT	chr4	+	69816156	2.33	5	0
Gag468.14698	AAG-gCaAGAACATaAAATGCAAGGAT	chr5	-	21133450	2.84	3	1
Gag468.14829	tAGtaCaAGAtCATTAAATGCTTGAGT	chr5	+	50382941	1.63	5	0
Ψ112.461	tCtGAGTGCTCC-ATAAAtGCAGGGAT	chr13	+	13583359	5.54	3	1
Ψ112.1278	gCtGgGTGCTgtTATAAAGGCATGGAT	chr10	+	79479345	2.1	5	0
Ψ112.1523	AaaaAcTcCTCCTATAAAGGCGTGGGT	chr11	+	42072377	1.82	5	0
Ψ112.2484	cCaGAGccCTCaTATAAAGGCTGGAAT	chr9	+	73036870	1.87	5	0
Ψ112.3154	cCtGgGT-CTCCgATAAAGGCTGGAGT	chr1	-	38951803	1.94	4	1
Ψ112.3637	ACtGAGTGCcCCTA-AgAGGCAGGGAT	chr1	-	172494503	4.59	3	1
Ψ112.3900	AgGGAGTtCTCC-ATAcAGGCTGGGAT	chr1	+	220144280	3.96	3	1
Ψ112.4440	tgGcAGTGC-CCTATAAAGGCCTGAGT	chr14	-	49910403	1.73	3	1
Ψ112.4469	gCacA-TGCTCCcATAAAGGCCAGGAT	chr14	-	55638045	2.38	4	1
Ψ112.4645	AacGAaTGCATtCTATAAAGGCAGGGGT	chr14	+	104654523	2.41	4	1
Ψ112.5896	ACtGctTcCTCtTATAAAGGCAAGAAT	chr2	+	112791194	1.92	5	0
Ψ112.6825	AtGGAG-GCTCCTATAAAGtCGTGGGT	chr3	+	63712084	5.84	2	1
Ψ112.6971	AtGaAG-GCTCCTATAAAGtCAGGAAT	chr3	+	94988327	5.99	3	1
Ψ112.7215	ACGTcAaTGCTCCTATAAAGGCAAGAAG	chr3	-	166373433	3.67	3	1
Ψ112.7741	ACtGtGaGCTCCT-TAAAGGCAGGAAT	chr6	-	42502495	2.12	3	1
Ψ112.8341	A-GGccTGaTCCTATAAAGGCATGAAT	chr7	-	20912766	1.85	5	0
Ψ112.8946	AgGGAGTGCaCCTcTAAAGGCTTGAAG	chr18	-	4656415	3.57	4	0
Ψ112.9260	AgGGAGATGCTtCTATAAgGGCATGAAT	chr19	+	9683101	4.51	3	1
Ψ112.9911	ACtGtGTGCTCCT-TgAAGGCAGGGAT	chr4	+	165529910	3.81	3	1
Ψ112.10613	ACaGAGTG-TCCTATAAtGGCAAGAAC	chr7	-	79468216	5.91	3	1
Ψ112.10754	AaGGAGTGaTCCTATAAgGaCCTGGGT	chr7	-	114225839	8.39	4	0
Ψ112.10978	ACaGAGT-CTCCaAaAAAGGCTGGAGT	chr7	+	156975163	2.97	3	1
Ψ112.11009	ACaGAGaGCTCCT-TcAAGGCAGGAAT	chr7	-	163600501	3.85	3	1
Ψ112.8536	cCtGAGgGCcCCTAgAAAGGCAAGGGT	chr16	+	72658083	2.09	5	0

Nominated off-target sites for the psi and gag guide RNAs are listed with their chromosomal coordinates. Sites are presented in the same order as displayed along the x-axis of Figure S5. The predicted sites Ψ112.8536 is not assayed, as no primers could be generated using the IDT rhAmpSeq design tool.
