# Supplementary material for: In vivo genome editing of central nervous system SIV reservoirs in ART-suppressed rhesus macaques
Source: Mol Ther Nucleic Acids. 2026 Aug 14;37(3):103063. doi: 10.1016/j.omtn.2026.103063 (PMC13560538; doi:10.1016/j.omtn.2026.103063)
Supplement: Supplementary file 2 [file mmc2.pdf]

Molecular Therapy
Nucleic Acids
Original Article

In vivo genome editing of central nervous system SIV reservoirs in ART-suppressed rhesus macaques

Hong Liu,[1] Shuren Liao,[1] Chen Chen,[1] Wenwen Huo,[2] Zahra Safaei,[1] Angela Rocchi,[1] Yuru Huang,[1] Ilker K. Sariyer,[1] Mackenzie E. Collins,[3] Jill M. Lawrence,[3] Will Dampier,[3] Michael R. Nonnemacher,[3] Brian Wigdahl,[3] Tricia H. Burdo,[4] Jennifer Gordon,[2] Mark Lewis,[5] Cheri A. Lee,[5] and Kamel Khalili[1]

[1]Center for Neurovirology and Gene Editing, Department of Microbiology, Immunology and Inflammation, Lewis Katz School of Medicine at Temple University, 3500 N. Broad Street, 7th Floor, Philadelphia, PA 19140, USA; [2]Excision BioTherapeutics, Inc., 499 Jackson Street, San Francisco, CA 94111, USA; [3]Center for Molecular Virology and Translational Neuroscience, Institute for Molecular Medicine and Infectious Disease, Department of Microbiology and Immunology, Drexel University College of Medicine, 2900 W Queen Ln, Philadelphia, PA 19129, USA; [4]Rutgers Institute for Translational Medicine and Science, Robert Wood Johnson Medical School, Rutgers, The State University of New Jersey, 89 French Street, Suite 4275, New Brunswick, NJ 08901, USA; [5]Bioqual, Inc., 9600 Medical Center Drive, #101, Rockville, MD 20850, USA

Latent human immunodeficiency virus type 1 (HIV-1) reservoirs in the central nervous system (CNS) may sustain viral persistence and neuroinflammation contributing to HIV-associated neurocognitive disorders (HAND) despite suppressive ART. AAV9-delivered CRISPR has successfully edited SIV proviral DNA in peripheral tissues with acceptable safety profiles, but the extent of *in vivo* genome editing in the brain remains unclear. Using SIV-infected rhesus macaques, we mapped intact proviral DNA across CNS regions and tested systemic AAV9-CRISPR-Cas9 targeting conserved sites within Ψ packaging signal and Gag region. Ten adult rhesus macaques were infected with genetically barcoded SIVmac239, suppressed with ART, then randomized to receive intravenous AAV9-SaCas9 with dual gRNAs (Ψ + Gag) or a Cas9-only control. At necropsy after viral rebound, SIV genomes were detected in multiple brain regions as well as lymphoid tissues, confirming the CNS as a persistent reservoir during ART. Barcode analysis revealed region-specific patterns consistent with compartmentalized CNS persistence. In CRISPR-treated animals, proviral editing was measurable across anatomically distinct CNS sites. These findings demonstrate that intact and potentially replication-competent virus persists in the primate brain under ART and that systemic AAV9-CRISPR can reach and edit proviral DNA in this sanctuary, supporting genome editing as a strategy toward durable remission of CNS reservoirs.

INTRODUCTION

Combination antiretroviral therapy (ART) continuously suppresses human immunodeficiency virus type 1 (HIV-1) replication but does not eradicate the virus because of the persistence of dormant or minimally replicating viral reservoirs.[1,2] These reservoirs are established at the early stage of infection and can drive rapid viral rebound and viremia when therapy is interrupted.[3,4] Among the anatomical reservoir sites, the central nervous system (CNS) represents a particular challenge owing to restricted immune surveillance, limited drug penetration across the blood-brain barrier (BBB), and viral discordance between plasma and cerebrospinal fluid (CSF).[5,6] Infection with HIV-1 is frequently associated with neurological complications, most notably HIV-associated neurocognitive disorders (HANDs), which remain prevalent even in individuals receiving effective ART.[7,8] Although early concepts attributed these disorders primarily to indirect effects of systemic infection and the accompanying inflammatory response, subsequent studies have increasingly supported direct CNS involvement through viral entry across the BBB and infection of resident brain cells.[9,10]

Within the brain, tissue-resident macrophages and microglia are considered major cellular reservoirs that can harbor replication-competent viruses, while astrocytes and other non-classical brain cell types may also be infected, in some cases in a restricted or abortive manner. And such infection can disrupt neuronal function and behavior through persistent inflammation, altered glial support, and local neurotoxicity, thereby contributing to neuronal injury, cognitive decline, dementia, and broader neurological dysfunction.[11,12] These observations raise an important question: can the brain serve as a clinically relevant viral reservoir that sustains persistent proviral DNA despite systemic viral suppression? Early *in situ* and postmortem studies have provided evidence for CNS cellular reservoirs, including detection of HIV-1 nucleic acids in microglia,

Received 17 March 2026; accepted 12 August 2026;
https://doi.org/10.1016/j.omtn.2026.103063.

Correspondence: Kamel Khalili, Ph.D. Department of Microbiology, Immunology, and Inflammation, Center for Neurovirology & Gene Editing, Lewis Katz School of Medicine, Temple University, Philadelphia, PA, USA.
E-mail: kamel.khalili@temple.edu

Published by Elsevier Inc. on behalf of The American Society of Gene and Cell Therapy.
This is an open access article under the CC BY-NC-ND license (http://creativecommons.org/licenses/by-nc-nd/4.0/).

macrophages, astrocytes, and, in rare instances, neurons.[13,14] These findings underscore the brain as a clinically relevant sanctuary site that may sustain latent or low-level viral persistence during ART.

Gene editing offers a promising strategy to directly eliminate integrated proviral DNA. CRISPR-Cas9 systems can be programmed to excise conserved viral sequences and have shown antiviral activity in cell culture and animal models of HIV infection.[15–18] Effective translation of this approach to the CNS, however, requires delivery systems with broad biodistribution and the ability to access brain tissue. Adeno-associated virus serotype 9 (AAV9) has been shown to cross the BBB and transduce neurons and glia in rodents and nonhuman primates, motivating its use for CNS-directed genome editing.[19] In our previous studies using ART-treated rhesus macaques, AAV9-delivered CRISPR has edited SIV proviral DNA in peripheral tissues with an acceptable safety profile, yet its editing activity across defined CNS regions has not been comprehensively assessed.[20,21]

The SIV-infected rhesus macaque is regarded as a gold-standard preclinical model for HIV-1 infection because it closely models viral pathogenesis, reservoir biology, and tissue distribution, enabling rigorous evaluation of cure strategies *in vivo*.[22,23] In addition, genetically barcoded SIVmac239 provides a unique opportunity to examine the clonal composition and diversity of viral reservoirs across tissues.[24] Here, using barcoded SIV in long-term ART-suppressed macaques, we investigated the presence of SIV proviral DNA in multiple regions of the brain and evaluated the potential of systemic AAV9-CRISPR-Cas9 delivery to edit conserved SIV sequences within the Ψ packaging signal and Gag region in CNS reservoirs elements and assess regional CNS proviral editing alongside clonal dynamics. Further comparison of the genetic diversity and clonal architecture of viral reservoirs in the brain versus peripheral compartments provides insight into the brain as a potential reservoir site and informs gene-editing strategies aimed at viral elimination from the CNS.

RESULTS

Design and validation of CRISPR-Cas9 gRNAs

To identify optimal target sequences for SIV gene editing, the *in silico* analyses were performed to design gRNAs against conserved regions of the SIV genome. The LTR-targeting gRNA used in our previous studies was excluded to preserve the barcode cassette required for downstream clonal tracking analyses. The candidate gRNAs were evaluated using *CRISPOR* and *Benchling* platforms for bioinformatic predictions of on-target activity and off-target risk. Two high-scoring gRNAs targeting Ψ_112 and Gag_468 were selected for the downstream studies (Figure 1A). The 3D8 cell line, which harbors a single integrated SIV copy per cell, was used to functionally verify the activity of gRNAs via SaCas9-CRISPR ribonucleoprotein (RNP) nucleofection. The dual-gRNA SaCas9 complex electroporated into 3D8 cells resulted in the expected Ψ-Gag segment deletion as verified by excision PCR (Figure 1B) and Sanger DNA sequencing (Figure S1). Based on the reduction in the Gag-to-Env signal ratio detected by droplet digital PCR (ddPCR), excision of Gag-containing segment occurred in approximately 30% of total SIV genomes (Figure 1C). Additionally, the macaque microglia cells M1A infected with SIVmac239 were used to test the CRISPR-Ψ-Gag dual gRNA system and showed editing outcomes similar to those observed in 3D8 cells (Figure S2). The two gRNAs were cloned into the pX601 vector, and SaCas9 expression was placed under the control of the EF1α promoter. This modification preserved SaCas9 expression at levels comparable to those achieved with the original CMV promoter while reducing vector size to better accommodate AAV9 packaging limitations (Figure S3). The AAV9 vector containing SaCas9 and two gRNAs were tested in the 3D8 cells. PCR analysis confirmed successful cleavage at the Ψ and Gag gRNA sites, resulting in deletion of the intervening SIV genomic segment (Figure 1D). To further confirm the impact of the Ψ and Gag in the 3D8 cells, SIV-near full length (NFL) PCR, which amplifies an approximate 7 kb segment of the proviral genome, was used to quantify the rate of small indels and targeted deletions generated. Each UMI-consensus read was aligned and classified as either clean, contained an indel, or was part of a small indel or large deletion. A pattern of indels and targeted deletions were observed at the target sites in treated samples (Figure 1E). In the Ψ-Gag treated samples, only 7.9% of the reads had no evidence of edits vs. 60% and 69% in untreated or Cas9 only controls, respectively. The frequency of reads containing an indel at either site was consistent across samples but the number of reads with indels at both sites was 15% in treated vs. 5.66% and 1.9% in untreated or Cas9 only controls, respectively. The site-specific excision event (targeted deletion) was found in 18% of reads from Ψ-Gag treated samples and was not observed in either control. Interestingly, we observed that 5% of reads contained a deletion where only one edge matched a target while the other edge did not. This was only observed in the Ψ-Gag treated samples and never observed in either control. There was also an increase in the frequency (21%) of unintended proviral deletions, where neither end matched a target site in Ψ-Gag treated samples, compared to only 1.8% and 2.8% in untreated or Cas9 only controls, respectively (Figure 1F). These results support the activity of the selected gRNA pair and provide a rationale for its subsequent evaluation *in vivo*.

In vivo AAV9-CRISPR-Cas9 administration in SIV-infected rhesus macaques

Following SIVmac239M2 infection and long-term ART suppression, ten rhesus macaques received AAV9 vectors encoding CRISPR-Cas9 with (five Ψ-Gag CRISPR) or without (five Cas9 only) the validated gRNAs (Figure 2A). ART was discontinued one week after AAV9 infusion to initiate analytical treatment interruption (ATI) and assess viral rebound. Following viral rebound, tissues were collected by anatomical regions from which DNA was prepared for the next-step analyses. The ddPCR analysis showed that SIV Pol signals were most prominent in lymph nodes (Figure 2B). While SaCas9 signals, which indicate AAV9 vector delivery and persistence of vector-associated sequences, were enriched primarily in the liver but were also detected across multiple other tissues (Figure 2C). Notably, SaCas9

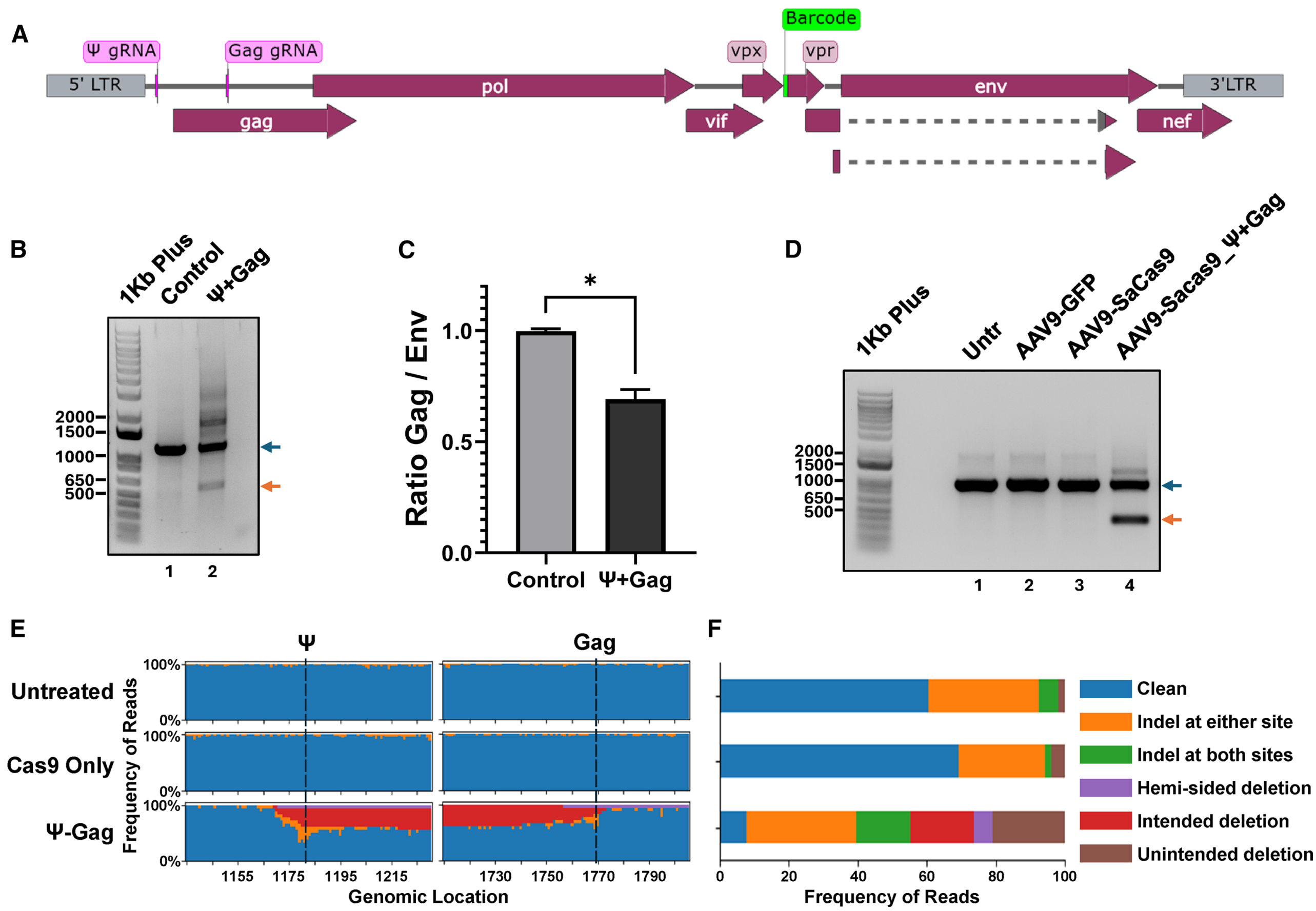

Figure 1. Design and validation of gRNAs targeting the SIV genome

(A) Schematic of the SIV genome showing the locations of the Ψ (psi) and Gag gRNA target sites and the barcode region. (B) PCR across the Ψ-Gag interval using DNA from 3D8 cells electroporated with Cas9 RNPs carrying both gRNAs. Dual-gRNA editing generated a smaller amplicon consistent with excision of the 590-bp Ψ-Gag segment (the orange arrow indicates the excision band, and the blue arrow indicates the full-length band). (C) Excision efficiency quantified by ddPCR. A reduced Gag-to-Env signal ratio indicated deletion of the SIV Gag-containing segment in 3D8 cells. Data are presented as mean ± SD. $^{*}p < 0.05$. (D) Nested PCR confirmation of Ψ-Gag excision in AAV9-CRISPR-treated 3D8 cells, showing a truncated product consistent with deletion (the orange arrow indicates the excision band, and the blue arrow indicates the full-length band). (E) The frequency of clean (blue), small indel (orange), hemi-sided deletions (purple), intended deletion (red), or unintended deletion (brown) at each base surrounding the target sites. The dashed line indicates the targeted cut sites. (F) A stacked bar plot of the relative frequency of classified reads.

signal levels were substantially higher than SIV Pol signals in the tissues analyzed. The Ψ (Figure 2D) and Gag (Figure 2E) gRNA sequences were found exclusively in the five animals treated with AAV9-CRISPR.

SIV proviral editing in multiple tissues of AAV9-CRISPR-treated rhesus macaques

CRISPR mediated removal of SIV DNA spanning Ψ to Gag was assessed by nested PCR. SIV deletions within this region were detected across tissues from five Ψ-Gag CRISPR-treated monkeys but were absent in Cas9-only control animals, underscoring the requirement of gRNA inclusion (Figure 3A). These deletion products were detected most frequently in lymph node tissues, consistent with the prominent SIV Pol signals observed in lymph nodes by ddPCR (Figure 2B). In contrast, tissues with strong SaCas9 signals but low or undetectable SIV DNA, such as the liver in most animals, did not show detectable editing by PCR-based assays. These findings indicate that detectable proviral editing depends on the simultaneous presence of both the CRISPR editing machinery and sufficient SIV DNA substrate within the sampled tissue. Besides, nested PCR targeting the SIV Pol region did not detect any deletion products in tissues from either CRISPR-treated or control animal (Figure S4), suggesting that the deletions observed in the Ψ-Gag region were specifically mediated by AAV9-delivered CRISPR. Moreover, rhAmpSeq analysis detected no statistically significant increase in indels or structural variants at any nominated predicted off-target site in CRISPR-treated animals compared with control animals, suggesting that editing with the Ψ_112 and Gag_468 guide pair did not produce detectable off-target chromosomal alterations within the analyzed sites and assay sensitivity, thereby supporting the safety profile of the AAV9-CRISPR strategy (Figure S5; Table S1). Sanger sequencing of excised amplicons confirmed SIV editing in these

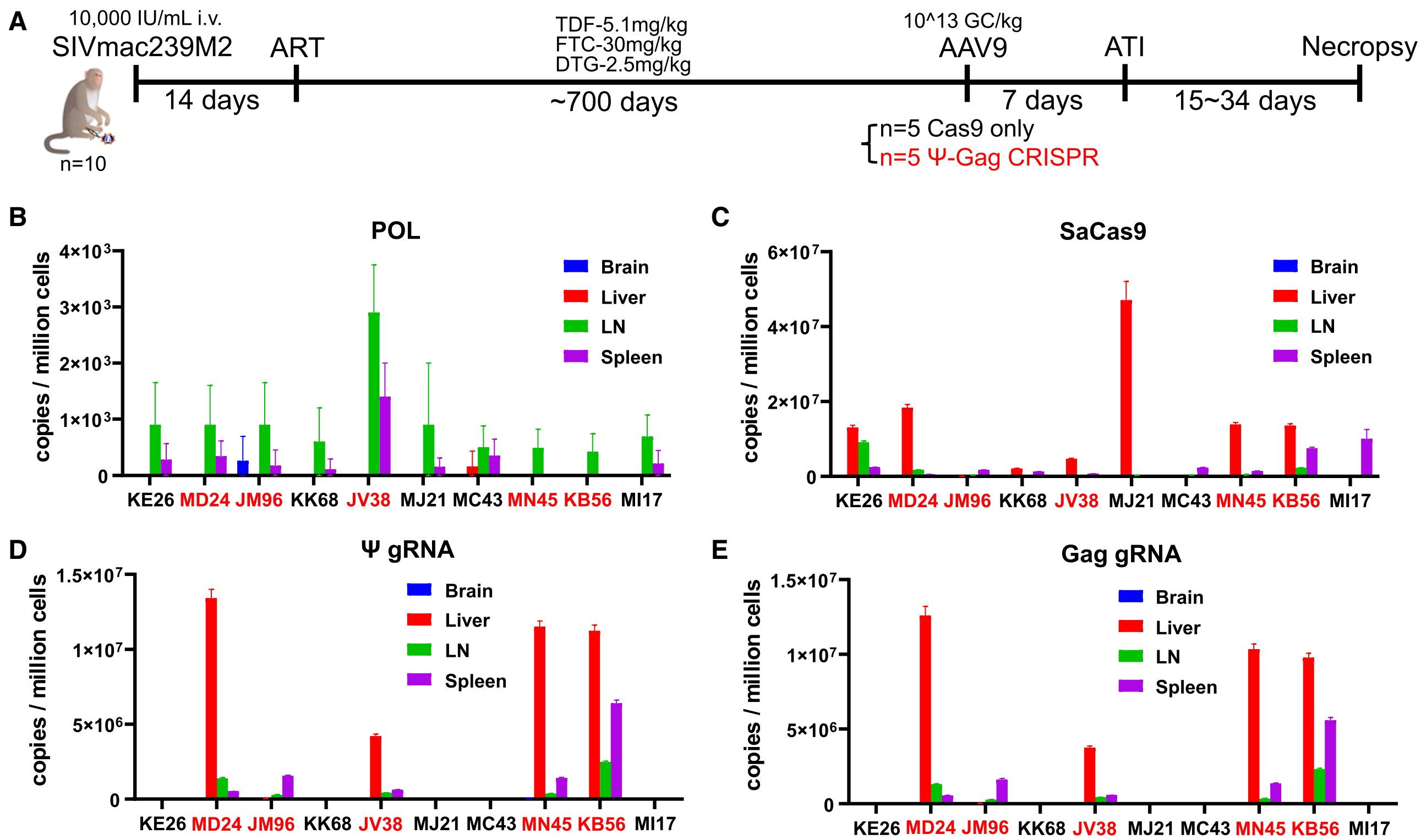

Figure 2. SIV and CRISPR detection in AAV9-CRISPR-Cas9 treated monkeys

(A) Timeline illustrating experimental design wherein ten rhesus macaques were intravenously inoculated with barcoded SIVmac239M2 and maintained on daily subcutaneous ART (tenofovir, emtricitabine, and dolutegravir) for about 24 months. Five animals (MD24, JM96, JV38, MN45, and KB56 shown in red) received a slow intravenous infusion of AAV9 vector delivering CRISPR-Cas9 with dual gRNAs targeting Ψ and Gag (10^{13} GC/kg); the remaining five animals (KE26, KK68, MJ21, MC43, and MI17 shown in black) received a control AAV9 vector expressing Cas9 only. ART was withdrawn one-week post-infusion, and necropsies were performed at the onset of detectable viral rebound. Regionally extracted DNA from all ten animals was quantified by ddPCR for SIV Pol (B), SaCas9 (C), gRNA Ψ (D) and gRNA Gag (E) levels. Data in panels (B)–(E) are presented as mean ± SD.

gRNA-treated tissues, while SIV cleavage in the brain tissues showed particularly precise on-target junctions consistent with the predicted CRISPR cut sites (Figure 3B), potentially reflecting greater co-occurrence of CRISPR components and SIV DNA substrate within those sampled tissues. To further confirm these results, the PCR products were subjected to nanopore sequencing to determine the frequency of targeted deletions from treated and untreated animals. The site-specific excision deletion was found in 47% of reads from MD24-Brain and 0% in all other tested samples. The frequency of hemi-targeted deletions and unintended proviral deletions also increased to 0.5% and 10%, respectively, when compared to 0.03% and 0.3% rates seen in the untreated KK68 sample (Figure 3C). These findings motivated a focused investigation of nervous system compartments from all these animals in the following study.

Distribution of SIV and Cas9 in various brain regions

Total 13 brain regions from each animal were examined for SIV Pol, Vpr, and SaCas9 DNA. SIV Pol was most frequently detected in prefrontal cortex and choroid plexus, while Vpr was most frequent in hypothalamus and prefrontal cortex (Figure 4A). Both SIV markers were broadly distributed across the brain tissues, as all ten animals show SIV positivity in at least two or three brain regions (Table S2). SaCas9 DNA was detected in over 80% of sampled brain regions, indicating widespread vector access. Using intact proviral DNA assay (IPDA), intact SIV genomes were identified in classical viral enrich sites (e.g., lymph nodes and spleen) as well as in CNS regions including the prefrontal cortex and hypothalamus (Figure 4B; Table S3). However, the intact full-length viral genomes were relatively rare in brain tissues, suggesting that a substantial proportion of CNS-associated viral DNA may be defective and contain deletions. Thus, assays targeting different regions of the SIV genome, such as Pol and Vpr, may yield non-concordant detection patterns, as shown in Figure 4A. These data suggest the presence of replication-competent viruses in various brain regions despite ART intervention, which may serve as an additional reservoir alongside traditional lymphoid tissues.

CRISPR-Cas9 excises SIV provirus in the nervous system

In the five Ψ-Gag CRISPR treated animals, 19 nervous system tissues per animal, including 13 brain, two spinal cord, and four PNS regions, were analyzed for excision-specific amplicons. Some nervous system tissues from these treated animals yielded truncated amplicons when detected by nested PCR (Figure 5A). Sanger sequencing

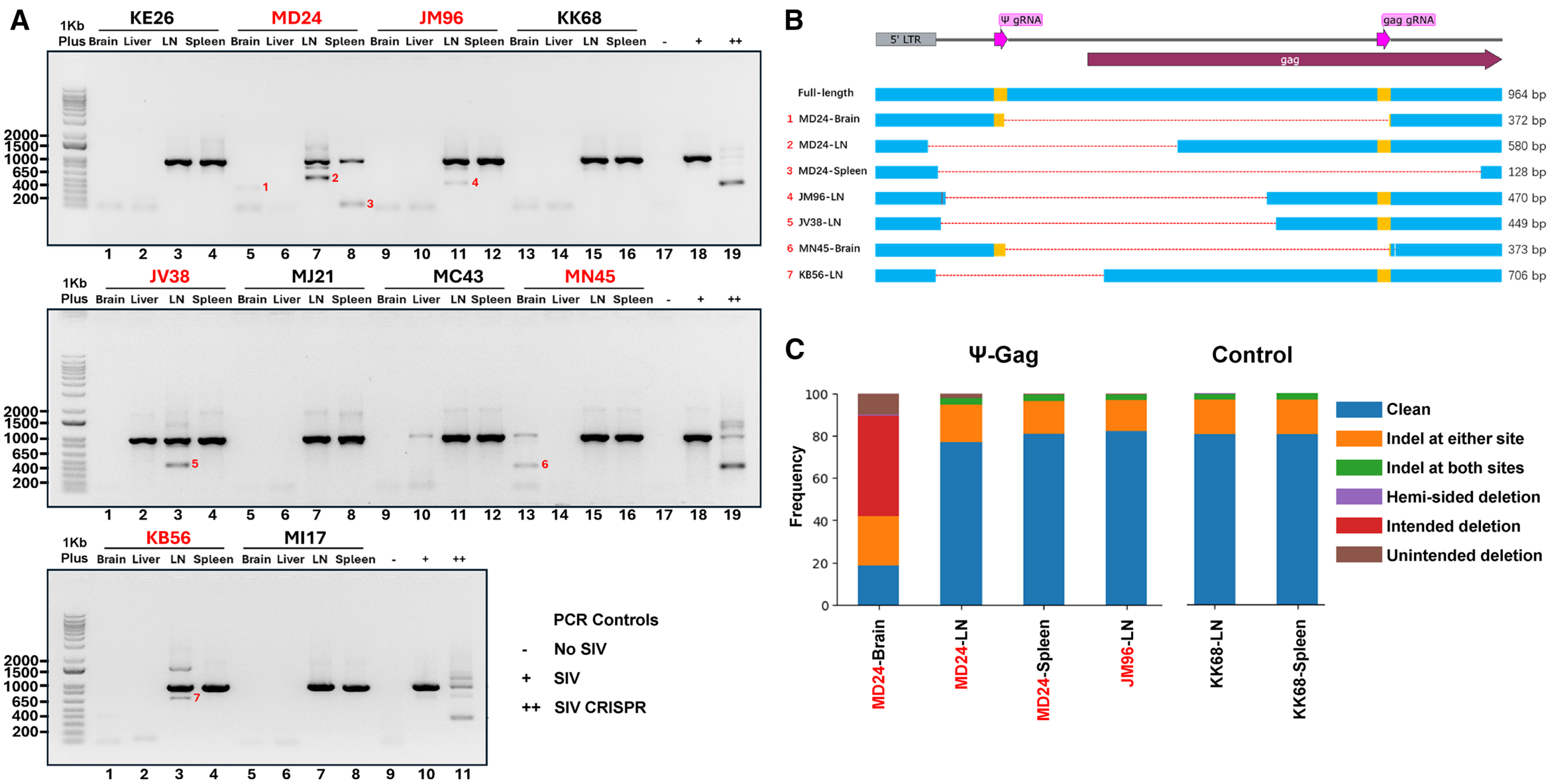

Figure 3. *In vivo* SIV excision by AAV9-delivered CRISPR-Cas9 in ART-suppressed rhesus macaques

(A) Agarose gel electrophoresis of nested Ψ-Gag PCR amplicons confirmed SIV proviral DNA excision in tissues from CRISPR-treated animals (MD24, JM96, JV38, MN45, and KB56 shown in red), but not in control animals (KE26, KK68, MJ21, MC43, and MI17 shown in black). The + and ++ represent 3D8 cells and 3D8 cells treated with CRISPR respectively. (B) Truncated amplicons were validated by Sanger sequencing as shown in the sequence alignment. (C) A stacked barplot of the relative frequency of classified reads from the entire PCR reactions shown in (A) as determined by nanopore sequencing.

confirmed cleavage mostly located at the Ψ and Gag target sites as shown in the representative schematic, which is consistent with previous findings (Figure 5B). To relate proviral editing to regional SIV and AAV levels, excision events were enriched in CNS areas with higher local SIV and SaCas9 DNA—most notably the prefrontal cortex and substantia nigra (Figure 5C), indicating that editing was influenced by both vector biodistribution and substrate abundance. In addition, no significant correlation was observed between necropsy timing and editing outcomes across animals, suggesting that the modest variation in necropsy timing did not account for the observed editing patterns.

Barcode analysis of viral diversity

To investigate SIV clonal dynamics, barcode-tagged sequences were analyzed in various tissues from 4 monkeys (controls: MJ21, KE26; treated: KB56, MN45). High-throughput sequencing revealed limited but distinct populations within individual regions, suggesting that CNS reservoirs can be seeded and maintained at least partially independent from viral persistence in peripheral sites (Figure 6A). Notably, the normalized barcode richness was much lower in peripheral tissues associated with higher rebound virus levels (e.g., PBMC, lymph nodes, spleen) than in nervous system tissues (Figure 6B). These findings illustrate spatial compartmentalization of reservoir populations and support the concept that anatomically distinct reservoirs may evolve uniquely under local constraints and contribute differentially to rebound after ATI.

DISCUSSION

Despite decades of research and the advent of potent ART, the eradication of HIV remains elusive due to the persistence of latent viral reservoirs. Recent reports showed that CD4+ T cells can also be found in the brain, reinforcing the importance of HIV reservoirs in the CNS.[25,26] However, few studies have specifically investigated CNS reservoirs, which are uniquely challenging due to the BBB, cellular heterogeneity, and limited immune surveillance.[27] Our study suggests that CNS may also serve as a critical reservoir for SIV, even under long-term ART suppression, aligning with observations in people with HIV-1.[5,6] We detected broad distribution of SIV DNA across multiple brain regions, indicating that virus can disseminate widely within the brain and that proviral DNA can persist in brain cells. IPDA further identified intact genomes in select CNS sites as well as classical lymphoid reservoirs. Notably, intact proviruses were less frequent than total Pol/Vpr signals in the brain, consistent with the known predominance of defective genomes in treated infection and the triphasic decay kinetics of intact SIV genomes during ART.[28] The presence of hypermutated barcodes in some CNS samples further suggests ongoing host-mediated editing and defective integration events in this compartment.[29,30] During specific clinical states, including neurosymptomatic CSF escape under suppressive ART, drug-resistant variants may replicate locally and drive inflammation.[31] Together, these observations reinforce the view that a persistent and heterogeneous CNS reservoir remains present during ART with ongoing potential

A

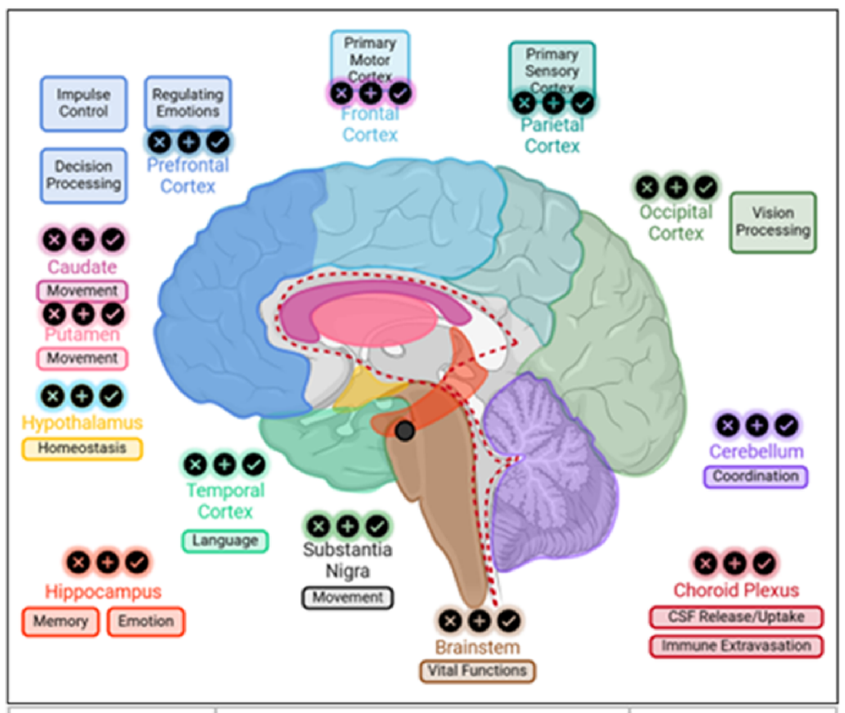

	SIV		AAV9
Region	Pol (X)	VPR (+)	Cas9 (✓)
HIPP	3	6	10
PFC	5	8	9
C	2	4	10
Pu	1	3	8
SN	3	5	9
FC	2	3	9
HYP	2	9	10
ChP	6	6	10
PC	2	5	8
TC	2	3	7
OC	3	6	10
CRB	2	6	10
BS	2	7	9

B

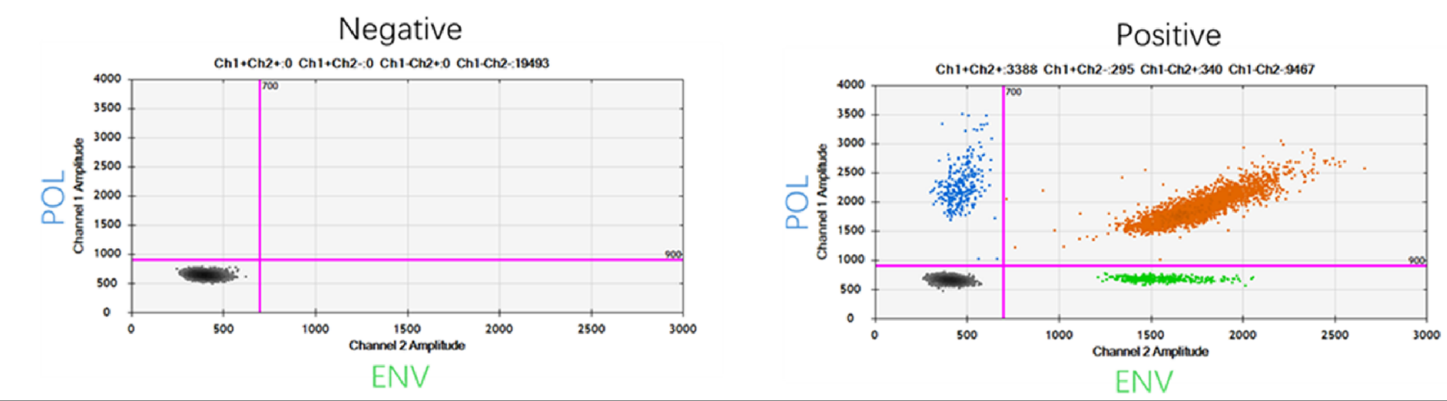

SIV IPDA detected by ddPCR	KE26	MD24	JM96	KK68	JV38	MJ21	MC43	MN45	KB56	MI17	+
Liver					X		X				2
Submandibular LN	X	X	X	X	X	X	X	X	X	X	10
Spleen cells	X	X	X	X	X	X	X	X	X	X	10
PBMC	X		-	X	X	X		X	X	X	7
Prefrontal Cortex					X					X	2
Frontal Cortex					X						1
Basal Ganglia			X								1
Caudate Nuclei											0
Parietal Cortex											0
Hippocampus					X			X			2
Temporal Cortex					X	X					2
Occipital Cortex				X						X	2
Hypothalamus	X							X			2
Substantia Nigra		X									1
Choroid Plexus											0
Cerebellum											0
Brain Stem				X							1
Sciatic Nerve								X			1
Trigeminal Ganglia			X			X					2
Thoracic DRG	X	-									1
Lumbar DRG				X							1
Thoracic Spinal Cord		X						X			2
Lumbar Spinal Cord											0

Figure 4. Detection of SIV proviral DNA and SaCas9 across distinct brain regions

(A) Schematic illustration of 13 anatomically distinct brain regions (top) from which DNA was extracted and analyzed by ddPCR with accompanying table summarizing the number of animals showing positive signals for SIV Pol, SIV Vpr, and SaCas9 in each region (bottom). (B) Representative IPDA analysis shows that multiplex ddPCR simultaneously amplifies the Pol and Env regions, enabling selective detection of intact proviruses while excluding hypermutated or deleted SIV DNA molecules (top). IPDA was performed on total DNA extracted from various tissues of all ten monkeys. Intact SIV proviral DNA was consistently detected in lymph nodes (LNs) and spleen, as well as in several CNS regions (bottom, "x" indicates positive detection of intact proviral DNA, and "–" indicates not determined; CRISPR-treated animals are shown in red and control animals are shown in white).

neuroinflammatory consequences linked to HAND in a subset of individuals.

The barcode analysis revealed overall limited diversity in the CNS and peripheral tissues, suggesting that early founder viruses may establish long-lived reservoirs. Furthermore, CNS viruses exhibit genetic compartmentalization and clonal expansion, indicating that they are seeded independently and evolve under distinct selective pressures.[32,33] The localized reservoirs may arise due to the limited trafficking of immune cells and cART compounds across the BBB, highlighting the importance of targeting distinct CNS niches for viral eradication.[9] In this regard, the use of genetically barcoded virus provides an additional opportunity to compare the genetic diversity of viral reservoirs in brain versus peripheral tissues and to further evaluate the extent to which the CNS may represent a distinct viral compartment.

The use of dual gRNAs targeting Ψ and Gag achieved successful cleavage and excision of proviral DNA in multiple tissues, with no off-target editing detected in the potential sites (Figure S5), supporting the feasibility of systemic AAV delivery for CNS-targeted CRISPR-Cas9 strategies. Importantly, this approach was safe and did not provoke overt inflammation or toxicity. Our results further imply that AAV9-mediated delivery of CRISPR can induce editing of proviral DNA in the brain, primarily through indel formation and, in some instances, albeit at lower frequency, through dual cleavage at Gag and Ψ that results in excision of the intervening viral DNA from integrated host genomic DNA. In tissues from CRISPR-treated monkeys, the dual Ψ and Gag gRNAs produced not only precise excision of the targeted Ψ-Gag intervening segment but also a range of imperfect repair products, including variable breakpoint deletions and small indels at the target sites. Such heterogeneity is expected as NHEJ/MMEJ-mediated end joining pathways following two Cas9-induced double-strand breaks, and similar outcomes have been reported in paired-gRNA deletion strategies and CRISPR approaches designed to target integrated HIV DNA.[34,35] Importantly, even when perfectly rejoined excision products are not recovered, indel-bearing junctions may still functionally inactivate proviral genomes,

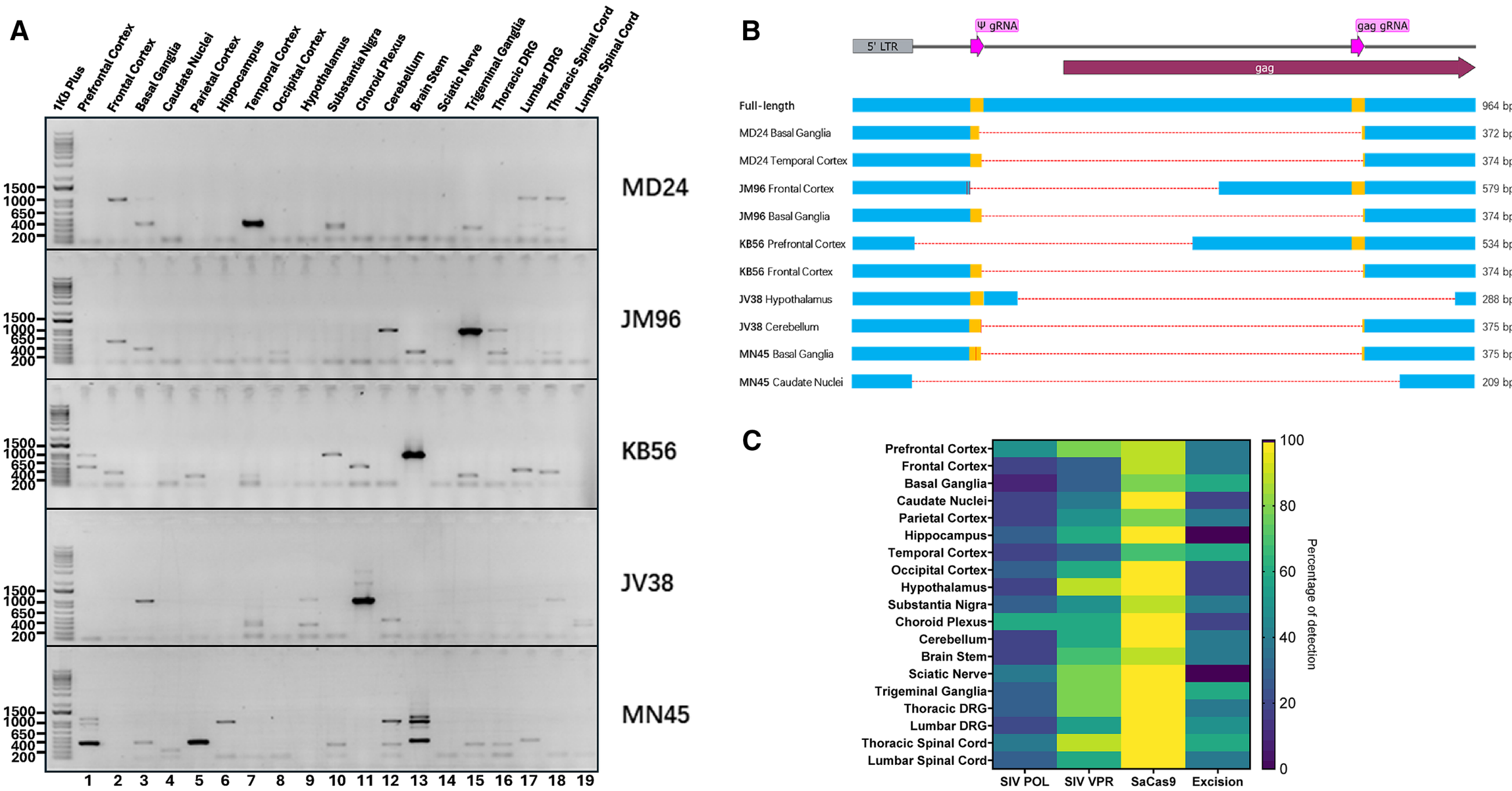

Figure 5. CRISPR-Cas9 mediated excision of SIV DNA in the nervous system of rhesus macaques

(A) PCR-based analysis detected excision of SIV proviral DNA (between Ψ and Gag) in various nervous system regions of the five CRISPR-treated animals, demonstrating successful *in vivo* genome editing. The approximately 1,000 bp band represents the full-length amplicon. The Ψ-Gag deletion is indicated by the presence of the smaller excision band of approximately 400 bp. Bands below 100 bp were considered primer dimers. (B) Representative truncated amplicons were confirmed by Sanger sequencing, as shown in the sequence alignment. (C) Heatmap summarizing the distribution of SIV Pol, Vpr, Cas9, and confirmed excision events across nervous system tissues from the five treated animals.

supporting the biological relevance of these mixed editing outcomes. At the same time, our findings do not exclude the possibility that some viral DNA detected in the brain represents genetically defective proviruses. However, the identification of precise excision products spanning the planned CRISPR target sites strongly supports authentic *in vivo* editing of integrated viral DNA within brain tissues and cells. Thus, while defective genomes may contribute to the total proviral signal, the recovery of predicted excision junctions provides compelling molecular evidence that CRISPR-mediated targeting occurred directly within CNS-associated proviral DNA.

While our results demonstrate the potential of CRISPR-Cas9 to target CNS viral reservoirs, several limitations should be addressed in future studies. Because this study included only male rhesus macaques due to cohort limitations, future translational studies should include both male and female animals to determine whether sex-associated differences influence CNS reservoir persistence, AAV9-CRISPR-Cas9 delivery, and editing outcomes. The cell lines used here were intended to validate the activity of the dual-gRNA CRISPR-Cas9 strategy and confirm targeted editing of the SIV genome. However, the observation under optimized *in vitro* electroporation or transduction conditions may not directly predict *in vivo* CNS effect, where vector delivery, cell-type accessibility, viral DNA abundance, anatomical distribution, and local tissue barriers may all influence the outcomes. Although promising, CRISPR editing did not fully eliminate intact proviral DNA, possibly due in part to limited AAV9-mediated transduction efficiency in certain brain regions.[36] Cas9 was broadly distributed in the brain of AAV9-treated animals, with excision events observed more frequently in the basal ganglia and temporal cortex, while other brain regions such as the hippocampus showed comparatively fewer editing events, likely reflecting variable delivery kinetics of AAV9 tropism or regional differences in vector uptake.[37] Residual intact genomes persisted in some regions of treated animals, emphasizing the need to optimize delivery and editing depth to achieve broader CNS coverage. CSF viral loads at necropsy were very low or undetectable, likely due to prolonged ART-mediated viral suppression and the limited duration of ATI (Table S4). Future studies with systematic longitudinal CSF sampling will be needed to determine whether CNS editing affects the timing and kinetics of CSF viral rebound. Moreover, latency reversal was not induced in this study, which may limit the accessibility of CRISPR-Cas9 to deeply latent proviruses.[38] Combining gene-editing approaches with latency-reversing agents and immunotherapy strategies may improve overall clearance.[39]

The ability to target CNS reservoirs represents a critical advancement in curative HIV research, as many current strategies focus

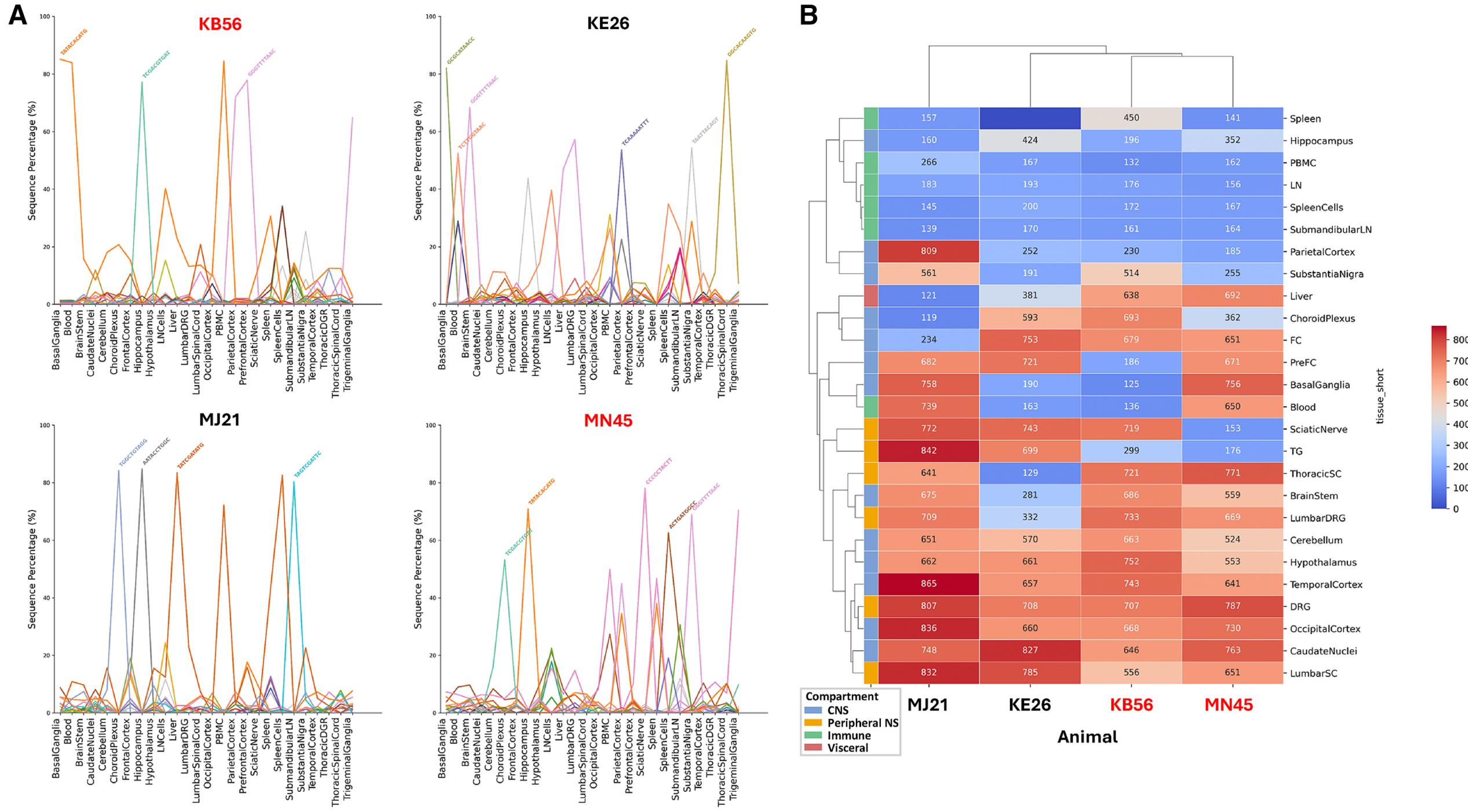

Figure 6. Barcode analysis of viral clonal diversity

(A) Relative abundance of the top-10 most abundant barcode sequences per tissue, shown separately for each animal. For each tissue, the ten barcodes with the highest read counts were identified, and their read percentages were plotted across all tissue types. Each line represents an individual barcode sequence, and colors distinguish barcodes within each figure. Those with >50% sequence percentage were annotated with the first 10 nt barcode sequences. (B) Cluster map of normalized barcode richness across 26 tissues and four animals. Tissues and animals were independently clustered using hierarchical clustering with Euclidean distance and average linkage. Cell colors represent normalized richness values according to the color scale shown (blue: low, red: high). Numerical values indicate the raw rounded integer values of normalized richness. The dendrogram on the top illustrates similarity among animals, while the dendrogram on the left illustrates similarity among tissues based on their barcode richness profiles. Colored bars alongside the tissue dendrogram indicate anatomical compartment: CNS (blue), peripheral nervous system (amber), immune/lymphoid (green), and visceral (red). KE26 Spleen had no available data (shown as 0). Untreated animals shown in black: MJ21, KE26. CRISPR-treated animals shown in red: KB56, MN45.

predominantly on peripheral reservoirs while the CNS remains relatively neglected.[40–42] Longitudinal analyses in ART-suppressed people with HIV-1 further indicate that the proviral reservoir can remain remarkably stable over time,[43] and studies of brain tissue demonstrate persistence of intact proviruses within the CNS compartment.[44] This study therefore helps bridge an important translational gap toward achieving HIV remission or elimination. Our findings highlight the potential of AAV9-CRISPR-Cas9 gene editing as a therapeutic strategy for targeting SIV/HIV reservoirs within the CNS. Successful excision of proviral DNA in ART-suppressed macaques demonstrates that even difficult-to-reach sanctuary sites can be accessed by this approach. Further optimization and combination approaches may improve the likelihood of achieving a functional cure. Overall, this study provides a comprehensive CNS-wide evaluation of *in vivo* SIV excision using a CRISPR strategy in a large animal model.

MATERIALS AND METHODS

Cell culture

The 3D8 cells (obtained through the NIH HIV Reagent Program, Division of AIDS, NIAID, NIH: Simian Immunodeficiency Virus, mac316 infected CEMx174 Cells, Clone 3D8, ARP-13239, contributed by Dr. Mario Roedrer and Dr. Joseph Mattapallil) were maintained in RPMI medium supplemented with 10% fetal bovine serum (FBS) and gentamicin (10 μg/mL; Sigma-Aldrich, St. Louis, MO, USA). The macaque microglia M1A cells were kindly provided by Dr. Yoelvis Garcia-Mesa and Dr. Jonathan Karn at Case Western Reserve University.[45] The cells were cultured in DMEM/F12 medium supplemented with glutamine, 5% FBS, 1× N2, 1× non-essential amino acids (NEAA), 10 mM HEPES, and Plasmocin (2.5 μg/mL) in flasks coated with 0.5% gelatin solution at 37°C for at least 30 min, followed by aspiration before cell plating.

Animal study design

Ten adult male rhesus macaques (Macaca mulatta) were enrolled in this study. All animals were intravenously inoculated with a barcoded SIVmac239M2 (10,000 IU/mL) (a generous gift from Brandon Keele, barcode sequences shown in Table S5) and after two weeks maintained on a daily subcutaneous ART regimen comprising tenofovir (5.1 mg/kg), emtricitabine (30 mg/kg), and dolutegravir (2.5 mg/kg). ART was administered for a minimum of 20 months to ensure sustained viral suppression before CRISPR treatment. All

procedures were approved by the Institutional Animal Care and Use Committee (IACUC) and conducted under protocols at an AAALAC International-accredited facility.

CRISPR-Cas9 vector design

Dual guide RNAs (gRNAs) targeting the conserved Ψ (packaging signal) and Gag (structural polyprotein) regions of the SIV genome were designed based on bioinformatics predictions using *Benchling* and *CRISPOR* tools to ensure high on-target efficiency and minimal off-target activity (Table S5). The two gRNAs were cloned into an AAV9 vector containing *Staphylococcus aureus* (SaCas9) under the control of a EF1α promoter (Figure S3).

RNP-CRISPR electroporation

Guide RNAs were coupled individually with SaCas9 by adding 2.2 μL of sNLS-SaCas9-sNLS Nuclease (10 μg/mL = 80 μM, Aldevron, #9218-0.25MG) to 6 μL of synthetic gRNA (60 μM, CRISPRevolution Custom RNA modified, Synthego) in 11.8 μL of electroporation Buffer R (Neon, Invitrogen). The molar ratio when incubating was 1:2 (9 μM:18 μM) SaCas9: gRNAs. The mixtures were incubated individually for 10 min at room temperature (RT), then combined and added to 2.5×10^6 of target cells in 200 μL of Buffer R. Cells were then mixed gently and electroporated using Neon Electroporation Device set to 1400 V, 1 × 30 ms impulse with 100 μL Neon tip. Cells were immediately plated in 4 mL of pre-warmed growth medium.

AAV9-CRISPR administration

Animals were randomly assigned into two groups: treatment (n = 5) and control (n = 5). The treatment group received a slow intravenous infusion of AAV9-CRISPR vectors containing dual gRNAs (10^{13} genome copies/kg), while control animals received AAV9-Cas9 vectors lacking gRNAs. Following systemic AAV9-SaCas9 administration, ART was discontinued one week later. Animals were monitored longitudinally for plasma viral rebound, and necropsy was performed at the onset of detectable rebound. Because rebound occurred at different times among animals, necropsy was conducted between 15 and 34 days after ART withdrawal. To minimize potential confounding effects associated with variable necropsy timing, all delivery and editing analyses were normalized to tissue input or cell equivalents, where applicable, and quantified using standardized molecular assays performed under identical experimental conditions.

Tissue collection and DNA extraction

Upon confirmation of viral rebound, animals were euthanized and perfused with phosphate-buffered saline (PBS). Tissues including spleen, lymph nodes, liver and peripheral blood mononuclear cells (PBMCs) were harvested. A total of 19 nervous system tissues were collected from each animal, including 15 CNS regions (prefrontal cortex, frontal cortex, basal ganglia, caudate nuclei, parietal cortex, hippocampus, temporal cortex, occipital cortex, hypothalamus, substantia nigra, choroid plexus, cerebellum, brain stem, thoracic and lumbar spinal cord) and four peripheral nervous system (PNS) tissues (sciatic nerve, trigeminal ganglia, thoracic and lumbar dorsal root ganglia). Genomic DNA was extracted from tissues using the NucleoSpin Tissue kits (Macherey-Nagel, Germany) according to the manufacturer's protocol and quantified with the Nanodrop spectrophotometry.

PCR, droplet digital PCR, and intact proviral DNA assay

Exa-band PCR

To detect excision events, amplicons spanning the Ψ and Gag cut sites were generated by nested PCR using flanking primers listed in Table S6. PCR was performed with the Terra PCR kit (Takara, Japan) using an initial input of 100 ng template DNA, with cycling conditions, primer concentrations, reagent volumes, and other reaction parameters set according to the manufacturer's instructions. Products were visualized by agarose gel electrophoresis and confirmed using Sanger sequencing (Azenta, South Plainfield, NJ, USA).

Near full-length PCR

SIV proviral DNA from 3D8 cells was amplified using a two-round hemi-nested long-range PCR strategy designed to generate NFL amplicons tagged with unique molecular indices (UMIs) for downstream nanopore sequencing. For round 1, reactions contained 100 ng template DNA in a 50 μL reaction consisting of 25 μL Platinum SuperFi II Master Mix, 18 μL nuclease-free water, 2 μL combined SIV-NFL-For and SIV-NFL-Rev primers (Table S6) at 250 nM each, and 5 μL DNA template. Round 1 cycling was performed with an initial denaturation at 98°C for 30 s, followed by 30 cycles of 98°C for 10 s, 60°C for 10 s, and 72°C for 210 s, with a final extension at 72°C for 300 s and hold at 4°C. PCR products were purified using Nanopore Ampure XP beads at a 0.8× bead-to-sample ratio and eluted in nuclease-free water. Round 2 PCR used 5 μL purified round 1 product in a 50 μL reaction containing 25 μL Platinum SuperFi II Master Mix, 19.8 μL nuclease-free water, 0.1 μL R2 forward ONT primer and 0.1 μL R2 reverse ONT primer (Table S6), each at 250 nM, and was amplified using the same thermocycling program. The round 1 primers targeted SIV proviral regions and introduced 16 basepair unique UMIs and synthetic priming sites, while the round 2 primers targeted these synthetic sites and added Oxford Nanopore-compatible overhangs.

All ddPCR was conducted using the Bio-Rad QX200 system (Bio-Rad Laboratories, Hercules, CA, USA) to detect SIV Pol, Vpr, SaCas9, gRNAs sequences, and TERT gene as a reference in tissue DNA samples. It was also performed to quantify the relative abundance of SIV Gag and Env genes, providing an indirect measurement of excision efficiency by the loss of Gag/Env signal ratio. The primers and probes for each region are listed in Table S6. Intact proviral DNA was detected using a validated SIV- specific IPDA protocol employing dual-target probes against Pol and Env genes to distinguish intact from defective or hypermutated viral genomes as previously described.[28]

Nanopore sequence analysis

Products from either the Exa-band PCRs or SIV-NFL were sequenced by nanopore long read sequencing using R14 chemistry with the Native Barcoding Kit (SQK-NBD114.24) and the Ligation Sequencing DNA Kit (SQK-LSK114) according to the manufacturer's instructions.

Products were collected into batches of 6 and sequenced for 12 h. Raw Pod5 files were duplex basecalled using dorado (v0.9.1) with the model duplex model dna_r10.4.1_e8.2_5khz_stereo@v1.3 and simplex model dna_r10.4.1_e8.2_400bps_sup@v5.0.0.

The SIV-NFL samples were subjected UMI consensus merging using a modified version of the Nextflow umi-pipeline-nf.[46] Three algorithmic refinements were introduced to better accommodate the structural diversity of lentiviral amplicons sequenced on Oxford Nanopore platforms. First, the read-length filter used to exclude spurious concatemers and truncated molecules was extended with a deletion-tolerant mode. In the original implementation, a read was classified as too short whenever its aligned query span fell below a fixed fraction (minimal_overlap) of the target amplicon length, which inadvertently discarded genuine reads carrying large internal deletions—a hallmark of defective genomes. Under the new mode, the short-read criterion was instead based on the breadth of reference coverage across the BED-defined amplicon window, so that a read harboring a large internal deletion but still spanning the full target locus was retained. Correspondingly, the long-read (concatemer) cutoff in this mode evaluated the aligned query span rather than the total read length, preventing intact long-insert reads from being mis-classified as concatemers. An additional parameter allowed the upper length threshold to be set explicitly when the amplicon geometry departs substantially from the default formula. Second, a length-stratified subclustering step was added downstream of UMI-tag-based grouping. After reads sharing a UMI sequence were consolidated into subclusters by pairwise edit distance, each subcluster was further partitioned according to amplicon length using single-linkage clustering, with membership controlled by either an absolute base-pair tolerance or a percentage difference. This refinement addresses the scenario in which intact and internally deleted viral genomes co-occur in the same UMI family—whether by UMI collision or by sharing edit-distance-similar tags—which would otherwise yield a chimeric consensus sequence that corresponds to neither biological molecule. By resolving these length-distinct molecules before polishing, each class was independently corrected and reported. The modified version is available at https://github.com/DamLabResources/umi-pipeline-nf-HIV, commit 19f7d47 was used for this study.

The raw reads in the case of Exa-band PCR or in the case of SIV-NFL, the UMI consensus reads were subjected to a set of custom python scripts to characterize the structural outcomes of CRISPR editing in individual lentiviral genomes, each nanopore sequencing read was processed through a multi-stage classification pipeline. First, raw reads were aligned to the proviral reference sequence using minimap2 (v2.28) with an Oxford Nanopore-optimized preset, producing a set of primary and supplementary alignment segments per read. Because lentiviral long terminal repeats (LTRs) are nearly identical at both ends of the provirus, an automated self-alignment procedure tiles the reference with overlapping seed windows to precisely define the 5′ and 3′ LTR boundaries, and any supplementary alignments incorrectly placed at the wrong LTR copy are detected and re-aligned locally to the correct copy. Nanopore duplex reads—in which two complementary strands are sequenced in tandem and produce a single concatenated signal—were detected by their characteristic reference topology and split into their two constituent strand-level events so they do not appear as spurious structural rearrangements. Alignment segments mapping outside the expected amplicon window were then discarded. With the normalized alignment in hand, each read's CIGAR strings and inter-segment junctions were parsed to enumerate all structural events: small insertions and deletions, large intra-molecular deletions, inter-contig translocations, inversions, and circular reference wraps. Each event was annotated with its nearest CRISPR guide RNA (gRNA) cut site, and small indels falling within a defined window around each cut are recorded as target-site edits. Based on the combination of events present, every read was assigned one of seven mutually exclusive summary classes: *clean* (no edits or rearrangements), *indel at one or both sites* (small insertions or deletions at one or more cut sites but no large structural changes), *intended deletion* (a deletion spanning and bridging two distinct guide target sites, consistent with excision of the intervening sequence), *hemi-sided deletion* (a large structural rearrangement whose breakpoints map near a cut site), *unintended deletion* (a structural rearrangement whose breakpoints are not associated with any guide target), *mixed* (a combination of on- and off-target events in the same molecule), or no_alignment (the read failed to map). Each read was additionally flagged for whether it spans the complete expected genomic interval—a proxy for molecule integrity—and for its inferred topology (linear, circular, or multi-molecule concatemer).

Off-target analysis by rhAMP-Seq

Potential off-target sites for the Ψ_112 and Gag_468 guide pair were nominated using Excision BioTherapeutics' COTANA bioinformatic pipeline, which is based on Cas-OFFinder and incorporates a customized SaCas9 position-weight matrix. The rhesus macaque genome was searched using the SaCas9 PAM sequence 5′-NNGRRN-3′, allowing up to five mismatches and one bulge. Candidate sites were filtered and ranked based on mismatch number, bulge presence, PAM orientation, and predicted positional tolerance. A total of 49 nominated off-target sites were selected for analysis (Table S7).

rhAMP-Seq primer sets were designed using the IDT rhAMP primer design tool. Primers targeting the nominated off-target sites were pooled into a single primer set for the Ψ_112 and Gag_468 guide pair. Two rounds of PCR were performed according to the manufacturer's instructions, and PCR products from different samples were pooled at equal molar ratios before 2 × 150 bp paired-end sequencing on an Illumina NextSeq platform.

Sequencing data were analyzed using Excision BioTherapeutics' targeted amplicon sequencing pipeline. Reads were processed using FASTP for adapter, quality, and poly-G trimming. Insertions or deletions within ±5 bp of the predicted SaCas9 cut sites were quantified at each nominated off-target site. Structural variants were also evaluated. Statistical comparisons between treated and control samples were performed using a beta-binomial model, and power analysis was conducted to estimate the detection sensitivity at each site.

Amplicon barcode sequencing and clonal diversity analysis

Animals were infected with SIV tagged with a 34-nt cassette containing ten base pair (bp) random barcode sequences between the Vpx and Vpr genes. Viral barcode high-throughput sequencing was performed on the DNA prepared from the collected tissues. Regions flanking the barcoded sequences were amplified using nested PCR. Sequencing adapters were subsequently added in a third round of PCR using Illumina indexing primers (Table S6). Equal amounts of the barcoded PCR products were pooled and submitted to Azenta/Genewiz for 2 × 250 bp paired end deep sequencing.

Reads were analyzed using SIV barcode pipeline (adapted from https://github.com/DamLabResources/outerspace). First, demultiplexed R1 and R2 reads were merged using BBMerge. Reads that could not be merged were retained as single end reads. The merged FASTQ files were then converted to BAM format. For each merged or single end read, the 34-nt barcode was extracted by identifying 27 bp upstream and 26 bp downstream flanking sequences using a Python regular expression, allowing up to four mismatches (including a maximum of two insertions and deletions [indels]). Extracted barcodes were error-corrected and collapsed using UMI-tools (https://pmc.ncbi.nlm.nih.gov/articles/PMC5340976/), permitting up to three differences.[47] The resulting corrected barcodes were used to calculate barcode diversity (richness = number of unique barcode sequences post-correction) and for subsequent downstream analyses. To normalize richness, the values are adjusted based on the total number of reads and scaled to a standardized total of 1,000 reads.

Statistical analysis

Quantitative results are presented as mean ± standard deviation (SD). Group comparisons were performed using unpaired two-tailed student's t-tests or one-way ANOVA with Tukey's multiple comparison test, as appropriate. Statistical significance was defined as $p < 0.05$. Graphs were generated in GraphPad Prism version 10.0.

DATA AND CODE AVAILABILITY

The data supporting the findings of this study are available within the article and its supplemental materials. Additional data are available from the corresponding author upon reasonable request.

ACKNOWLEDGMENTS

The authors wish to thank past and present members of the Center for Neurovirology and Gene Editing for their support and sharing of reagents. We thank Dr. Maurizio Caocci from the Burdo laboratory for the preparation and distribution of brain tissues provided by our colleagues at BioQual, Inc.; Dr. Brandon Keele for providing the barcoded SIV; Dr. Yoelvis Garcia-Mesa and Dr. Jonathan Karn for kindly providing the M1A cells; and Merck and ViiV Healthcare for the antiretrovirals. We also thank the Duke Human Vaccine Institute for CSF viral loads (NIAID DAIDS Virology Core Laboratory; contract # 75N93025C0002), and the BioQual staff for animal care and experimental administration. We appreciate Cynthia Papaleo for the assembly of data and preparation of this manuscript. This research was supported by the CRISPR for Cure Martin Delaney Collaboratory for HIV cure UM1 AI164568 and co-funded by NIAID, NIMH, NIDA, NINDS, NIDDK, and NHLBI. This study utilized services offered by core facilities of the Comprehensive NeuroHIV Center (CNHC NIMH grant number P30MH092177).

AUTHOR CONTRIBUTIONS

Conceived the idea and supervised editing strategy: K.K.; construction of gRNAs and other reagents: C.C.; designed the editing experiments: K.K.; design of viral infection studies, communication with animal facility, tissue preparation and distribution: T.H.B.; performed the experiments: H.L., S.L., Z.S., W.H., and Y.H.; performed animal experiments: M.L. and C.A.L. (Bioqual facility); interpret the data: K.K., T.H.B., J.G., W.D., M.R.N., and B.W.; preparation and comments of manuscript: H.L., K.K., I.K.S., J.G., W.D., M.R.N., and B.W.; preparation of data and figures: A.R., H.L., I.K.S., W.D., and M.R.N. SIV sequencing and analysis: M.E.C., J.M.L., W.D., M.R.N., and B.W.

DECLARATION OF INTERESTS

The authors H.L., S.L., C.C., Z.S., A.R., Y.H., I.K.S., M.E.C., J.M.L., W.D., M.R.N., B.W., M.L., and C.A.L. declare no conflict of interest. W.H. and J.G. were employees of Excision BioTherapeutics. K.K. is named inventor on patents that cover the viral gene editing technology that is the subject of this article.

SUPPLEMENTAL INFORMATION

Supplemental information can be found online at https://doi.org/10.1016/j.omtn.2026.103063.

REFERENCES

1. Chun, T.W., Engel, D., Berrey, M.M., Shea, T., Corey, L., and Fauci, A.S. (1998). Early establishment of a pool of latently infected, resting CD4(+) T cells during primary HIV-1 infection. Proc. Natl. Acad. Sci. USA *95*, 8869–8873. https://doi.org/10.1073/pnas.95.15.8869.

2. Siliciano, J.M., and Siliciano, R.F. (2015). The remarkable stability of the latent reservoir for HIV-1 in resting memory CD4+ T cells. J. Infect. Dis. *212*, 1345–1347. https://doi.org/10.1093/infdis/jiv219.

3. Finzi, D., Hermankova, M., Pierson, T., Carruth, L.M., Buck, C., Chaisson, R.E., Quinn, T.C., Chadwick, K., Margolick, J., Brookmeyer, R., et al. (1997). Identification of a reservoir for HIV-1 in patients on highly active antiretroviral therapy. Science *278*, 1295–1300. https://doi.org/10.1126/science.278.5341.1295.

4. Wong, J.K., Hezareh, M., Günthard, H.F., Havlir, D.V., Ignacio, C.C., Spina, C.A., and Richman, D.D. (1997). Recovery of replication-competent HIV despite prolonged suppression of plasma viremia. Science *278*, 1291–1295. https://doi.org/10.1126/science.278.5341.1291.

5. Letendre, S.L., Ellis, R.J., Ances, B.M., and McCutchan, J.A. (2010). Neurologic complications of HIV disease and their treatment. Top. HIV Med. *18*, 45–55.

6. Edén, A., Fuchs, D., Hagberg, L., Nilsson, S., Spudich, S., Svennerholm, B., Price, R.W., and Gisslén, M. (2010). HIV-1 viral escape in cerebrospinal fluid of subjects on suppressive antiretroviral treatment. J. Infect. Dis. *202*, 1819–1825. https://doi.org/10.1086/657342.

7. González-Scarano, F., and Martín-García, J. (2005). The neuropathogenesis of AIDS. Nat. Rev. Immunol. *5*, 69–81. https://doi.org/10.1038/nri1527.

8. Glass, J.D., and Johnson, R.T. (1996). Human immunodeficiency virus and the brain. Annu. Rev. Neurosci. *19*, 1–26. https://doi.org/10.1146/annurev.ne.19.030196.000245.

9. Saylor, D., Dickens, A.M., Sacktor, N., Haughey, N., Slusher, B., Pletnikov, M., Mankowski, J.L., Brown, A., Volsky, D.J., and McArthur, J.C. (2016). HIV-associated neurocognitive disorder—pathogenesis and prospects for treatment. Nat. Rev. Neurol. *12*, 234–248. https://doi.org/10.1038/nrneurol.2016.27.

10. Ocampo, F.F. (2024). An overview of neurologic complications of HIV and opportunistic infections. Pract. Neurol. *23*, 28–34.

11. Wallet, C., De Rovere, M., Van Assche, J., Daouad, F., De Wit, S., Gautier, V., Mallon, P.W.G., Marcello, A., Van Lint, C., Rohr, O., and Schwartz, C. (2019). Microglial cells: The main HIV-1 reservoir in the brain. Front. Cell. Infect. Microbiol. *9*, 362. https://doi.org/10.3389/fcimb.2019.00362.

12. Wahl, A., and Al-Harthi, L. (2023). HIV infection of non-classical cells in the brain. Retrovirology *20*, 1. https://doi.org/10.1186/s12977-023-00616-9.

13. Torres-Muñoz, J., Stockton, P., Tacoronte, N., Roberts, B., Maronpot, R.R., and Petito, C.K. (2001). Detection of HIV-1 gene sequences in hippocampal neurons isolated from postmortem AIDS brains by laser capture

microdissection. J. Neuropathol. Exp. Neurol. *60*, 885–892. https://doi.org/10.1093/jnen/60.9.885.

14. Bagasra, O., Lavi, E., Bobroski, L., Khalili, K., Pestaner, J.P., Tawadros, R., and Pomerantz, R.J. (1996). Cellular reservoirs of HIV-1 in the central nervous system of infected individuals: Identification by the combination of in situ polymerase chain reaction and immunohistochemistry. AIDS *10*, 573–585. https://doi.org/10.1097/00002030-199606000-00002.

15. Liao, H.K., Gu, Y., Diaz, A., Marlett, J., Takahashi, Y., Li, M., Suzuki, K., Xu, R., Hishida, T., Chang, C.J., et al. (2015). Use of the CRISPR/Cas9 system as an intracellular defense against HIV-1 infection in human cells. Nat. Commun. *6*, 6413. https://doi.org/10.1038/ncomms7413.

16. Yin, C., Zhang, T., Qu, X., Zhang, Y., Putatunda, R., Xiao, X., Li, F., Xiao, W., Zhao, H., Dai, S., et al. (2017). In vivo excision of HIV-1 provirus by saCas9 and multiplex single-guide RNAs in animal models. Mol. Ther. *25*, 1168–1186. https://doi.org/10.1016/j.ymthe.2017.03.012.

17. Kaminski, R., Bella, R., Yin, C., Otte, J., Ferrante, P., Gendelman, H.E., Li, H., Booze, R., Gordon, J., Hu, W., and Khalili, K. (2016). Excision of HIV-1 DNA by gene editing: A proof-of-concept in vivo study. Gene Ther. *23*, 696. https://doi.org/10.1038/gt.2016.45.

18. Dash, P.K., Kaminski, R., Bella, R., Su, H., Mathews, S., Ahooyi, T.M., Chen, C., Mancuso, P., Sariyer, R., Ferrante, P., et al. (2019). Sequential LASER ART and CRISPR treatments eliminate HIV-1 in a subset of infected humanized mice. Nat. Commun. *10*, 2753. https://doi.org/10.1038/s41467-019-10366-y.

19. Foust, K.D., Nurre, E., Montgomery, C.L., Hernandez, A., Chan, C.M., and Kaspar, B.K. (2009). Intravascular AAV9 preferentially targets neonatal neurons and adult astrocytes. Nat. Biotechnol. *27*, 59–65. https://doi.org/10.1038/nbt.1515.

20. Mancuso, P., Chen, C., Kaminski, R., Gordon, J., Liao, S., Robinson, J.A., Smith, M.D., Liu, H., Sariyer, I.K., Sariyer, R., et al. (2020). CRISPR based editing of SIV proviral DNA in ART treated non-human primates. Nat. Commun. *11*, 6065. https://doi.org/10.1038/s41467-020-19821-7.

21. Burdo, T.H., Chen, C., Kaminski, R., Sariyer, I.K., Mancuso, P., Donadoni, M., Smith, M.D., Sariyer, R., Caocci, M., Liao, S., et al. (2024). Preclinical safety and biodistribution of CRISPR targeting SIV in non-human primates. Gene Ther. *31*, 224–233. https://doi.org/10.1038/s41434-023-00410-4.

22. Whitney, J.B., Hill, A.L., Sanisetty, S., Penaloza-MacMaster, P., Liu, J., Shetty, M., Parenteau, L., Cabral, C., Shields, J., Blackmore, S., et al. (2014). Rapid seeding of the viral reservoir prior to SIV viraemia in rhesus monkeys. Nature *512*, 74–77. https://doi.org/10.1038/nature13594.

23. Hatziioannou, T., and Evans, D.T. (2012). Animal models for HIV/AIDS research. Nat. Rev. Microbiol. *10*, 852–867. https://doi.org/10.1038/nrmicro2911.

24. Fennessey, C.M., Pinkevych, M., Immonen, T.T., Reynaldi, A., Venturi, V., Nadella, P., Reid, C., Newman, L., Lipkey, L., Oswald, K., et al. (2017). Genetically-barcoded SIV facilitates enumeration of rebound variants and estimation of reactivation rates in nonhuman primates following interruption of suppressive antiretroviral therapy. PLoS Pathog. *13*, e1006359. https://doi.org/10.1371/journal.ppat.1006359.

25. Yoshida, T.M., Nguyen, M., Zhang, L., Lu, B.Y., Zhu, B., Murray, K.N., Mineur, Y.S., Zhang, C., Xu, D., Lin, E., et al. (2025). The subfornical organ is a nucleus for gut-derived T cells that regulate behaviour. Nature *643*, 499–508. https://doi.org/10.1038/s41586-025-09050-7.

26. White, Z., Cabrera, I., Mei, L., Clevenger, M., Ochoa-Raya, A., Kapustka, I., Dominguez, J.R., Zhou, J., Koster, K.P., Anwar, S., et al. (2025). Gut inflammation promotes microbiota-specific CD4 T cell-mediated neuroinflammation. Nature *643*, 509–518. https://doi.org/10.1038/s41586-025-09120-w.

27. Spudich, S., and González-Scarano, F. (2012). HIV-1-related central nervous system disease: Current issues in pathogenesis, diagnosis, and treatment. Cold Spring Harb. Perspect. Med. *2*, a007120. https://doi.org/10.1101/cshperspect.a007120.

28. Fray, E.J., Wu, F., Simonetti, F.R., Zitzmann, C., Sambaturu, N., Molina-Paris, C., Bender, A.M., Liu, P.T., Ventura, J.D., Wiseman, R.W., et al. (2023). Antiretroviral therapy reveals triphasic decay of intact SIV genomes and persistence of ancestral variants. Cell Host Microbe *31*, 356–372.e5. https://doi.org/10.1016/j.chom.2023.01.016.

29. Ho, Y.C., Shan, L., Hosmane, N.N., Wang, J., Laskey, S.B., Rosenbloom, D.I.S., Lai, J., Blankson, J.N., Siliciano, J.D., and Siliciano, R.F. (2013). Replication-competent noninduced proviruses in the latent reservoir increase barrier to HIV-1 cure. Cell *155*, 540–551. https://doi.org/10.1016/j.cell.2013.09.020.

30. Bruner, K.M., Wang, Z., Simonetti, F.R., Bender, A.M., Kwon, K.J., Sengupta, S., Fray, E.J., Beg, S.A., Antar, A.A.R., Jenike, K.M., et al. (2019). A quantitative approach for measuring the reservoir of latent HIV-1 proviruses. Nature *566*, 120–125. https://doi.org/10.1038/s41586-019-0898-8.

31. Canestri, A., Lescure, F.X., Jaureguiberry, S., Moulignier, A., Amiel, C., Marcelin, A.G., Peytavin, G., Tubiana, R., Pialoux, G., and Katlama, C. (2010). Discordance between cerebral spinal fluid and plasma HIV replication in patients with neurological symptoms who are receiving suppressive antiretroviral therapy. Clin. Infect. Dis. *50*, 773–778. https://doi.org/10.1086/650538.

32. Maldarelli, F., Wu, X., Su, L., Simonetti, F.R., Shao, W., Hill, S., Spindler, J., Ferris, A.L., Mellors, J.W., Kearney, M.F., et al. (2014). Specific HIV integration sites are linked to clonal expansion and persistence of infected cells. Science *345*, 179–183. https://doi.org/10.1126/science.1254194.

33. Wagner, T.A., McLaughlin, S., Garg, K., Cheung, C.Y.K., Larsen, B.B., Styrchak, S., Huang, H.C., Edlefsen, P.T., Mullins, J.I., and Frenkel, L.M. (2014). Proliferation of cells with HIV integrated into cancer genes contributes to persistent infection. Science *345*, 570–573. https://doi.org/10.1126/science.1256304.

34. Liu, Y., Binda, C.S., Berkhout, B., and Das, A.T. (2023). CRISPR-Cas attack of HIV-1 proviral DNA can cause unintended deletion of surrounding cellular DNA. J. Virol. *97*, e0133423. https://doi.org/10.1128/jvi.01334-23.

35. Binda, C.S., Klaver, B., Berkhout, B., and Das, A.T. (2020). CRISPR-Cas9 dual-gRNA attack causes mutation, excision and inversion of the HIV-1 proviral DNA. Viruses *12*, 330. https://doi.org/10.3390/v12030330.

36. Samaranch, L., Salegio, E.A., San Sebastian, W., Kells, A.P., Foust, K.D., Bringas, J.R., Lamarre, C., Forsayeth, J., Kaspar, B.K., and Bankiewicz, K.S. (2012). Adeno-associated virus serotype 9 transduction in the central nervous system of nonhuman primates. Hum. Gene Ther. *23*, 382–389. https://doi.org/10.1089/hum.2011.200.

37. Hordeaux, J., Wang, Q., Katz, N., Buza, E.L., Bell, P., and Wilson, J.M. (2018). The neurotropic properties of AAV-PHP.B are limited to C57BL/6J mice. Mol. Ther. *26*, 664–668. https://doi.org/10.1016/j.ymthe.2018.01.018.

38. Rasmussen, T.A., and Lewin, S.R. (2016). Shocking HIV out of hiding: Where are we with clinical trials of latency reversing agents? Curr. Opin. HIV AIDS *11*, 394–401. https://doi.org/10.1097/COH.0000000000000279.

39. Søgaard, O.S., Graversen, M.E., Leth, S., Olesen, R., Brinkmann, C.R., Nissen, S.K., Kjaer, A.S., Schleimann, M.H., Denton, P.W., Hey-Cunningham, W.J., et al. (2015). The depsipeptide romidepsin reverses HIV-1 latency in vivo. PLoS Pathog. *11*, e1005142. https://doi.org/10.1371/journal.ppat.1005142.

40. Churchill, M.J., Deeks, S.G., Margolis, D.M., Siliciano, R.F., and Swanstrom, R. (2016). HIV reservoirs: What, where and how to target them. Nat. Rev. Microbiol. *14*, 55–60. https://doi.org/10.1038/nrmicro.2015.5.

41. Heaton, R.K., Clifford, D.B., Franklin, D.R., Jr., Woods, S.P., Ake, C., Vaida, F., Ellis, R.J., Letendre, S.L., Marcotte, T.D., Atkinson, J.H., et al. (2010). HIV-associated neurocognitive disorders persist in the era of potent antiretroviral therapy: CHARTER Study. Neurology *75*, 2087–2096. https://doi.org/10.1212/WNL.0b013e318200d727.

42. Ellis, R., Langford, D., and Masliah, E. (2007). HIV and antiretroviral therapy in the brain: Neuronal injury and repair. Nat. Rev. Neurosci. *8*, 33–44. https://doi.org/10.1038/nrn2040.

43. Josefsson, L., von Stockenstrom, S., Faria, N.R., Sinclair, E., Bacchetti, P., Killian, M., Epling, L., Tan, A., Ho, T., Lemey, P., et al. (2013). The HIV-1 reservoir in eight patients on long-term suppressive antiretroviral therapy is stable with few genetic changes over time. Proc. Natl. Acad. Sci. USA *110*, E4987–E4996. https://doi.org/10.1073/pnas.1308313110.

44. Sun, W., Rassadkina, Y., Gao, C., Collens, S.I., Lian, X., Solomon, I.H., Mukerji, S.S., Yu, X.G., and Lichterfeld, M. (2023). Persistence of intact HIV-1 proviruses in the brain during antiretroviral therapy. eLife *12*, RP89837. https://doi.org/10.7554/eLife.89837.

45. Garcia-Mesa, Y., Jay, T.R., Checkley, M.A., Luttge, B., Dobrowolski, C., Valadkhan, S., Landreth, G.E., Karn, J., and Alvarez-Carbonell, D. (2017). Immortalization of primary microglia: A new platform to study HIV regulation in the central nervous system. J. Neurovirol. *23*, 47–66. https://doi.org/10.1007/s13365-016-0499-3.

46. Karst, S.M., Ziels, R.M., Kirkegaard, R.H., Sørensen, E.A., McDonald, D., Zhu, Q., Knight, R., and Albertsen, M. (2021). High-accuracy long-read amplicon sequences using unique molecular identifiers with Nanopore or PacBio sequencing. Nat. Methods *18*, 165–169. https://doi.org/10.1038/s41592-020-01041-y.

47. Smith, T., Heger, A., and Sudbery, I. (2017). UMI-tools: modeling sequencing errors in Unique Molecular Identifiers to improve quantification accuracy. Genome Res. *27*, 491–499. https://doi.org/10.1101/gr.209601.116.

OMTN, Volume *37*

Supplemental information

In vivo genome editing of central nervous system SIV reservoirs in ART-suppressed rhesus macaques

Hong Liu, Shuren Liao, Chen Chen, Wenwen Huo, Zahra Safaei, Angela Rocchi, Yuru Huang, Ilker K. Sariyer, Mackenzie E. Collins, Jill M. Lawrence, Will Dampier, Michael R. Nonnemacher, Brian Wigdahl, Tricia H. Burdo, Jennifer Gordon, Mark Lewis, Cheri A. Lee, and Kamel Khalili

Supplemental Figures

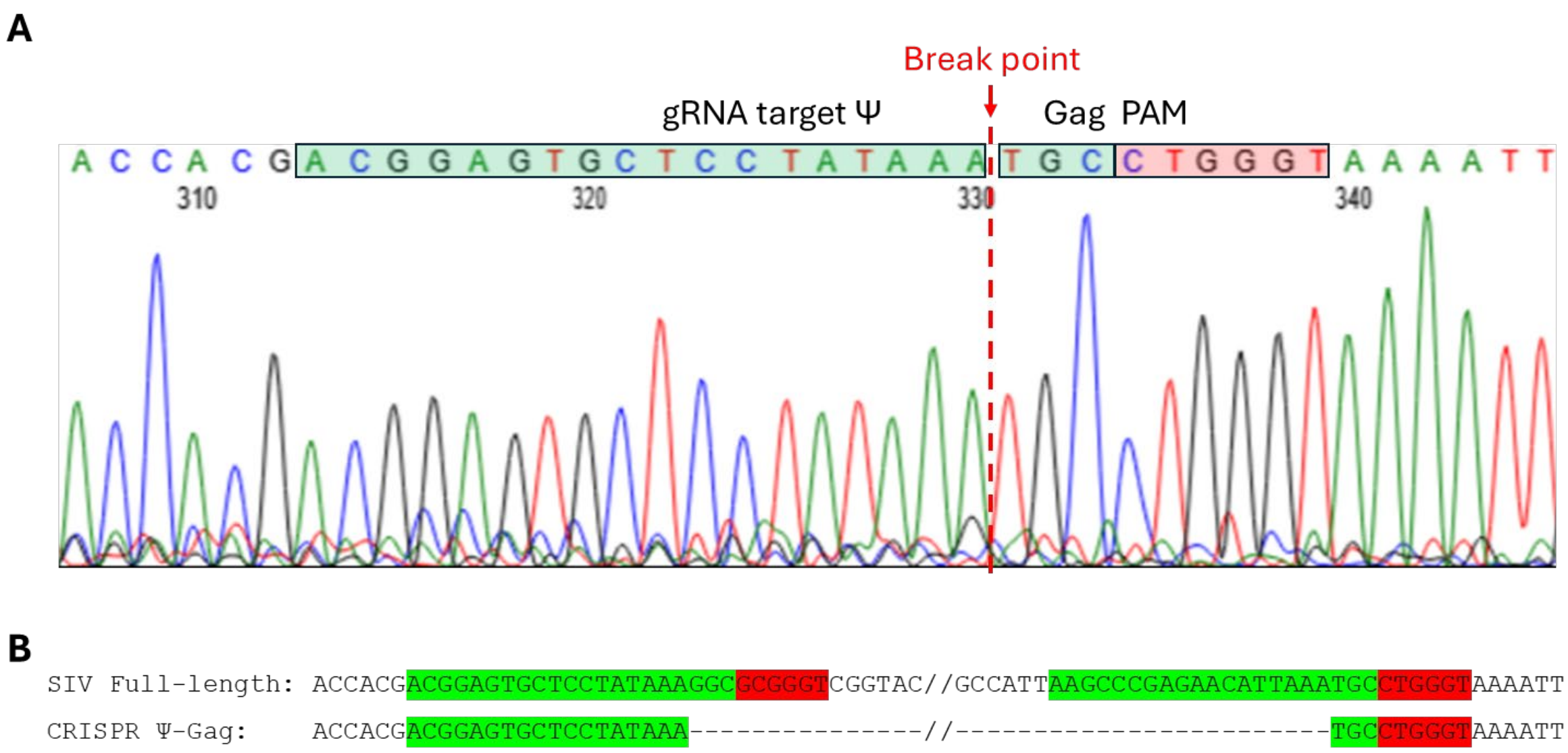

Figure S1. Sanger sequencing analysis of CRISPR-mediated excision of the SIV Ψ–Gag region in 3D8 cells. (A) Representative Sanger sequencing trace of the truncated amplicon generated by CRISPR cleavage and end joining within the SIV Ψ–Gag region in 3D8 cells. (B) Sequence alignment of the corresponding edited amplicons. The gRNA target sites are highlighted in green, and PAM sequences are shown in red.

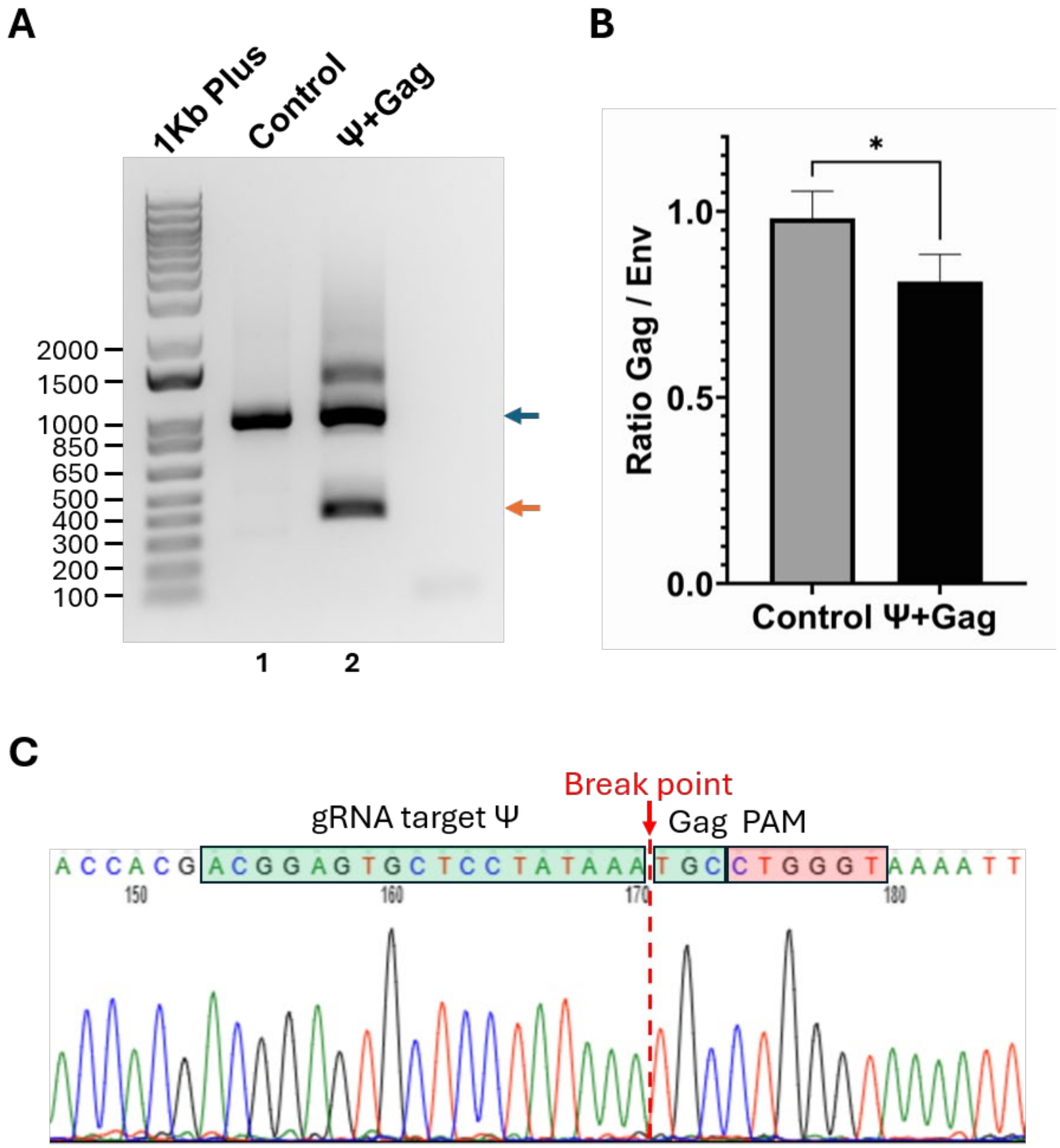

Figure S2. CRISPR-mediated excision of the Ψ–Gag region in SIVmac239-infected macaque microglial M1A cells. (A) PCR analysis of the Ψ–Gag region using genomic DNA isolated from SIVmac239-infected M1A macaque microglial cells electroporated with Cas9 RNPs containing both Ψ and Gag targeting gRNAs. Dual-gRNA editing produced a smaller amplicon corresponding to excision of the 590-bp Ψ–Gag segment. The orange arrow denotes the excision-specific band, whereas the blue arrow denotes the full-length band. (B) Quantification of excision efficiency by ddPCR. The reduced Gag-to-env signal ratio reflects deletion of the Gag segment in SIV-infected M1A cells. (C) Representative Sanger sequencing trace of the truncated amplicon indicated by the orange arrow in panel A, confirming CRISPR-mediated cleavage and end joining within the SIV Ψ–Gag region in M1A cells.

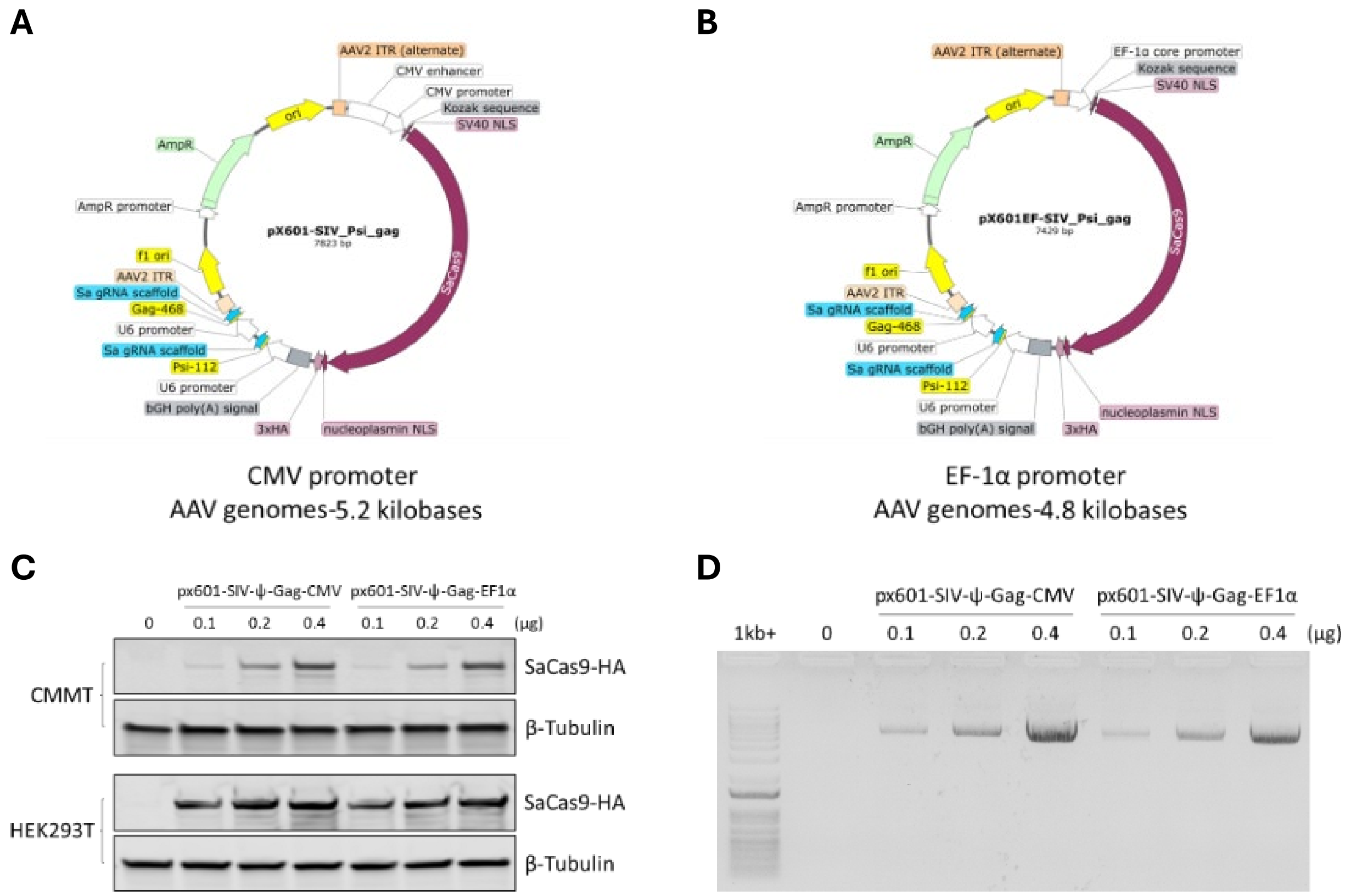

Figure S3. The EF1α core promoter maintains comparable SaCas9 expression while reducing vector size to improve AAV packaging. (A) Vector map of the pX601-SaCas9 construct containing dual gRNAs targeting SIV Ψ and Gag. The CMV promoter drives SaCas9 expression (red), and two gRNA cassettes (yellow target sequence + blue scaffold) encode guides directed against the SIV Ψ packaging signal and the Gag coding region. (B) Vector map of pX601-EF1α-SaCas9-SIV Ψ-Gag. In this construct, the CMV promoter was replaced with the EF1α core promoter to reduce vector genome size and improve compatibility with AAV9 packaging constraints. (C) Increasing amounts of px601-SIV-Ψ-Gag-CMV or px601-SIV-Ψ-Gag-EF1α plasmids were transfected into CMMT monkey cells or HEK293T human cells. SaCas9 expression levels were assessed by western blot analysis 72 hours post-transfection, with β-tubulin used as a loading control. (D) Equal amounts of plasmid DNA used in the transfection experiments were analyzed by agarose gel electrophoresis to confirm comparable DNA input between px601-SIV-Ψ-Gag-CMV and px601-SIV-Ψ-Gag-EF1α plasmids.

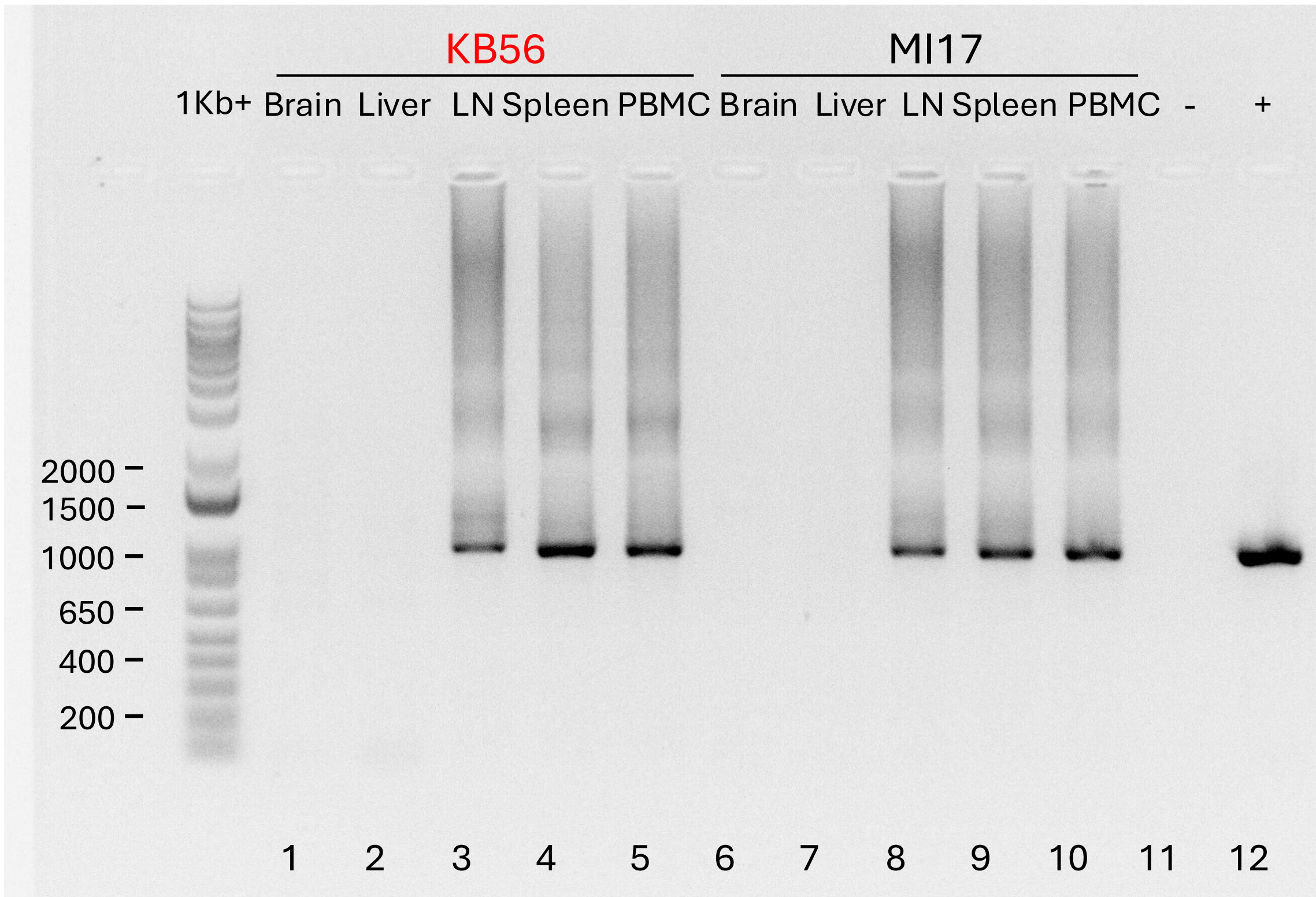

Figure S4. Nested PCR analysis of the SIV Pol region in tissues from ART-suppressed rhesus macaques. Agarose gel electrophoresis of nested PCR amplicons targeting the SIV Pol region showed products of similar size across tissues from both CRISPR-treated (KB56) and control (MI17) animals, with no truncated bands detected. Negative and positive controls consisted of water input and 3D8 cell genomic DNA, respectively.

A

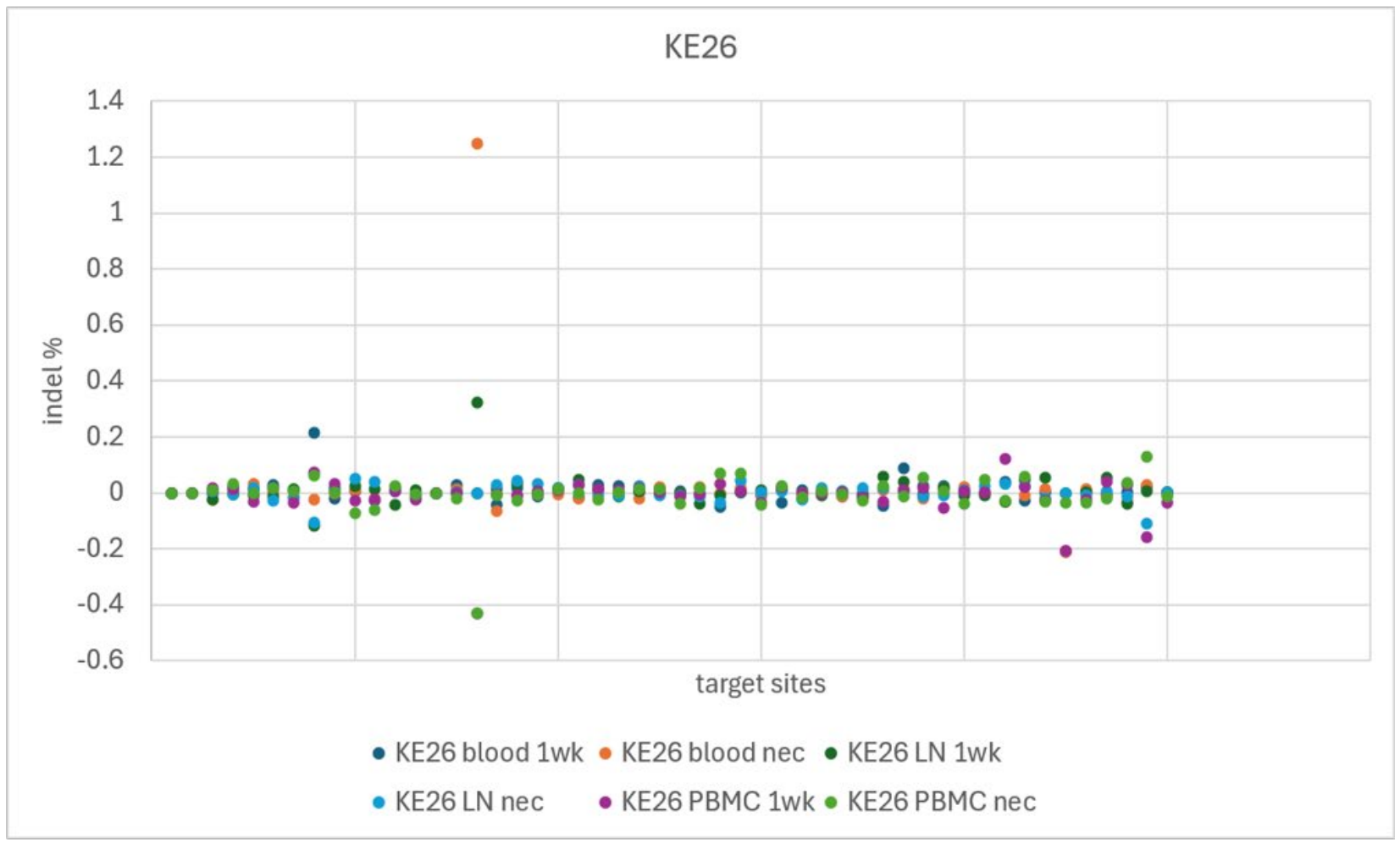

B

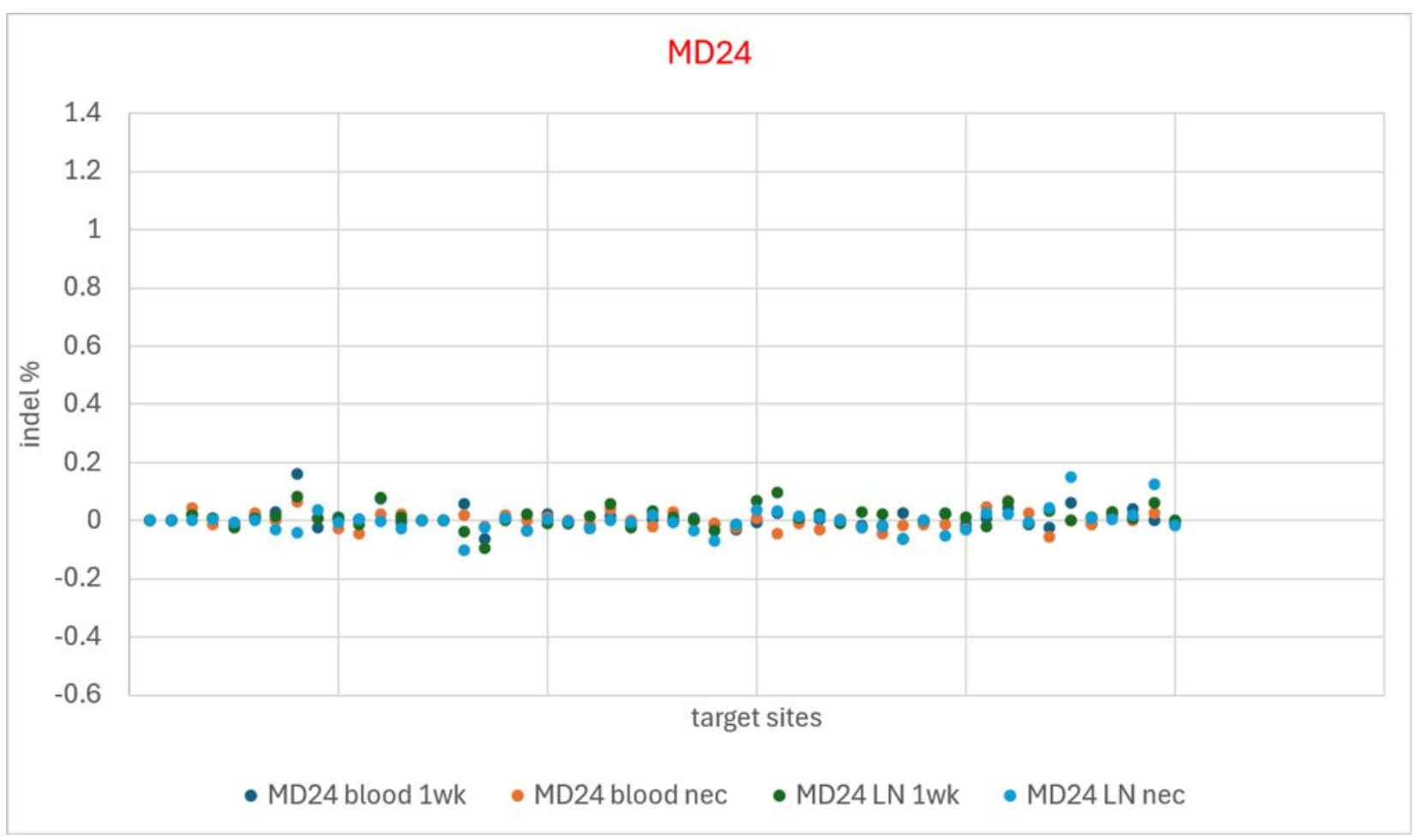

Figure S5. Delta indel frequencies at nominated off-target sites across regional tissue samples from two monkeys. For each nominated off-target site, the change in indel frequency (delta indel frequency) was calculated as the percentage of indel-containing reads in the post-CRISPR sample minus that in the matched pre-CRISPR baseline sample. Each dot represents one nominated off-target site in one tissue sample. Panel A shows Monkey KE26 (control), and panel B shows Monkey MD24 (treated). Distinct colors represent individual samples within each monkey. Off-target sites are arranged along the x-axis in the same order as listed in Table S7. Negative delta indel frequencies likely reflect technical variation resulting from unequal sequencing depth between baseline and post-CRISPR samples. At sites with relatively low baseline read depth, a single indel-containing read can disproportionately inflate the estimated background indel frequency, resulting in an artificial negative value after subtraction. These negative values should therefore be interpreted as baseline noise rather than evidence of reduced indel formation. Sequencing depth for all sites and samples is provided in Table S1. “1 wk” denotes samples collected 1 week after AAV9 treatment, and “nec” denotes samples collected at necropsy.

Supplemental Tables

Table S1. Aligned read depth at nominated off-target sites across regional tissue samples from two monkeys.

	KE26 blood			MD24 blood			KE26 LN cells			MD24 LN cells			KE26 PBMC		
Target Id	pre-	1 wk	Necro	pre-	1 wk	Necro	pre-	1 wk	Necro	pre-	1 wk	Necro	pre-	1 wk	Necro
Ψ112	0	0	0	0	0	0	1	0	2	0	1	2	1	0	0
Gag468	4	2	2	12	0	0	3	0	15	1	5	27	8	2	0
Gag468.188	32910	38839	33367	44180	47877	34770	37740	42495	26750	31947	45884	33430	40762	41429	13816
Gag468.1864	32480	39241	34483	44968	48201	38739	38923	44085	29428	37013	44976	39333	42942	41081	12766
Gag468.2361	25013	31394	25134	34899	36532	26772	27937	31884	18583	21949	32955	25627	29919	30894	8942
Gag468.2797	23138	26902	23081	30181	31085	23752	24199	27887	16843	18783	27785	20132	25001	26909	8291
Gag468.3388	22964	27920	23079	30918	32591	24176	24689	27430	16573	20349	27761	20911	26182	27441	8328
Gag468.3487	3291	3848	4042	2990	3852	3614	5672	6241	5703	5356	5140	5529	5189	5319	1695
Gag468.3875	32477	40040	34186	45423	47898	36981	40057	45222	27912	31205	46372	35957	42350	44344	13446
Gag468.4060	10975	13168	10778	14270	16071	10609	12033	13935	8356	9813	13767	11315	12620	14414	3972
Gag468.4398	12167	14301	12658	17194	17387	13553	13392	15714	9963	11281	15131	11643	15068	15538	5023
Gag468.7037	8175	10580	9061	12098	13140	10299	8852	10465	6964	8432	9735	9065	10244	11269	3119
Gag468.7156	15338	19174	16148	15020	15349	11819	17853	19698	14059	10713	14042	11663	19272	20924	6568
Gag468.7383	0	2	0	0	1	0	1	0	2	2	0	2	2	1	2
Gag468.8142	22697	27516	24880	195	147	156	25233	30364	19833	212	206	158	28350	29266	9935
Gag468.8966	199	250	160	1457	1546	1284	243	308	225	969	1519	1153	233	254	90
Gag468.9400	5826	8008	6605	8591	10266	6835	7522	7578	4951	5530	7515	6242	6905	7984	2501
Gag468.9813	16718	21875	17782	21403	22418	17700	18660	20403	14658	14549	16324	14636	21385	22236	6941
Gag468.10211	20363	25629	20097	12475	12914	8599	26206	29639	17482	8486	14372	10434	26944	29091	8644
Gag468.11495	37801	45480	37693	52462	55633	38773	46230	52592	33749	37073	54860	41880	47627	50431	16133
Gag468.11808	36377	42897	37607	49271	51170	39474	41175	46069	29671	34530	48418	35405	43680	45816	15633
Gag468.12030	18032	21399	18375	23826	24276	19445	21729	23385	15451	18621	22618	19750	21350	21189	6567
Gag468.12674	21219	26068	22085	26457	27957	20760	23567	26478	17819	17482	21229	19627	25871	26773	8571
Gag468.13583	22766	27416	24302	32130	34649	25676	24525	26290	18494	21914	26004	24435	28738	28355	8431
Gag468.14061	34426	42003	36746	46823	49186	37368	38925	44647	27752	31300	44746	33968	41461	42910	13924
Gag468.14698	27812	34015	29214	38522	41570	30601	30183	36124	22215	25423	36450	26906	33823	34685	11077
Gag468.14829	12028	14614	12708	16210	17168	13475	15682	17999	10592	14834	18779	15119	15561	15193	4275
Ψ112.461	10277	13336	10556	15411	17239	12705	12950	14327	8420	9849	15027	11865	13254	15444	4411
Ψ112.1278	24548	30467	27027	36135	37727	30569	27816	30832	20392	26320	32962	26740	31166	33118	10022
Ψ112.1523	21108	26375	21293	19379	21186	15198	23541	27236	17456	12210	17276	13878	25489	27063	8737
Ψ112.2484	11153	14017	11303	19382	21205	14553	13551	15462	9865	13065	19219	14250	13881	15530	5234
Ψ112.3154	12456	15293	13185	16743	19507	15107	14182	16070	10747	12065	16993	12436	15408	17387	5507
Ψ112.3637	30668	38238	32868	44833	46716	36214	33763	38878	25872	31959	37630	32808	38276	40368	12542
Ψ112.3900	16332	20951	17270	23896	26372	19119	18296	22039	14078	17590	22507	17815	20748	23318	7459
Ψ112.4440	35011	42636	37627	47104	49763	39063	41237	47524	30486	34097	48758	36020	42206	46170	16002
Ψ112.4469	32911	39176	33783	44187	48623	34805	36779	40571	25138	30330	39755	32537	40016	39528	12331
Ψ112.4645	7123	8324	7173	9456	10106	7820	11042	12181	8857	10699	13948	10379	11417	11159	3931
Ψ112.5896	37024	43677	37808	48447	50211	39922	39139	44391	27679	31975	43827	36048	44072	42769	13015
Ψ112.6825	13797	17324	14355	20283	21964	15764	15932	18985	11147	14171	20275	14599	17963	19737	5573
Ψ112.6971	31170	38240	33173	42768	44208	33682	37032	42175	25794	29849	42698	31926	39725	41406	12876
Ψ112.7215	12297	15685	12244	19316	21222	14201	14850	16878	11064	12620	18029	14379	15802	17303	5132
Ψ112.7741	3229	3816	3239	4214	4455	3224	3640	4177	2247	2466	3818	3098	3539	3996	1153
Ψ112.8341	37173	45199	35595	53001	56886	40484	37378	42681	28123	33755	43291	38673	45753	43149	12591
Ψ112.8946	11696	14316	12275	16581	17789	13593	13600	15432	10330	10776	15547	12426	14680	14792	5123
Ψ112.9260	1420	1450	1488	1642	1610	1590	1915	2264	1905	2774	2403	2009	1946	1516	584
Ψ112.9911	34856	41623	35725	48406	49925	38762	39558	45440	29714	34763	46791	35851	43655	45894	15299
Ψ112.10613	14275	16410	14123	17849	19927	15627	14939	16202	10298	12073	16686	12774	15276	16445	4983
Ψ112.10754	11215	13983	13297	16256	18192	15757	15227	17161	11310	14459	19560	14985	15872	16603	5410
Ψ112.10978	10179	13020	9748	14661	15921	9862	11761	14032	7829	9598	14459	11168	14587	14173	3256
Ψ112.11009	19083	23876	19903	26534	30260	22499	21484	24871	16857	18993	24084	19908	23576	26296	8838
ACTB	2291	2537	2904	2984	2761	3267	5138	5663	4870	7806	7463	6440	4303	3185	1156
GAPDH	4940	5502	5553	6513	6369	6494	8142	8621	6264	8874	10260	8877	7103	6527	2056

Table S2. Summarizing the positive ddPCR signaling for SIV Pol DNA in different brain regions from the 10 animals.

SIV POL detected by ddPCR	KE26	MD24	JM96	KK68	JV38	MJ21	MC43	MN45	KB56	MI17
Prefrontal Cortex	X	X	X	X						X
Frontal Cortex	X									X
Basal Ganglia										X
Caudate Nuclei	X						X			
Parietal Cortex		X							X	
Hippocampus			X				X	X		
Temporal Cortex						X		X		
Occipital Cortex			X						X	X
Hypothalamus	X					X				
Substantia Nigra			X					X		X
Choroid Plexus			X	X	X	X	X			X
Cerebellum					X					X
Brain Stem								X	X	

Table S3. Tissue distribution of intact proviral DNA (copies per 150 ng gDNA) in 10 rhesus macaques.

SIV IPDA	KE26	MD24	JM96	KK68	JV38	MJ21	MC43	MN45	KB56	MI17
Liver	0.00	0.00	0.00	0.00	1.50	0.00	6.48	0.00	0.00	0.00
Submandibular LN	40.11	26.27	40.14	34.73	11.15	3.02	10.54	1.60	11.20	6.00
Spleen cells	63.67	826.75	9.63	3.03	65.31	10.11	4.27	2.57	1.37	4.07
PBMC	5.28	0.00	NA	1.55	4.80	3.32	0.00	4.38	3.34	124.53
Prefrontal Cortex	0.00	0.00	0.00	0.00	8.00	0.00	0.00	0.00	0.00	2.76
Frontal Cortex	0.00	0.00	0.00	0.00	1.80	0.00	0.00	0.00	0.00	0.00
Basal Ganglia	0.00	0.00	1.40	0.00	0.00	0.00	0.00	0.00	0.00	0.00
Caudate Nuclei	0.00	0.00	0.00	0.00	0.00	0.00	0.00	0.00	0.00	0.00
Parietal Cortex	0.00	0.00	0.00	0.00	0.00	0.00	0.00	0.00	0.00	0.00
Hippocampus	0.00	0.00	0.00	0.00	5.85	0.00	0.00	2.60	0.00	0.00
Temporal Cortex	0.00	0.00	0.00	0.00	2.00	1.20	0.00	0.00	0.00	0.00
Occipital Cortex	0.00	0.00	0.00	9.61	0.00	0.00	0.00	0.00	0.00	1.28
Hypothalamus	1.20	0.00	0.00	0.00	0.00	0.00	0.00	1.20	0.00	0.00
Substantia Nigra	0.00	1.40	0.00	0.00	0.00	0.00	0.00	0.00	0.00	0.00
Choroid Plexus	0.00	0.00	0.00	0.00	0.00	0.00	0.00	0.00	0.00	0.00
Cerebellum	0.00	0.00	0.00	0.00	0.00	0.00	0.00	0.00	0.00	0.00
Brain Stem	0.00	0.00	0.00	1.40	0.00	0.00	0.00	0.00	0.00	0.00
Sciatic Nerve	0.00	0.00	0.00	0.00	0.00	0.00	0.00	3.00	0.00	0.00
Trigeminal Ganglia	0.00	0.00	10.20	0.00	0.00	1.35	0.00	0.00	0.00	0.00
Thoracic DRG	1.22	NA	0.00	0.00	0.00	0.00	0.00	0.00	0.00	0.00
Lumbar DRG	0.00	0.00	0.00	1.40	0.00	0.00	0.00	0.00	0.00	0.00
Thoracic Spinal Cord	0.00	1.26	0.00	0.00	0.00	0.00	0.00	3.47	0.00	0.00
Lumbar Spinal Cord	0.00	0.00	0.00	0.00	0.00	0.00	0.00	0.00	0.00	0.00

NA= Not available

Table S4. Longitudinal CSF viral load measurements (RNA copies/mL, * indicates 1 out of 2 replicates detected).

Sample ID	Before ART	2 weeks after ART	2 months after ART	Necropsy
**KE26**	NA	9,457	Not Detected	169
**MD24**	23,578	12,455	Not Detected	Not Detected
**JM96**	280,390	3,824	Not Detected	Not Detected
**KK68**	47,514	67	Not Detected	Not Detected
**JV38**	24,412	2,848	232*	790
**MJ21**	284,815	466	76*	45*
**MC43**	12,759	1,681	Not Detected	68
**MN45**	293,687	229	Not Detected	38
**KB56**	22,561	77*	Not Detected	Not Detected
**MI17**	173,323	623	119*	37

NA= Not available

The limit of quantification (LOQ) for this assay is 62 RNA cp/mL (1.79 log10) with 0.5 mL plasma. Values below the LOQ may be outside of the 95% confidence interval for reproducibility.

Table S5. SIV barcode and gRNAs sequences.

**Barcode**	
vpx-barcode-vpr	-ACGCGAGCCATGNNNNNNNNNNCGATGCGCGCGT- or<br>-ACGCGCGCATCGNNNNNNNNNNCATGGCTCGCGT-
**SIV gRNA PAM**	
Ψ_112	ACGGAGTGCTCCTATAAAGGCGCGGGT
Gag_468	AAGCCCGAGAACATTAAATGCCTGGGT

Table S6. Primer sequences.

SIV GAG F	GCTCCATTAGTGCCAACAGG
SIV GAG R	ACCACTAGGTGTCTCTGCAC
SIV GAG P FAM	TGCGTCATCTGGTGCATTCACGCA
SIV ENV F	CTACTGGTGGCACCTCAAGA
SIV ENV R	AGCGGTCAGCGTCAACGA
SIV ENV P HEX	TGTGCTAGGGTTCTTGGGTT
SIV POL F	TTCTGCCTCAGGGATGGAAG
SIV POL R	CCCTGTCATGTTCCAGGTCT
SIV POL P FAM	TTGCCTTCCTGAAGGGTTCTAGCACA
SIV VPR F	AAGGACCACAAAGGGAACCA
SIV VPR R	CCTCAAGGGTGTCTCCATGT
SIV VPR P FAM	CCTCGCTTGCTAACTGCACTTGGT
RM TERT F	GAGCTGAGATTGTGCCCTTG
RM TERT R	CCATTTGCTGCTCTCTGCTC
RM TERT P HEX	CCAGCACAGATCCTGGTCCCGT
Ψ gRNA F	GACGGAGTGCTCCTATAAAGGC
GAG gRNA F	GAAGCCCGAGAACATTAAATGC
Scaffold R	CGCCAACAAGTTGACGAGAT
Scaffold P FAM	CAGAATCTACTAAAACAAGGCAAA
SaCas9 F	CGACATCAAGGACATTACCG
SaCas9 R	GCTGCTCTGGTAGATGGTCA
SaCas9 P FAM	AACGCCGAGCTGCTGGATCA
IPDA POL F	GCAGGGATAGAGCACACCTTTG
IPDA POL R	CTATGGTTTCTACTGAATTTGCTTGTTC
IPDA POL P	TTTCAGGTGGTGATTCA
IPDA POL P	TAGGTGGTGATTTATT
IPDA ENV F	CCTCAATAAAGCCTTGTGTAAAATTATC
IPDA ENV R	GTTGTTGATGATTTTGTCAATCCC
IPDA ENV P HEX	TGCATTACTATGAGATGC
IPDA ENV P	TGCATTACTATAAAATGC
Excision 1st F	GCTAGACTCTCACCAGCACT
Excision 1st R	CTGATCCTGACGGCTCCCTA
Excision Nested	CGCCTGGTCAACTCGGTACTC
Excision Nested	TCTGCAGCCTCCTCGTTTAT
Barcode 1st F	GGAGAAGAGACAATAGGAGAGG
Barcode 1st R	GAGCGCTCGTTGGAGGATTC
Barcode	CTACACGACGCTCTTCCGATCT AAACCACCTACCAAGGGAGC
Barcode	AGACGTGTGCTCTTCCGATCT CCTCAAGGGTGTCTCCATGT
SIV-NFL-For	CAAGCAGAAGACGGCATACGAGATNNNYRNNNYRNNNYRNNNatatacttagaaaaggaagaaggcatcata
SIV-NFL-Rev	AATGATACGGCGACCACCGAGATCNNNYRNNNYRNNNYRNNNgccttagccttttcggagttcttc
OntAdapter-For	TTTCTGTTGGTGCTGATATTGCCAAGCAGAAGACGGCATACGAGAT
OntAdapter-	ACTTGCCTGTCGCTCTATCTTCAATGATACGGCGACCACCGAGATC

i501	AATGATACGGCGACCACCGAGATCTACACTATAGCCTACACTCTTTCCCTACACGACGCTCTTCCGATC
i502	AATGATACGGCGACCACCGAGATCTACACATAGAGGCACACTCTTTCCCTACACGACGCTCTTCCGAT
i503	AATGATACGGCGACCACCGAGATCTACACCCTATCCTACACTCTTTCCCTACACGACGCTCTTCCGATC
i504	AATGATACGGCGACCACCGAGATCTACACGGCTCTGAACACTCTTTCCCTACACGACGCTCTTCCGAT
i505	AATGATACGGCGACCACCGAGATCTACACAGGCGAAGACACTCTTTCCCTACACGACGCTCTTCCGAT
i506	AATGATACGGCGACCACCGAGATCTACACTAATCTTAACACTCTTTCCCTACACGACGCTCTTCCGATC
i507	AATGATACGGCGACCACCGAGATCTACACCAGGACGTACACTCTTTCCCTACACGACGCTCTTCCGAT
i508	AATGATACGGCGACCACCGAGATCTACACGTACTGACACACTCTTTCCCTACACGACGCTCTTCCGATC
i509	AATGATACGGCGACCACCGAGATCTACACGACGACCTACACTCTTTCCCTACACGACGCTCTTCCGATC
i510	AATGATACGGCGACCACCGAGATCTACACTAATCGGCACACTCTTTCCCTACACGACGCTCTTCCGATC
i511	AATGATACGGCGACCACCGAGATCTACACTACAGCCTACACTCTTTCCCTACACGACGCTCTTCCGATC
i512	AATGATACGGCGACCACCGAGATCTACACTATAATGAACACTCTTTCCCTACACGACGCTCTTCCGATC
i513	AATGATACGGCGACCACCGAGATCTACACCAGGCGAGACACTCTTTCCCTACACGACGCTCTTCCGAT
i514	AATGATACGGCGACCACCGAGATCTACACCATGGCTAACACTCTTTCCCTACACGACGCTCTTCCGATC
i515	AATGATACGGCGACCACCGAGATCTACACATGAGCGTACACTCTTTCCCTACACGACGCTCTTCCGAT
i516	AATGATACGGCGACCACCGAGATCTACACGGTAGCACACACTCTTTCCCTACACGACGCTCTTCCGAT
i701	CAAGCAGAAGACGGCATACGAGATACCGTGATGTGACTGGAGTTCAGACGTGTGCTCTTCCG
i702	CAAGCAGAAGACGGCATACGAGATACACATCGGTGACTGGAGTTCAGACGTGTGCTCTTCCG
i703	CAAGCAGAAGACGGCATACGAGATACGCCTAAGTGACTGGAGTTCAGACGTGTGCTCTTCCG
i704	CAAGCAGAAGACGGCATACGAGATACTGGTCAGTGACTGGAGTTCAGACGTGTGCTCTTCCG
i705	CAAGCAGAAGACGGCATACGAGATACCACTGTGTGACTGGAGTTCAGACGTGTGCTCTTCCG
i706	CAAGCAGAAGACGGCATACGAGATACATTGGCGTGACTGGAGTTCAGACGTGTGCTCTTCCG
i707	CAAGCAGAAGACGGCATACGAGATACGATCTGGTGACTGGAGTTCAGACGTGTGCTCTTCCG
i708	CAAGCAGAAGACGGCATACGAGATACTCAAGTGTGACTGGAGTTCAGACGTGTGCTCTTCCG
i709	CAAGCAGAAGACGGCATACGAGATACCTGATCGTGACTGGAGTTCAGACGTGTGCTCTTCCG
i710	CAAGCAGAAGACGGCATACGAGATACAAGCTAGTGACTGGAGTTCAGACGTGTGCTCTTCCG
i711	CAAGCAGAAGACGGCATACGAGATACGTAGCCGTGACTGGAGTTCAGACGTGTGCTCTTCCG
i712	CAAGCAGAAGACGGCATACGAGATACTACAAGGTGACTGGAGTTCAGACGTGTGCTCTTCCG
i713	CAAGCAGAAGACGGCATACGAGATACTTGACTGTGACTGGAGTTCAGACGTGTGCTCTTCCG
i714	CAAGCAGAAGACGGCATACGAGATACGGAACTGTGACTGGAGTTCAGACGTGTGCTCTTCCG
i715	CAAGCAGAAGACGGCATACGAGATACTGACATGTGACTGGAGTTCAGACGTGTGCTCTTCCG
i716	CAAGCAGAAGACGGCATACGAGATACGGACGGGTGACTGGAGTTCAGACGTGTGCTCTTCCG

Table S7. Genomic coordinates of nominated predicted off-target sites assayed by rhAmpSeq.

Guide Name	DNA	Chr.	Dir.	Location	score	Mismatch	Bulge
Gag468.188	AAGagaGAGAACATTAgATGCCCGAGT	chr8	+	129646327	3.81	4	0
Gag468.1864	AAGaCCGtcAACATTAAATGCTGGAGA	chr8	+	74466804	3.15	4	0
Gag468.2361	AAGCCACcAGAAaATTAAgTGCCAGAAT	chr5	+	142783769	4.78	3	1
Gag468.2797	AAG-CCtAGAACATTAtATGCAAGGAA	chr6	+	33186275	5.17	3	1
Gag468.3388	AAGa-CaAGAACATTAcATGCATGAAT	chr1	-	68264642	3.84	3	1
Gag468.3487	cAcCCC-AGAACATaAAATGCTGGAGT	chr1	-	90854215	2.25	3	1
Gag468.3875	AAGCCCaATGAcCATTAcATGCTGGGAT	chr1	-	165565036	3.94	3	1
Gag468.4060	AtaCtaGA-AACATTAAATGCACGAAT	chrX	+	7478665	2.39	4	1
Gag468.4398	AAGaCtG-GcAgATTAAATGCCAGAAT	chrX	-	62915993	2.13	4	1
Gag468.7037	AAcCCCaAGATACATTAgATGCCGGAGT	chr1	+	212407024	3.69	3	1
Gag468.7156	AAGtCaGAGAcCATTcAATGCAGGAAT	chr10	-	14701398	2.74	4	0
Gag468.7383	tAGCCgGgGcACATaAAATGCTGGGGT	chr10	+	63679751	2.17	5	0
Gag468.8142	AgGCaCGtGAggATTAAATGCAAGAAT	chr13	-	30597702	2.22	5	0
Gag468.8966	AAtCaaGAGAcCATTAAATGCCAGAAT	chr14	+	76605106	1.49	4	0
Gag468.9400	AAGCaaGAG-ACAcTAAATGCCAGGGT	chr15	+	39545550	2.82	3	1
Gag468.9813	AAGaCgG-GAACAaTAAATGCTGGGGT	chr16	+	6018724	2.18	3	1
Gag468.10211	AAGaCaGAGAtCATTAAATGtAAGGAT	chr17	+	15401964	6.84	4	0
Gag468.11495	AAG-CtGAGAACAaTAAAaGCGTGGGT	chr2	-	30837452	5.95	3	1
Gag468.11808	AAGCACatAGAACATTAAATGCTAGAGA	chr2	-	96069761	3.75	3	1
Gag468.12030	tAtCaCaAGAA-ATTAAATGCAGGAGT	chr2	-	140752403	1.82	4	1
Gag468.12674	AAaCCCcAGAACA-TAAAgGCAAGGGT	chr20	-	71565886	5.95	3	1
Gag468.13583	AAGCCCcAGgAaATTAAATGCAAGGAC	chr3	-	167015911	3.26	4	0
Gag468.14061	AtGgttGAGAAgATTAAATGCCAGGAT	chr4	+	69816156	2.33	5	0
Gag468.14698	AAG-gCaAGAACATaAAATGCAAGGAT	chr5	-	21133450	2.84	3	1
Gag468.14829	tAGtaCaAGAtCATTAAATGCTTGAGT	chr5	+	50382941	1.63	5	0
Ψ112.461	tCtGAGTGCTCC-ATAAAtGCAGGGAT	chr13	+	13583359	5.54	3	1
Ψ112.1278	gCtGgGTGCTgtTATAAAGGCATGGAT	chr10	+	79479345	2.1	5	0
Ψ112.1523	AaaaAcTcCTCCTATAAAGGCGTGGGT	chr11	+	42072377	1.82	5	0
Ψ112.2484	cCaGAGccCTCaTATAAAGGCTGGAAT	chr9	+	73036870	1.87	5	0
Ψ112.3154	cCtGgGT-CTCCgATAAAGGCTGGAGT	chr1	-	38951803	1.94	4	1
Ψ112.3637	ACtGAGTGCcCCTA-AgAGGCAGGGAT	chr1	-	172494503	4.59	3	1
Ψ112.3900	AgGGAGTtCTCC-ATAcAGGCTGGGAT	chr1	+	220144280	3.96	3	1
Ψ112.4440	tgGcAGTGC-CCTATAAAGGCCTGAGT	chr14	-	49910403	1.73	3	1
Ψ112.4469	gCacA-TGCTCCcATAAAGGCCAGGAT	chr14	-	55638045	2.38	4	1
Ψ112.4645	AacGAaTGCATtCTATAAAGGCAGGGGT	chr14	+	104654523	2.41	4	1
Ψ112.5896	ACtGctTcCTCtTATAAAGGCAAGAAT	chr2	+	112791194	1.92	5	0
Ψ112.6825	AtGGAG-GCTCCTATAAAGtCGTGGGT	chr3	+	63712084	5.84	2	1
Ψ112.6971	AtGaAG-GCTCCTATAAAGtCAGGAAT	chr3	+	94988327	5.99	3	1
Ψ112.7215	ACGTcAaTGCTCCTATAAAGGCAAGAAG	chr3	-	166373433	3.67	3	1
Ψ112.7741	ACtGtGaGCTCCT-TAAAGGCAGGAAT	chr6	-	42502495	2.12	3	1
Ψ112.8341	A-GGccTGaTCCTATAAAGGCATGAAT	chr7	-	20912766	1.85	5	0
Ψ112.8946	AgGGAGTGCaCCTcTAAAGGCTTGAAG	chr18	-	4656415	3.57	4	0
Ψ112.9260	AgGGAGATGCTtCTATAAgGGCATGAAT	chr19	+	9683101	4.51	3	1
Ψ112.9911	ACtGtGTGCTCCT-TgAAGGCAGGGAT	chr4	+	165529910	3.81	3	1
Ψ112.10613	ACaGAGTG-TCCTATAAtGGCAAGAAC	chr7	-	79468216	5.91	3	1
Ψ112.10754	AaGGAGTGaTCCTATAAgGaCCTGGGT	chr7	-	114225839	8.39	4	0
Ψ112.10978	ACaGAGT-CTCCaAaAAAGGCTGGAGT	chr7	+	156975163	2.97	3	1
Ψ112.11009	ACaGAGaGCTCCT-TcAAGGCAGGAAT	chr7	-	163600501	3.85	3	1
Ψ112.8536	cCtGAGgGCcCCTAgAAAGGCAAGGGT	chr16	+	72658083	2.09	5	0

Nominated off-target sites for the psi and gag guide RNAs are listed with their chromosomal coordinates. Sites are presented in the same order as displayed along the x-axis of Figure S5. The predicted sites Ψ112.8536 is not assayed, as no primers could be generated using the IDT rhAmpSeq design tool.
